# Supplementary material for: Human vascularized macrophage-islet organoids to model immune-mediated pancreatic β cell pyroptosis upon viral infection
Source: Cell Stem Cell. 2024 Nov 7;31(11):1612–1629.e8. doi: 10.1016/j.stem.2024.08.007 (PMC11546835; doi:10.1016/j.stem.2024.08.007)
Supplement: Document S1. Figures S1–S6 and Tables S1–S3 [file mmc1.pdf]

Cell Stem Cell, Volume *31*

Supplemental Information

Human vascularized macrophage-islet organoids to model immune-mediated pancreatic β cell pyroptosis upon viral infection

Liuliu Yang, Yuling Han, Tuo Zhang, Xue Dong, Jian Ge, Aadita Roy, Jiajun Zhu, Tiankun Lu, J. Jeya Vandana, Neranjan de Silva, Catherine C. Robertson, Jenny Z. Xiang, Chendong Pan, Yanjie Sun, Jianwen Que, Todd Evans, Chengyang Liu, Wei Wang, Ali Naji, Stephen C.J. Parker, Robert E. Schwartz, and Shuibing Chen

Figure S1

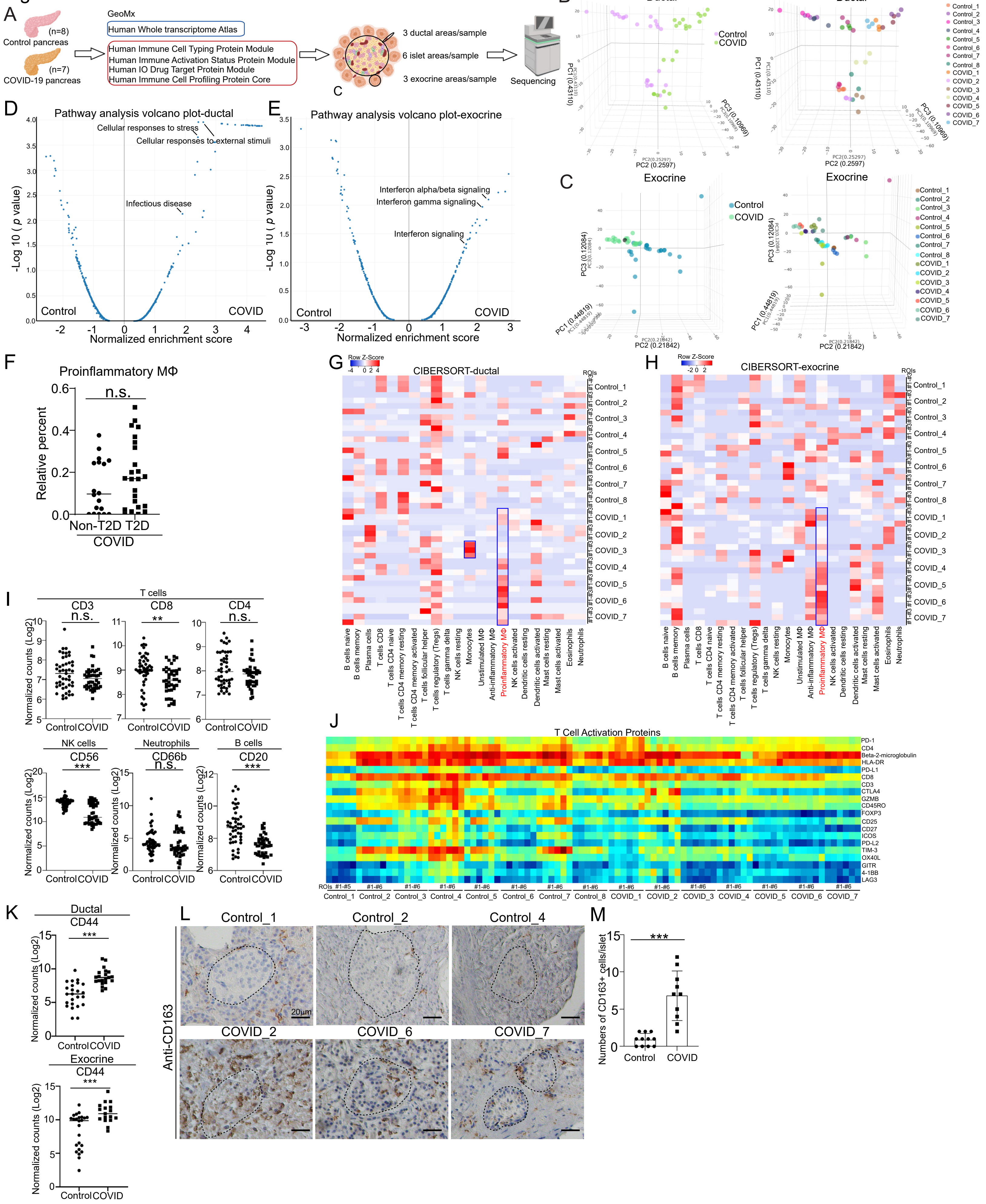

A
GeoMx
Human Whole transcriptome Atlas
Human Immune Cell Typing Protein Module
Human Immune Activation Status Protein Module
Human IO Drug Target Protein Module
Human Immune Cell Profiling Protein Core
(n=8) Control pancreas
(n=7) COVID-19 pancreas
3 ductal areas/sample
6 islet areas/sample
3 exocrine areas/sample
Sequencing
B Ductal
C Exocrine
D Pathway analysis volcano plot-ductal
Cellular responses to stress
Cellular responses to external stimuli
Infectious disease
E Pathway analysis volcano plot-exocrine
Interferon alpha/beta signaling
Interferon gamma signaling
Interferon signaling
Normalized enrichment score
F Proinflammatory MΦ
n.s.
Relative percent
Non-T2D T2D
COVID
G CIBERSORT-ductal
H CIBERSORT-exocrine
I T cells CD3 CD8 CD4
NK cells CD56
Neutrophils CD66b
B cells CD20
Normalized counts (Log2)
J T Cell Activation Proteins
K Ductal CD44
Exocrine CD44
L Control_1 Control_2 Control_4
COVID_2 COVID_6 COVID_7
Anti-CD163
20μm
M Numbers of CD163+ cells/islet
Control COVID

SUPPLEMENTAL INFORMATION

Figure S1. GeoMx spatial transcriptomics and protein analysis of pancreatic autopsy samples from control and COVID-19 subjects. Related to Figure 1.

(A) Schematic representation of GeoMx spatial transcriptomics and protein analysis.

(B and C) 3D PCA plots of GeoMx whole transcriptome sequencing data from ROIs of human ductal (B) and exocrine (C) areas of COVID-19 (N=7) and control (N=8) pancreatic autopsy samples.

(D and E) Volcano plot highlighting the pathways enriched in transcriptome sequencing data from ROIs of human ductal (D) and exocrine (E) areas of COVID-19 (N=7) and control (N=8) pancreatic autopsy samples.

(F) Relative percent of proinflammatory macrophages from the CIBERSORT analysis of immune cells (LM22) using the GeoMx whole transcriptome sequencing data of human islet areas in non-T2D or T2D COVID-19 pancreatic autopsy samples. Each dot represents one count in each ROI.

(G) Heatmap of the CIBERSORT analysis of immune cells (LM22) using the GeoMx whole transcriptome sequencing data from ROIs of human ductal areas of COVID-19 (N=7) and control (N=8) pancreatic autopsy samples.

(H) Heatmap of the CIBERSORT analysis of immune cells (LM22) using the GeoMx whole transcriptome sequencing data from ROIs of human exocrine areas of COVID-19 (N=7) and control (N=8) pancreatic autopsy samples.

(I) Normalized counts (Log2) of immune cell markers, including CD3, CD8 and CD4 for T cells, CD56 for NK cells, CD66b for neutrophils and CD20 for B cells, in ROIs of the islet areas of control (N=8) and COVID-19 (N=7) pancreatic autopsy samples. Each dot represents one count in each ROI.

(J) Heatmap of the proteins related to T cell activation examined by GeoMx protein assay from ROIs of human islet areas of COVID-19 (N=7) and control (N=8) pancreatic autopsy samples.

(K) Normalized counts (Log2) of CD44 in the ductal or exocrine areas of control (N=8) and COVID-19 (N=7) pancreatic autopsy samples. Each dot represents one count in each ROI.

(L and M) Immunohistochemistry staining (L) and quantification (M) of CD163 in COVID-19 (N=3) and control (N=3) pancreatic autopsy samples. Dotted lines encircled the regions of the islets. Scale bar=20 μm.

P values were calculated by unpaired two-tailed Student's t test. n.s., no significance, $^{**}P < 0.01$, $^{***}P < 0.001$.

Figure S2

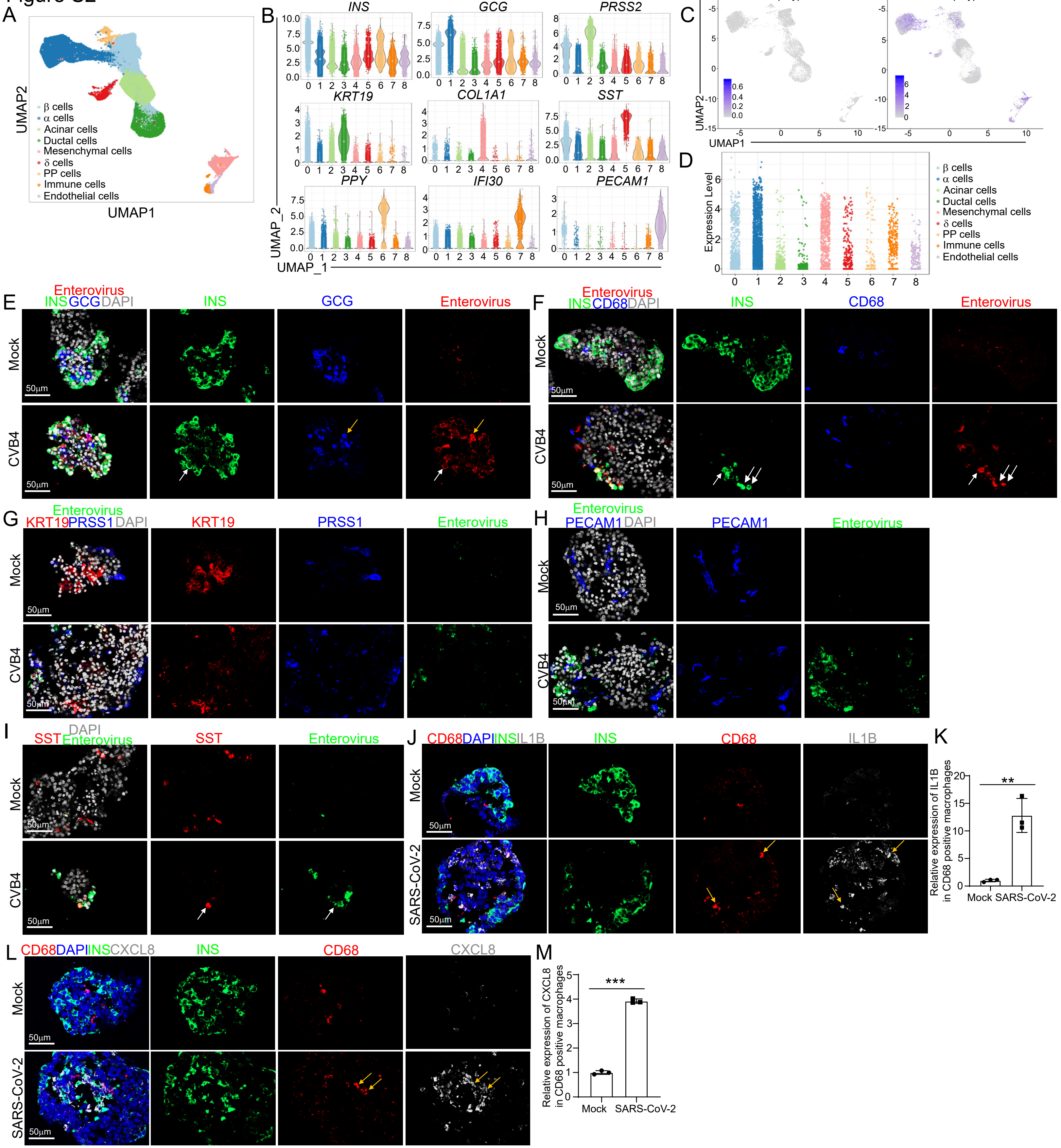

A
UMAP2
UMAP1
β cells
α cells
Acinar cells
Ductal cells
Mesenchymal cells
δ cells
PP cells
Immune cells
Endothelial cells
B
INS
GCG
PRSS2
KRT19
COL1A1
SST
PPY
IFI30
PECAM1
UMAP_2
UMAP_1
C
Mock-polyprotein
CVB4--polyprotein
UMAP2
UMAP1
D
Expression Level
E
Enterovirus
INS GCG DAPI
INS
GCG
Enterovirus
Mock
CVB4
50µm
F
Enterovirus
INS CD68 DAPI
INS
CD68
Enterovirus
G
Enterovirus
KRT19 PRSS1 DAPI
KRT19
PRSS1
Enterovirus
H
Enterovirus
PECAM1 DAPI
PECAM1
Enterovirus
I
SST DAPI Enterovirus
SST
Enterovirus
J
CD68 DAPI INS IL1B
INS
CD68
IL1B
SARS-CoV-2
K
Relative expression of IL1B in CD68 positive macrophages
**
Mock
SARS-CoV-2
L
CD68 DAPI INS CXCL8
INS
CD68
CXCL8
M
Relative expression of CXCL8 in CD68 positive macrophages
***
Mock
SARS-CoV-2

Figure S2. CVB4 infects human islets and induces activation of proinflammatory macrophages. Related to Figure 2.

(A) UMAP of human islets exposed to mock, SARS-CoV-2 (MOI=1) or CVB4 ($2x10^6$ PFU/ml) viruses.

(B) Violin plot of cell markers of each cell population, including *INS, GCG, PRSS2, KRT19*, *COL1A1*, *SST*, *PPY*, *IFI30*, and *PECAM1*.

(C) UMAP showed the expression of CVB4-*polyprotein* in human islets exposed to mock or CVB4 ($2x10^6$ PFU/ml) virus.

(D) Jitter plot showed the expression of CVB4-*polyprotein* in human islets exposed to mock or CVB4 ($2x10^6$ PFU/ml) virus.

(E) Confocal images of Enterovirus (CVB4) antigen expression in INS^+ β cells, GCG^+ α cells of human islets exposed to mock or CVB4 ($2x10^6$ PFU/ml). The white arrows highlight the co-localization of INS and Enterovirus antigen. The yellow arrows highlight the co-localization of GCG and Enterovirus antigen. Scale bar= 50 µm.

(F) Confocal images of Enterovirus (CVB4) antigen expression in INS^+ β cells, $CD68^+$ macrophages of human islets exposed to mock or CVB4 ($2x10^6$ PFU/ml). The white arrows highlight the co-localization of INS and Enterovirus antigen. Scale bar= 50 µm.

(G) Confocal images of Enterovirus (CVB4) antigen expression in $KRT19^+$ ductal cells, $PRSS1^+$ acinar cells of human islets exposed to mock or CVB4 ($2x10^6$ PFU/ml). Scale bar= 50 µm.

(H) Confocal images of Enterovirus (CVB4) antigen expression in $PECAM1^+$ endothelial cells of human islets exposed to mock or CVB4 ($2x10^6$ PFU/ml). Scale bar= 50 µm.

(I) Confocal images of Enterovirus (CVB4) antigen expression in SST^+ δ cells of human islets exposed to mock or CVB4 ($2x10^6$ PFU/ml). The white arrows highlight the co-localization of SST and Enterovirus antigen. Scale bar= 50 µm.

(J and K) Confocal images (J) and quantification (K) of IL1B expression in $CD68^+$ macrophages of human islets exposed to mock or SARS-CoV-2 (MOI=0.5). The yellow arrows highlight the co-localization of CD68 and IL1B. Scale bar= 50 µm.

(L and M) Confocal images (L) and quantification (M) of CXCL8 expression in $CD68^+$ macrophages of human islets exposed to mock or SARS-CoV-2 (MOI=0.5). The yellow arrows highlight the co-localization of CD68 and CXCL8. Scale bar= 50 µm.

n=3 independent biological replicates. Data was presented as mean ± STDEV. *P* values were calculated by unpaired two-tailed Student's t test. $^{**}P < 0.01$, $^{***}P < 0.001$.

Figure S3

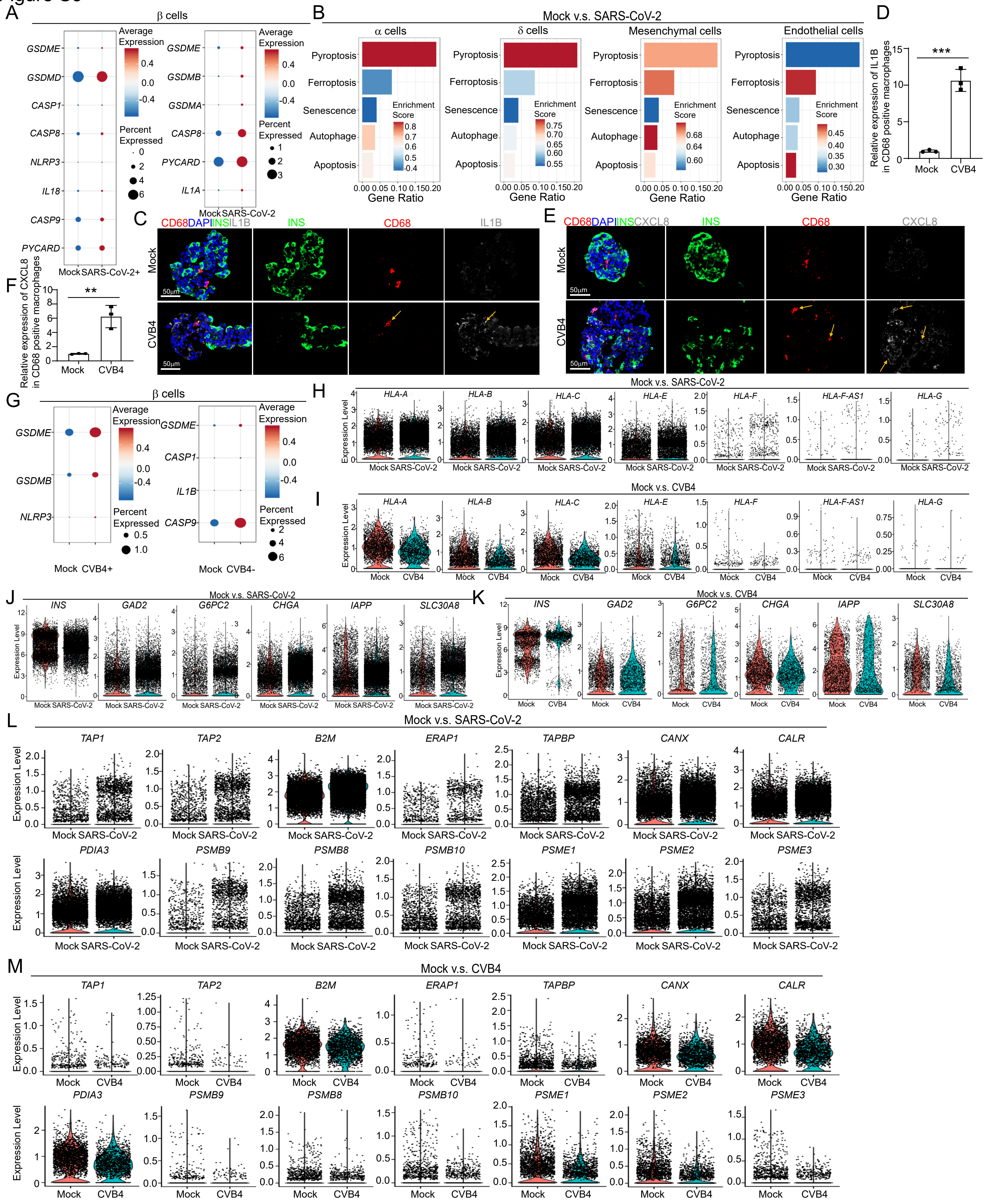

A
β cells
Average Expression
Percent Expressed
GSDME
GSDMD
CASP1
CASP8
NLRP3
IL18
CASP9
PYCARD
GSDMB
GSDMA
IL1A
Mock SARS-CoV-2+
Mock SARS-CoV-2
B
Mock v.s. SARS-CoV-2
α cells
δ cells
Mesenchymal cells
Endothelial cells
Pyroptosis
Ferroptosis
Senescence
Autophage
Apoptosis
Enrichment Score
Gene Ratio
C
CD68DAPIINSIL1B
INS
CD68
IL1B
Mock
CVB4
50μm
D
Relative expression of IL1B in CD68 positive macrophages
***
Mock
CVB4
E
CD68DAPIINSCXCL8
CXCL8
F
Relative expression of CXCL8 in CD68 positive macrophages
**
G
β cells
Mock CVB4+
Mock CVB4-
H
Mock v.s. SARS-CoV-2
Expression Level
HLA-A
HLA-B
HLA-C
HLA-E
HLA-F
HLA-F-AS1
HLA-G
I
Mock v.s. CVB4
J
Mock v.s. SARS-CoV-2
INS
GAD2
G6PC2
CHGA
IAPP
SLC30A8
K
Mock v.s. CVB4
L
Mock v.s. SARS-CoV-2
TAP1
TAP2
B2M
ERAP1
TAPBP
CANX
CALR
PDIA3
PSMB9
PSMB8
PSMB10
PSME1
PSME2
PSME3
M
Mock v.s. CVB4

Figure S3. Single cell RNA-seq analysis of human islets upon CVB4 or SARS-CoV-2 exposure. Related to Figure 2.

(A) Dot plot analysis of pyroptosis pathway associated genes in virus$^+$ β cell cluster and virus$^-$ β cell cluster of human islets upon mock or SARS-CoV-2 exposure (MOI=1).

(B) Pathway enrichment analysis of cell death pathways in α, δ, mesenchymal and endothelial cell clusters of human islets exposed to mock or SARS-CoV-2 (MOI=1).

(C and D) Confocal images (C) and quantification (D) of IL1B expression in CD68$^+$ macrophages of human islets exposed to mock or CVB4 ($2x10^6$ PFU/ml). The yellow arrows highlight the co-localization of CD68 and IL1B. Scale bar= 50 µm.

(E and F) Confocal images (E) and quantification (F) of CXCL8 expression in CD68$^+$ macrophages of human islets exposed to mock or CVB4 ($2x10^6$ PFU/ml). The yellow arrows highlight the co-localization of CD68 and CXCL8. Scale bar= 50 µm.

(G) Dot plot analysis of pyroptosis pathway associated genes in virus$^+$ β cell cluster and virus$^-$ β cell cluster of human islets upon mock or CVB4 ($2x10^6$ PFU/ml).

(H) Violin plot of the expression of *HLA* genes in the β cell cluster of human islets exposed to mock or SARS-CoV-2 (MOI=1).

(I) Violin plot of the expression of *HLA* genes in the β cell cluster of human islets exposed to mock or CVB4 ($2x10^6$ PFU/ml).

(J) Violin plot of the expression of autoantigen genes in the β cell cluster of human islets exposed to mock or SARS-CoV-2 (MOI=1).

(K) Violin plot of the expression of autoantigen genes in the β cell cluster of human islets exposed to mock or CVB4 ($2x10^6$ PFU/ml).

(L) Violin plot of the expression of antigen presentation associated genes in the β cell cluster of human islets exposed to mock or SARS-CoV-2 (MOI=1).

(M) Violin plot of the expression of antigen presentation genes in the β cell cluster of human islets exposed to mock or CVB4 ($2x10^6$ PFU/ml).

n=3 independent biological replicates. Data was presented as mean ± STDEV. *P* values were calculated by unpaired two-tailed Student's t test. $^{**}P < 0.01$, $^{***}P < 0.001$.

Figure S4

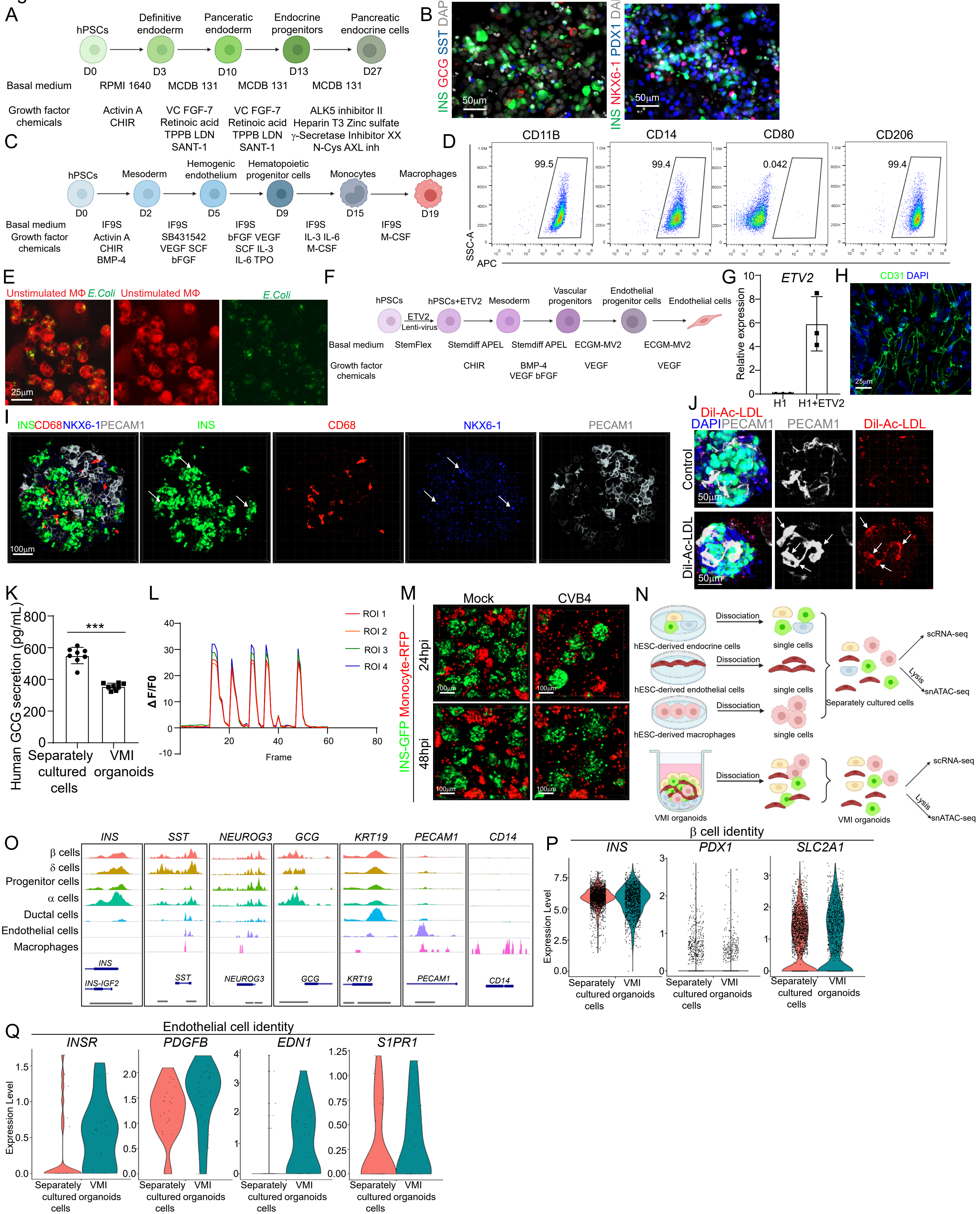

A
hPSCs
Definitive endoderm
Pancreatic endoderm
Endocrine progenitors
Pancreatic endocrine cells
D0
D3
D10
D13
D27
Basal medium
RPMI 1640
MCDB 131
MCDB 131
MCDB 131
Growth factor chemicals
Activin A CHIR
VC FGF-7 Retinoic acid TPPB LDN SANT-1
VC FGF-7 Retinoic acid TPPB LDN SANT-1
ALK5 inhibitor II Heparin T3 Zinc sulfate γ-Secretase Inhibitor XX N-Cys AXL inh
B
INS GCG SST DAPI
INS NKX6-1 PDX1 DAPI
50μm
C
hPSCs
Mesoderm
Hemogenic endothelium
Hematopoietic progenitor cells
Monocytes
Macrophages
D0
D2
D5
D9
D15
D19
Basal medium Growth factor chemicals
IF9S Activin A CHIR BMP-4
IF9S SB431542 VEGF SCF bFGF
IF9S bFGF VEGF SCF IL-3 IL-6 TPO
IF9S IL-3 IL-6 M-CSF
IF9S M-CSF
D
CD11B
CD14
CD80
CD206
99.5
99.4
0.042
99.4
SSC-A
APC
E
Unstimulated MΦ E.Coli
Unstimulated MΦ
E.Coli
25μm
F
hPSCs
hPSCs+ETV2
Mesoderm
Vascular progenitors
Endothelial progenitor cells
Endothelial cells
ETV2 Lenti-virus
Basal medium
StemFlex
Stemdiff APEL
Stemdiff APEL
ECGM-MV2
ECGM-MV2
Growth factor chemicals
CHIR
BMP-4 VEGF bFGF
VEGF
VEGF
G
ETV2
Relative expression
H1
H1+ETV2
H
CD31 DAPI
25μm
I
INS CD68 NKX6-1 PECAM1
INS
CD68
NKX6-1
PECAM1
100μm
J
Dil-Ac-LDL DAPI PECAM1
PECAM1
Dil-Ac-LDL
Control
Dil-Ac-LDL
50μm
K
Human GCG secretion (pg/mL)
***
Separately cultured cells
VMI organoids
L
ΔF/F0
Frame
ROI 1
ROI 2
ROI 3
ROI 4
M
Mock
CVB4
INS-GFP Monocyte-RFP
24hpi
48hpi
100μm
N
hESC-derived endocrine cells
hESC-derived endothelial cells
hESC-derived macrophages
Dissociation
single cells
Separately cultured cells
scRNA-seq
Lysis
snATAC-seq
VMI organoids
O
INS
SST
NEUROG3
GCG
KRT19
PECAM1
CD14
β cells
δ cells
Progenitor cells
α cells
Ductal cells
Endothelial cells
Macrophages
INS-IGF2
P
β cell identity
INS
PDX1
SLC2A1
Expression Level
Separately cultured cells
VMI organoids
Q
Endothelial cell identity
INSR
PDGFB
EDN1
S1PR1
Expression Level
Separately cultured cells
VMI organoids

Figure S4. Construction and characterization of hPSC-derived VMI organoids. Related to Figure 3 and Figure 4.

(A) Schematic illustration of directed differentiation of hPSCs to pancreatic endocrine cells. At day 16, we detected INS-GFP^{+} cells. Then, early stage 6 cells (day 16-day19) were co-cultured with macrophages and endothelial cells or culture separately for 7-14 days.

(B) Confocal images of hPSC-derived pancreatic endocrine cells stained with antibodies against INS, GCG, NKX6-1, PDX1 and SST. Scale bar= 50 µm.

(C) Schematic illustration of directed differentiation of hESCs to macrophages.

(D) Flow cytometry analysis of hPSC-derived unstimulated macrophages stained with antibodies against CD11B, CD14, CD80 and CD206.

(E) Confocal images of hPSC-derived macrophages engulfing GFP labeled *E.Coli*. Macrophages: RFP; *E.Coli: Green*. Scale bar= 25 µm.

(F) Schematic illustration of directed differentiation of hPSCs to endothelial cells.

(G) qRT-PCR analysis to examine the expression level of *ETV2* in H1 hPSCs following forced expression of ETV2 or control. Data was normalized to β-actin.

(H) Confocal images of hPSC-derived endothelial cells stained with antibodies against PECAM1 (CD31) and DAPI. Scale bar= 25 µm.

(I) Composite Z-stack confocal images of VMI organoids at day 14 after reaggregation stained with antibodies against INS, CD68, NKX6-1 and PECAM1 (CD31). The white arrows highlight the co-localization of INS and NKX6-1. Scale bar= 100 µm.

(J) Confocal images of VMI organoids stained with antibodies against PECAM1 (CD31) and Dil-Ac-LDL. Scale bar= 50 µm.

(K) ELISA assay showed the secretion of GCG in VMI organoids and separately cultured endocrine cells.

(L) Quantification of calcium signaling in VMI organoids upon high glucose stimulation. High glucose: 20 mM D-glucose. Each frame was captured every 500ms.

(M) Live cell imaging of VI organoids and monocytes exposed to mock or CVB4 ($2x10^6$ PFU/ml) at 24 hpi and 48 hpi. Scale bar= 100 µm.

(N) Schematic illustration of the sample preparation for scRNA-seq and snATAC-seq.

(O) Chromatin accessibility signals of cell markers for each cluster as analyzed using snATAC-seq.

(**P)** Violin plot analysis of β cell associated genes in β cell cluster of VMI organoids at day 7 after reaggregation and separately cultured cells as analyzed by scRNA-seq.

(**Q)** Violin plot analysis of endothelial cell associated genes in endothelial cell cluster of VMI organoids at day 7 after reaggregation and separately cultured cells as analyzed by scRNA-seq.

n=3 independent biological replicates. Data was presented as mean ± STDEV. *P* values were calculated by unpaired two-tailed Student's t test. ****P* < 0.001.

Figure S5

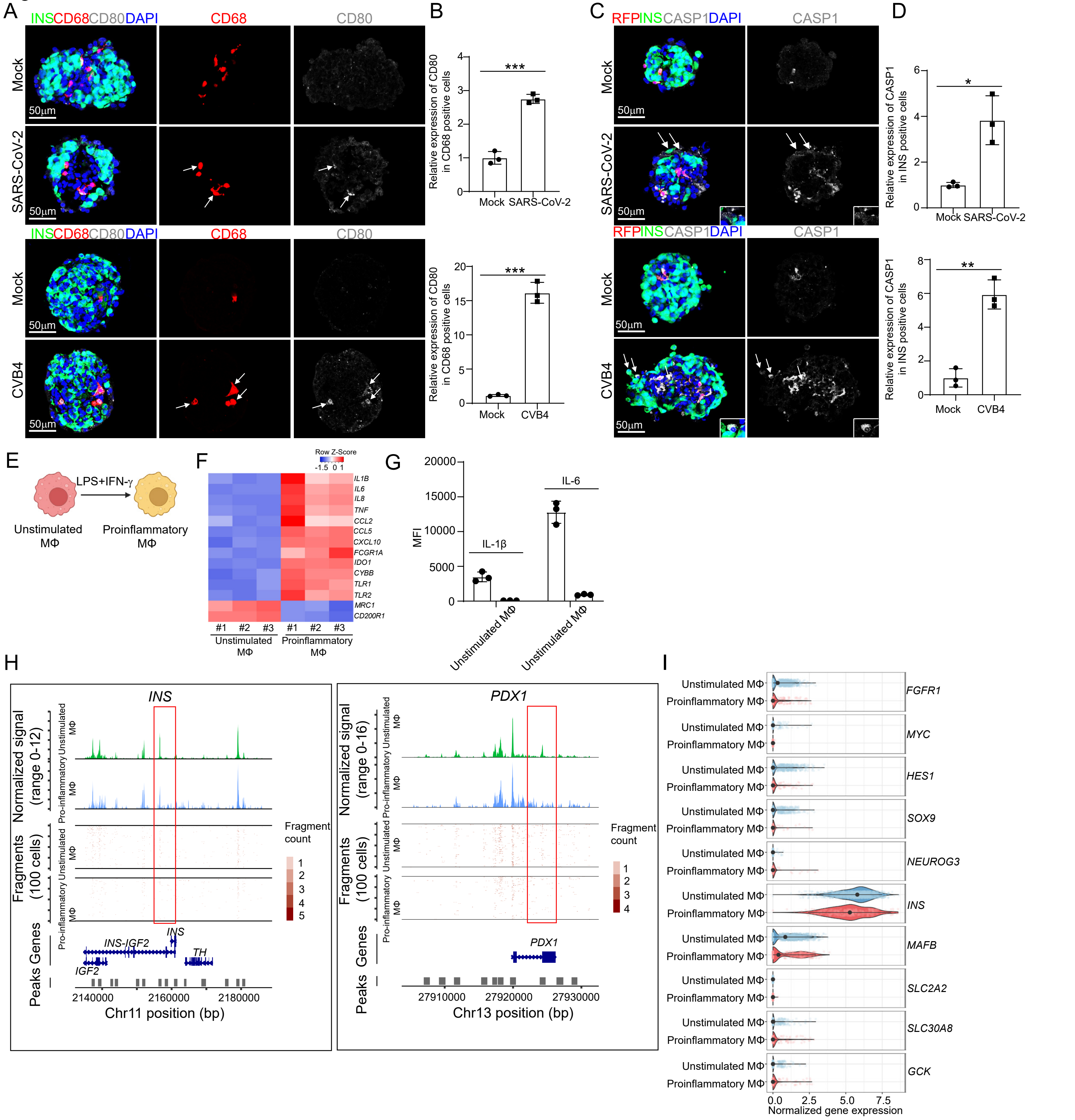

A
INSCD68CD80DAPI
CD68
CD80
Mock
SARS-CoV-2
CVB4
50µm
B
Relative expression of CD80 in CD68 positive cells
***
Mock
SARS-CoV-2
CVB4
C
RFPINSCASP1DAPI
CASP1
D
Relative expression of CASP1 in INS positive cells
*
**
E
LPS+IFN-γ
Unstimulated MΦ
Proinflammatory MΦ
F
Row Z-Score
IL1B
IL6
IL8
TNF
CCL2
CCL5
CXCL10
FCGR1A
IDO1
CYBB
TLR1
TLR2
MRC1
CD200R1
#1 #2 #3
Unstimulated MΦ
Proinflammatory MΦ
G
MFI
IL-1β
IL-6
Unstimulated MΦ
H
INS
PDX1
Normalized signal (range 0-12)
Normalized signal (range 0-16)
Unstimulated MΦ
Pro-inflammatory MΦ
Fragments (100 cells)
Fragment count
Genes
Peaks
INS-IGF2
IGF2
TH
Chr11 position (bp)
Chr13 position (bp)
I
FGFR1
MYC
HES1
SOX9
NEUROG3
INS
MAFB
SLC2A2
SLC30A8
GCK
Normalized gene expression

Figure S5. Activation of proinflammatory macrophages and β cell pyroptosis were detected in hPSC-derived VMI organoids exposed to viruses. Related to Figure 5.

(A and B) Confocal images (A) and quantification (B) of hPSC-derived VMI organoids exposed to viruses or mock conditions stained with antibodies against INS, CD68 and CD80 (SARS-CoV-2: MOI=0.5; CVB4: $2x10^6$ PFU/ml). Scale bar= 50 µm. The white arrows highlight the $CD68^+CD80^+$ cells.

(C and D) Confocal images (C) and quantification (D) of hPSC-derived VMI organoids exposed to viruses or mock conditions stained with antibodies against INS and CASP1 (SARS-CoV-2: MOI=0.5; CVB4: $2x10^6$ PFU/ml). Scale bar= 50 µm. The white arrows highlight the INS^+CASP1^+ cells.

(E) Schematic illustration of the stimulation of macrophages to proinflammatory macrophages.

(F) Heatmap showing the expression of macrophage associated genes in hPSC-derived macrophages with or without 2 days treatment with 100 ng/ml LPS and 20 ng/ml IFN-γ.

(**G)** The secretion of cytokines, including IL-1β and IL-6 in the supernatant of hPSC-derived macrophages with or without 2 days treatment with 100 ng/ml LPS and 20 ng/ml IFN-γ.

(H) Chromatin accessibility signals of the β cluster of VMI organoids at day 7 after reaggregation containing unstimulated macrophages or proinflammatory macrophages as analyzed using snATAC-seq. The normalized signal shows the averaged frequency of sequenced DNA fragments within a genomic region. The fragment shows the frequency of sequenced fragments within a genomic region for individual cells.

(**I)** Jitter plot analysis of β cell dedifferentiation associated genes in β cell cluster of VMI organoids with unstimulated macrophages or proinflammatory macrophages at day 7 after reaggregation as analyzed by scRNA-seq.

n=3 independent biological replicates. Data was presented as mean ± STDEV. *P* values were calculated by unpaired two-tailed Student's t test. **P* < 0.05, ***P* < 0.01, ****P* < 0.001.

Figure S6

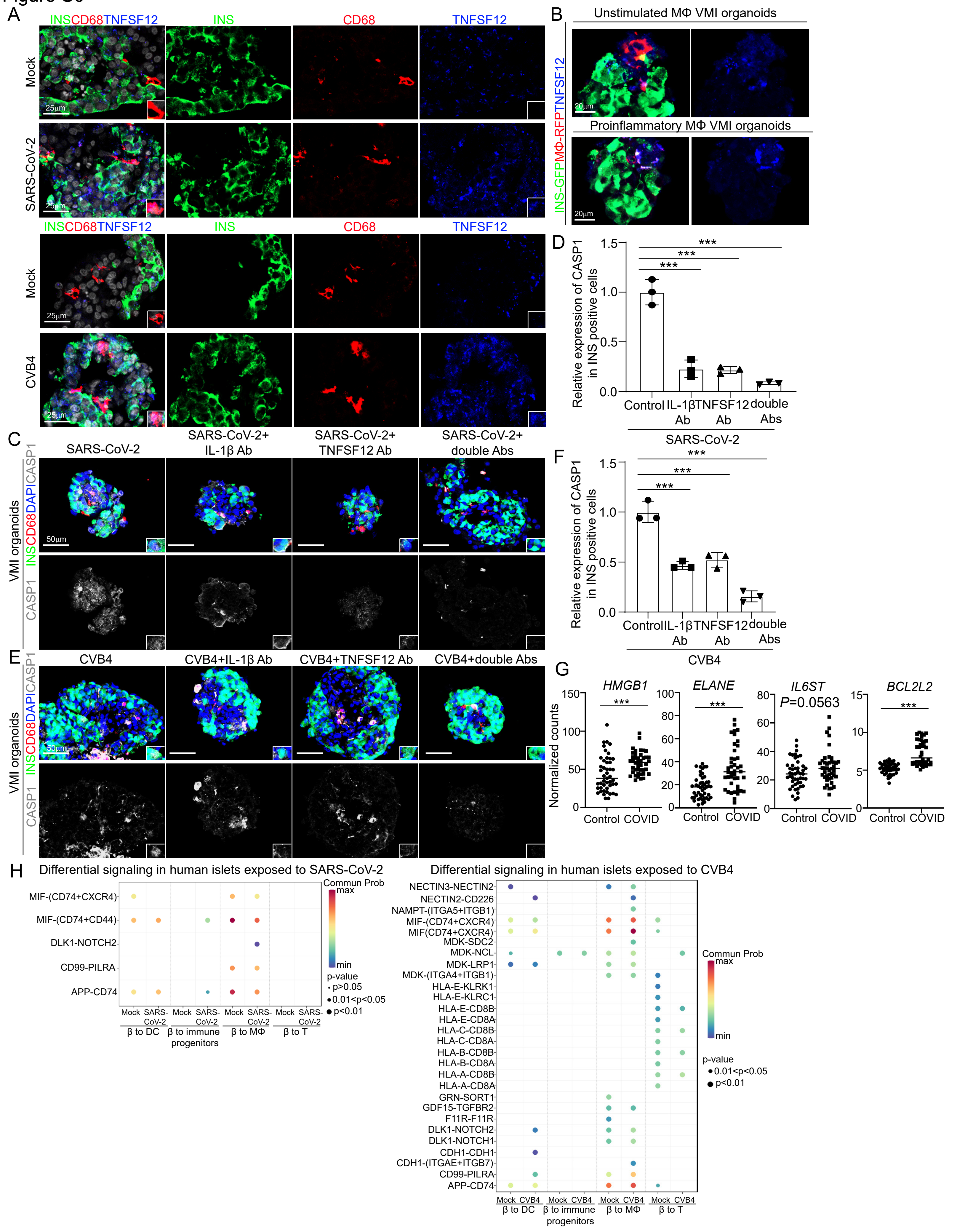
A
INSCD68TNFSF12
INS
CD68
TNFSF12
Mock
SARS-CoV-2
CVB4
25μm
B
Unstimulated MΦ VMI organoids
Proinflammatory MΦ VMI organoids
INS-GFPMΦ-RFPTNFSF12
20μm
C
SARS-CoV-2
SARS-CoV-2+ IL-1β Ab
SARS-CoV-2+ TNFSF12 Ab
SARS-CoV-2+ double Abs
VMI organoids
INSCD68DAPICASP1
CASP1
50μm
D
Relative expression of CASP1 in INS positive cells
Control
IL-1β Ab
TNFSF12 Ab
double Abs
SARS-CoV-2
***
E
CVB4
CVB4+IL-1β Ab
CVB4+TNFSF12 Ab
CVB4+double Abs
F
CVB4
G
HMGB1
ELANE
IL6ST
P=0.0563
BCL2L2
Normalized counts
Control
COVID
H
Differential signaling in human islets exposed to SARS-CoV-2
MIF-(CD74+CXCR4)
MIF-(CD74+CD44)
DLK1-NOTCH2
CD99-PILRA
APP-CD74
Commun Prob
max
min
p-value
p>0.05
0.01<p<0.05
p<0.01
Mock
SARS-CoV-2
β to DC
β to immune progenitors
β to MΦ
β to T
Differential signaling in human islets exposed to CVB4
NECTIN3-NECTIN2
NECTIN2-CD226
NAMPT-(ITGA5+ITGB1)
MIF-(CD74+CXCR4)
MIF(CD74+CXCR4)
MDK-SDC2
MDK-NCL
MDK-LRP1
MDK-(ITGA4+ITGB1)
HLA-E-KLRK1
HLA-E-KLRC1
HLA-E-CD8B
HLA-E-CD8A
HLA-C-CD8B
HLA-C-CD8A
HLA-B-CD8B
HLA-B-CD8A
HLA-A-CD8B
HLA-A-CD8A
GRN-SORT1
GDF15-TGFBR2
F11R-F11R
DLK1-NOTCH2
DLK1-NOTCH1
CDH1-CDH1
CDH1-(ITGAE+ITGB7)
CD99-PILRA
APP-CD74
Mock
CVB4

Figure S6. TNFSF12 expression in human islets exposed to CVB4 or SARS-CoV-2, and VMI organoids with proinflammatory macrophages. Related to Figure 6.

(A) Confocal images of TNFSF12 in human islets exposed to mock, CVB4 ($2x10^6$ PFU/ml) or SARS-CoV-2 (MOI=0.5). The inserts show a high magnification of cells. Scale bar= 25 µm.

(B) Confocal images of TNFSF12 in VMI organoids containing unstimulated or pro-inflammatory macrophages at day 7 after reaggregation. Scale bar= 20 µm.

(C and D) Confocal images (C) and quantification (D) of the CASP1 expression in INS^+ cells of VMI organoids exposed to SARS-CoV-2 (MOI=0.5) and treated with control, 10 µg/ml TNFSF12 blocking antibody, 5 µg/ml IL-1β blocking antibody or 10 µg/ml TNFSF12 + 5 µg/ml IL-1β blocking antibodies. The inserts show a high magnification of cells. Scale bar= 50 µm.

(E and F) Confocal images (E) and quantification (F) of the CASP1 expression in INS^+ cells of VMI organoids exposed to CVB4 ($2x10^6$ PFU/ml) and treated with control, 10 µg/ml TNFSF12 blocking antibody, 5 µg/ml IL-1β blocking antibody or 10 µg/ml TNFSF12 + 5 µg/ml IL-1β blocking antibodies. The inserts show a high magnification of cells. Scale bar= 50 µm.

(G) Normalized counts of pyroptosis associated genes expression in control or COVID-19 samples examined by GeoMx transcriptomic assays. Each dot represents one count in each ROI.

(H) Cell chat analysis showed the interactions from β cells to immune cell subpopulations, including DC cells, immune progenitors, T cells and macrophages in human islets exposed to SARS-CoV-2 (MOI=1) or CVB4 ($2x10^6$ PFU/ml).

n=3 independent biological replicates. Data was presented as mean ± STDEV. *P* values were calculated by one-way ANOVA with a common control. ****P* < 0.001.

Table S1. Patient information. Related to Figures 1, 2, 6 and Figures S1, S2, S3, S6.

Sample name	Age	Sex	Pre-existing diabetes	Tissues	Obesity (BMI)	Family Hx	Laboratory data (WBC)	The onset and severity of the disease	Time to death from onset	Comorbidities	Glucose	Transport, and processing of these samples	Cause of death
Control_1	58	F	N	pancreas	24.2	Yes, Brother	7.6 - 12.2	N/A	N/A	N/A	60-325	Within 1 day	Anoxia
Control_2	64	M	N	pancreas	25.12	N/A	N/A	N/A	N/A	N/A	96-105	Within 1 day	Anoxia
Control_3	64	F	N	pancreas	20.92	N/A	N/A	N/A	N/A	N/A	131-242	Within 1 day	CVA
Control_4	66	F	N	pancreas	32.98	N/A	N/A	N/A	N/A	N/A	154-354	Within 1 day	CVA
Control_5	61	F	N	pancreas	29.85	N/A	N/A	N/A	N/A	N/A	195-724	Within 1 day	Anoxia
Control_6	60	F	T2D	pancreas	29.7	N/A	14.2 - 20.4	N/A	N/A	N/A	58-482	Within 1 day	Anoxia
Control_7	59	M	N	pancreas	21.32	N/A	N/A	N/A	N/A	N/A	106-344	Within 1 day	Trauma
Control_8	62	F	T2D	pancreas	37.01	N/A	N/A	N/A	N/A	N/A	76-407	Within 1 day	CVA
Control_9	58	F	N	islets	31.07	No	8.7 - 16.4	N/A	N/A	N/A	131-212	Within 1 day	CVA
Control_10	27	M	N	islets	25.3	No	4.5 - 9.4	N/A	N/A	N/A	128-520	Within 1 day	CAR
Control_11	39	M	N	islets	31.07	No	8 - 14.7	N/A	N/A	N/A	130-346	Within 1 day	Anoxia
Control_12	57	M	N	islets	24.5	Yes, Mom & Dad	6.7 - 8.9	N/A	N/A	N/A	104-241	Within 1 day	CVA
COVID_1	58	M	T2D	pancreas	29.19	No	29.1	Hospitalized not intubat	21 days	HTN	75-171	Within 1 day	COVID Pneumonia

								ed, Required O2					
COVID_2	64	M	T2D	pancreas	19.77	No	17.4	Hospitalized not intubated, Required O2	1 day	HTN	103-296	Within 2 days	Respiratory failure and pneumonia
COVID_3	75	F	N	pancreas	23.62	No	35.07	Hospitalized not intubated, Required O2	12 days	Multiple Myeloma in good control, H/O DVT and PE on AC	43-179	Within 1 day	COVID Pneumonia
COVID_4	81	F	N	pancreas	20.27	No	22.7	Hospitalized not intubated, Required O2	9 days	CAD, Gout, HTN, Hyperlipidemia, Dementia	133-221	Within 1 day	COVID Pneumonia
COVID_5	53	M	T2D	pancreas	30.63	No	21.3	Hospitalized not intubated, Required O2	47 days	HTN, CVA	81-292	Within 2 days	Non COVID Pneumonia
COVID_6	87	M	T2D	pancreas	21.03	No	6.8	Hospitalized not intubated, Required O2	5 days	HTN, T2DM, Dementia	50-307	Within 1 day	COVID Pneumonia
COVID_7	71	F	N	pancreas	22.76	Yes, Brother	19.3	Hospitalized, Intubated	29 days	HTN, AML in remission	118-254	Within 1 day	COVID Pneumonia

						& Mom							
COVID_8	55	M	N	pancreas	Not recorded	No	17.46	Hospitalized not intubated, Required O2	14 days	HTN, CAD, Hyperlipidemia	110-263	Within 2 days	Respiratory failure and pneumonia

Table S2. Antibodies used for immunocytochemistry, intracellular flow cytometry analysis. Related to STAR Methods.

Usage	Antibody	Clone #	Host	Catalog #	Vendor	Dilution
Immunostaining	Polyclonal Guinea Pig Anti-Insulin	Polyclonal	Guinea Pig	#A0564	Dako	1:500
Immunostaining	Glucagon Rabbit Ab	Polyclonal	Rabbit	#2760	Cell Signaling	1:1000
Immunostaining	Polyclonal Rabbit Anti-Somatostatin	Polyclonal	Rabbit	#A0566	Dako	1:1000
Immunostaining	Human CD31/PECAM-1 Antibody	Polyclonal	Sheep	#AF806	R&D Systems	1:1000
Immunostaining	Purified anti-human CD68 Antibody	Monoclonal	Mouse	# 333802	Biolegend	1: 100
Immunostaining	Cleaved Caspase-1 (Asp297)	Monoclonal	Rabbit	# 4199	Cell Signaling	1: 200
Immunostaining	hPDX-1 Affinity purified goat igG	Polyclonal	Goat	# AF2419	R&D Systems	1:500
Immunostaining	Nkx6.1 (D8O4R) Rabbit mAb	Monoclonal	Rabbit	#54551	Cell Signaling	1: 500
Flow Cytometry	APC anti-mouse/human CD11b Antibody	Monoclonal	Rat	#101212	Biolegend	1: 50
Flow Cytometry	APC anti-human CD206 (MMR) Antibody	Monoclonal	Mouse	#321109	Biolegend	1:50
Flow Cytometry	APC anti-human CD14	Monoclonal	Mouse	#301808	Biolegend	1:100
GeoMx	Insulin Monoclonal Antibody (ICBTACLS), Alexa Fluor™ 488	Monoclonal	Mouse	#53-9769-82	Thermo Fisher Scientific	1:200

GeoMx	Cytokeratin, pan Antibody (AE-1/AE-3) [DyLight 594]	Monoclonal	Mouse	# NBP2-33200DL594	Novus Biological	1:200
Immunostaining	caspase-1 Antibody (14F468)	Monoclonal	Mouse	#sc-56036	Santa Cruz	1:200
Immunostaining	Enterovirus (Concentrate)	Monoclonal	Mouse	#M7064	Dako	1:500
Immunostaining	CD163 (D6U1J) Rabbit mAb	Monoclonal	Rabbit	#93498	Cell Signaling	1:200
Immunostaining	Anti-PRSS1 antibody produced in rabbit	Polyclonal	Rabbit	#HPA063471	Sigma Aldrich	1:500
GeoMx	Purified anti-Cytokeratin 19	Monoclonal	Mouse	#628502	Biolegend	1:1000
Immunohistochemistry	Human B7-1/CD80 MAb (Clone 37711)	Monoclonal	Mouse	# MAB140-100	RnD	1:500
Immunostaining	Alexa Fluor 488 AffiniPure Donkey Anti-Guinea Pig IgG (H+L)	Polyclonal	Donkey	#706-545-148	Jackson Immunoresearch Labs	1:500
Immunostaining	Donkey anti-Mouse IgG (H+L) Highly Cross-Adsorbed Secondary Antibody, Alexa Fluor 594	Polyclonal	Donkey	#A-21203	Thermo Fisher Scientific	1:500
Immunostaining	Donkey anti-Rabbit IgG (H+L) Secondary Antibody, Alexa Fluor 594 conjugate	Polyclonal	Donkey	#A-21207	Thermo Fisher Scientific	1:500
Immunostaining	Donkey anti-Rabbit IgG (H+L) Secondary Antibody,	Polyclonal	Donkey	#A-31573	Thermo Fisher Scientific	1:500

	Alexa Fluor 647 conjugate					
Immunostaining	Donkey anti-Mouse IgG (H+L) Secondary Antibody, Alexa Fluor 647	Polyclonal	Donkey	#A-31571	Thermo Fisher Scientific	1:500
Immunostaining	Donkey anti-Goat IgG (H+L) Cross-Adsorbed Secondary Antibody, Alexa Fluor 647	Polyclonal	Donkey	#A-21447	Thermo Fisher Scientific	1:500
Immunostaining	Donkey anti-Sheep IgG (H+L) Cross-Adsorbed Secondary Antibody, Alexa Fluor 647	Polyclonal	Donkey	#A-21448	Thermo Fisher Scientific	1:500
Immunostaining	Donkey anti-Mouse IgG (H+L) Highly Cross-Adsorbed Secondary Antibody, Alexa Fluor Plus 405	Polyclonal	Donkey	#A48257	Thermo Fisher Scientific	1:500

Table S3. Primers used for qRT-PCR. Related to STAR Methods.

**Primer name**	**Sequence**
ACTB-Forward	*CGTCACCAACTGGGACGACA*
ACTB-Reverse	*CTTCTCGCGGTTGGCCTTGG*
ETV2-F	*GAAGGAGCCAAATTAGGCTTCT*
ETV2-R	*GAGCTTGTACCTTTCCAGCAT*
