# Supplementary material for: Human vascularized macrophage-islet organoids to model immune-mediated pancreatic β cell pyroptosis upon viral infection
Source: Cell Stem Cell. 2024 Nov 7;31(11):1612–1629.e8. doi: 10.1016/j.stem.2024.08.007 (PMC11546835; doi:10.1016/j.stem.2024.08.007)
Supplement: Document S2. Article plus supplemental information [file mmc11.pdf]

Cell Stem Cell

Article

Human vascularized macrophage-islet organoids to model immune-mediated pancreatic β cell pyroptosis upon viral infection

Graphical abstract

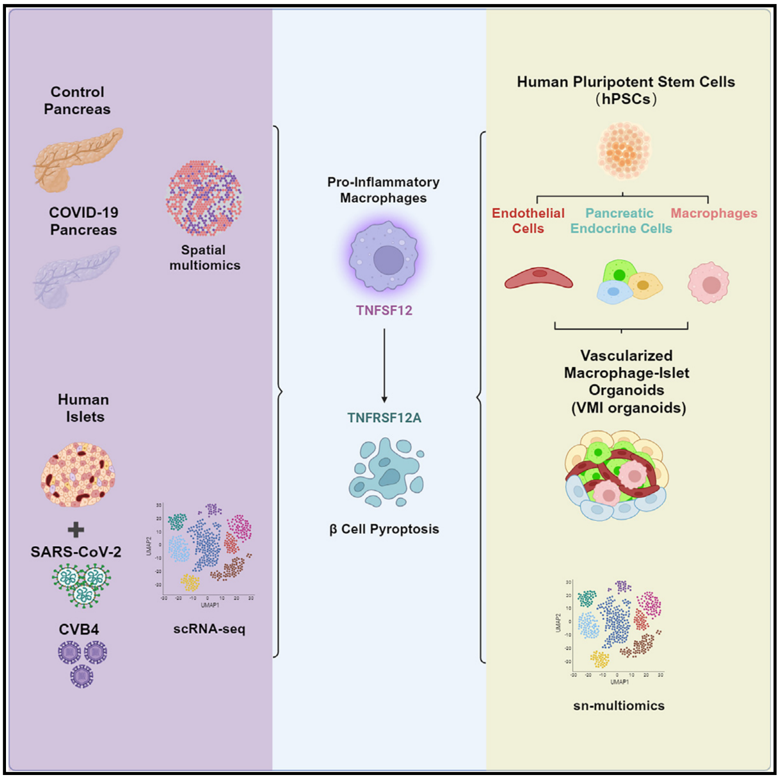

Authors

Liuliu Yang, Yuling Han, Tuo Zhang, ..., Stephen C.J. Parker, Robert E. Schwartz, Shuibing Chen

Correspondence

yangliuliu@ihcams.ac.cn (L.Y.), res2025@med.cornell.edu (R.E.S.), shc2034@med.cornell.edu (S.C.)

In brief

Yang and colleagues found proinflammatory macrophages enriched in COVID-19 pancreatic samples. Exposure to SARS-CoV-2 or coxsackievirus B4 induced proinflammatory macrophage activation and β cell pyroptosis. Human pluripotent stem cell-derived vascularized macrophage-islet organoids demonstrated that proinflammatory macrophages induced β cell pyroptosis through TNFSF12 signal.

Highlights

- Proinflammatory macrophages are enriched in COVID-19 pancreatic autopsy samples
- Virus exposure causes proinflammatory macrophage activation and β cell pyroptosis
- Construction of hPSC-derived vascularized macrophage-islet organoids
- Proinflammatory macrophages induce β cell pyroptosis through TNFSF12 signal

Yang et al., 2024, Cell Stem Cell *31*, 1612–1629
November 7, 2024 © 2024 The Author(s). Published by Elsevier Inc.
https://doi.org/10.1016/j.stem.2024.08.007

CellPress

CellPress
OPEN ACCESS

Cell Stem Cell

Article

Human vascularized macrophage-islet organoids to model immune-mediated pancreatic β cell pyroptosis upon viral infection

Liuliu Yang,[1,2,3,4,16,*] Yuling Han,[1,2,5,6,7,16] Tuo Zhang,[8,16] Xue Dong,[1] Jian Ge,[9] Aadita Roy,[1] Jiajun Zhu,[1,2] Tiankun Lu,[1,2] J. Jeya Vandana,[1,2] Neranjan de Silva,[1,2] Catherine C. Robertson,[10] Jenny Z. Xiang,[8] Chendong Pan,[8] Yanjie Sun,[8] Jianwen Que,[9] Todd Evans,[1,2] Chengyang Liu,[11] Wei Wang,[11] Ali Naji,[11] Stephen C.J. Parker,[10,12,13] Robert E. Schwartz,[14,15,*] and Shuibing Chen[1,2,17,*]

[1]Department of Surgery, Weill Cornell Medicine, 1300 York Avenue, New York, NY 10065, USA
[2]Center for Genomic Health, Weill Cornell Medicine, 1300 York Avenue, New York, NY 10065, USA
[3]State Key Laboratory of Experimental Hematology, National Clinical Research Center for Blood Disease, Haihe Laboratory of Cell Ecosystem, Institute of Hematology & Blood Diseases Hospital, Chinese Academy of Medical Sciences & Peking Union Medical College, Tianjin 300020, China
[4]Tianjin Institute of Health Science, Tianjin 301600, China
[5]Key Laboratory of Organ Regeneration and Reconstruction, State Key Laboratory of Stem Cell and Reproductive Biology, Institute of Zoology, Chinese Academy of Sciences, Beijing 100101, China
[6]Institute for Stem Cell and Regeneration, Chinese Academy of Sciences, Beijing 100101, China
[7]Beijing Institute for Stem Cell and Regenerative Medicine, Beijing 100101, China
[8]Genomic Resource Core Facility, Weill Cornell Medicine, New York, NY 10065, USA
[9]Columbia Center for Human Development, Department of Medicine, Columbia University Irving Medical Center, New York, NY 10032, USA
[10]Department of Computational Medicine and Bioinformatics, University of Michigan, Ann Arbor, MI, USA
[11]Department of Surgery, University of Pennsylvania School of Medicine, Philadelphia, PA 19104, USA
[12]Department of Human Genetics, University of Michigan, Ann Arbor, MI, USA
[13]Department of Biostatistics, University of Michigan, Ann Arbor, MI, USA
[14]Division of Gastroenterology and Hepatology, Department of Medicine, Weill Cornell Medicine, 1300 York Avenue, New York, NY 10065, USA
[15]Department of Physiology, Biophysics and Systems Biology, Weill Cornell Medicine, 1300 York Avenue, New York, NY 10065, USA
[16]These authors contributed equally
[17]Lead contact
*Correspondence: yangliuliu@ihcams.ac.cn (L.Y.), res2025@med.cornell.edu (R.E.S.), shc2034@med.cornell.edu (S.C.)
https://doi.org/10.1016/j.stem.2024.08.007

SUMMARY

There is a paucity of human models to study immune-mediated host damage. Here, we utilized the GeoMx spatial multi-omics platform to analyze immune cell changes in COVID-19 pancreatic autopsy samples, revealing an accumulation of proinflammatory macrophages. Single-cell RNA sequencing (scRNA-seq) analysis of human islets exposed to severe acute respiratory syndrome coronavirus 2 (SARS-CoV-2) or coxsackievirus B4 (CVB4) viruses identified activation of proinflammatory macrophages and β cell pyroptosis. To distinguish viral versus proinflammatory-macrophage-mediated β cell pyroptosis, we developed human pluripotent stem cell (hPSC)-derived vascularized macrophage-islet (VMI) organoids. VMI organoids exhibited enhanced marker expression and function in both β cells and endothelial cells compared with separately cultured cells. Notably, proinflammatory macrophages within VMI organoids induced β cell pyroptosis. Mechanistic investigations highlighted TNFSF12-TNFRSF12A involvement in proinflammatory-macrophage-mediated β cell pyroptosis. This study established hPSC-derived VMI organoids as a valuable tool for studying immune-cell-mediated host damage and uncovered the mechanism of β cell damage during viral exposure.

INTRODUCTION

A strong connection between coronavirus disease 19 (COVID-19) and diabetes is now recognized. Since the beginning of the pandemic, there have been reports of new-onset diabetes[1–5] and exacerbated complications in patients with pre-existing diabetes. Moreover, a rise in type 1 diabetes (T1D) incidence has been observed.[4,6] A study from the Centers for Disease Control and Prevention reported that persons aged <18 years with COVID-19 were more inclined to receive a new diabetes

 © 2024 The Author(s). Published by Elsevier Inc.
This is an open access article under the CC BY license (http://creativecommons.org/licenses/by/4.0/).

CellPress
OPEN ACCESS

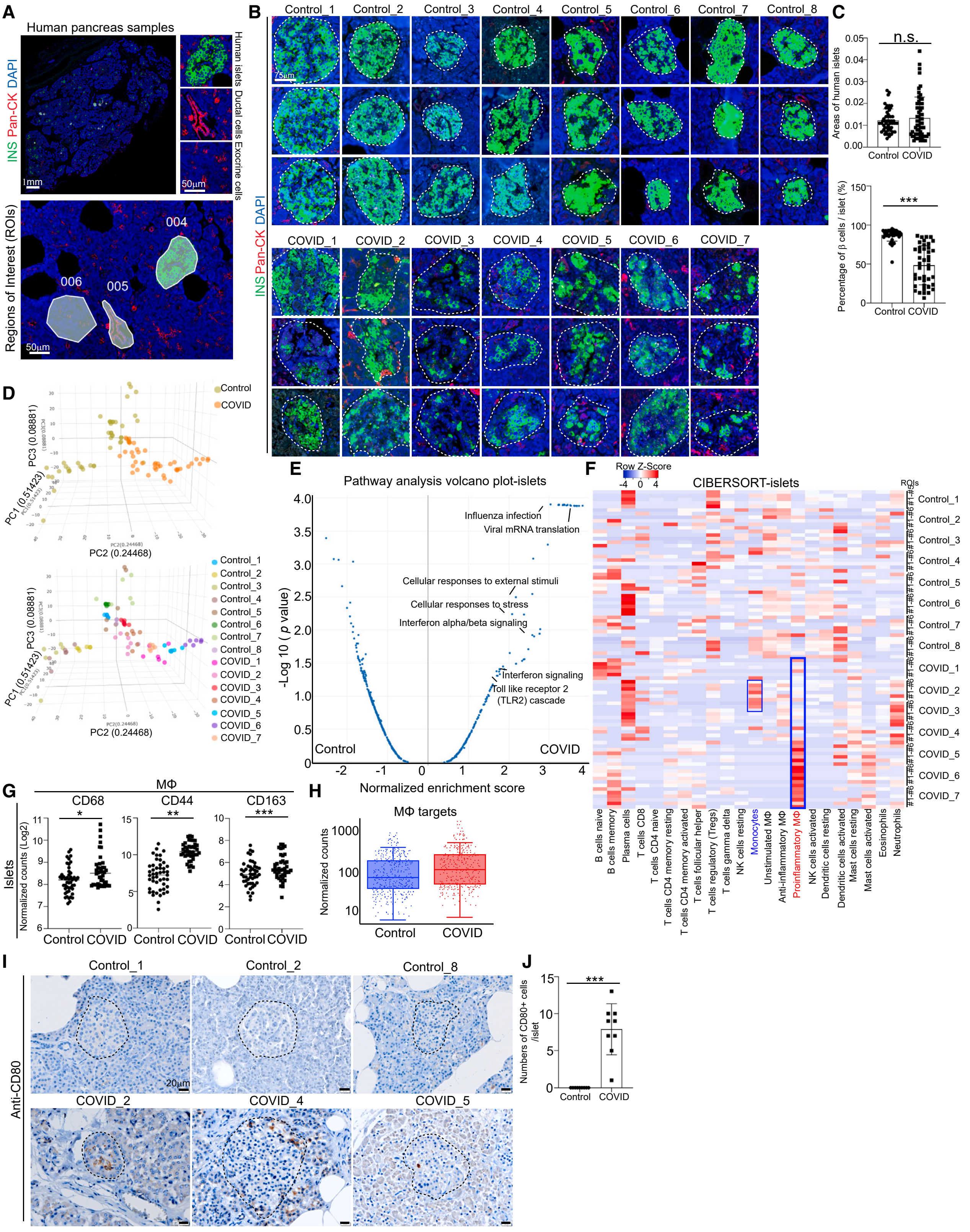

Figure 1. Macrophage accumulation in islets of COVID-19 pancreatic autopsy samples

(A) Representative images illustrating morphology marker and selection of ROIs using GeoMx platform. 004, islet area; 005, ductal area; 006, exocrine area. Scale bars, 1 mm or 50 µm.

(legend continued on next page)

diagnosis compared with those without COVID-19. Studies reported a heightened T1D and type 2 diabetes (T2D) incidence rates after the beginning of pandemic, surpassing the pre-pandemic period.[7–9] In addition to severe acute respiratory syndrome coronavirus (SARS-CoV-2), a number of studies suggest the correlation between viral infections and T1D,[10] including enteroviruses[11] such as coxsackievirus B,[12,13] as well as rotavirus,[14] mumps virus,[15] and cytomegalovirus.[16] Coxsackievirus B4 (CVB4), a positive-sense single-stranded RNA virus, isolated from newly diagnosed T1D patients, could infect and induce destruction of human islet cells *in vitro*.[17]

In infectious diseases, multiple mechanisms contribute to the observed host injury. Our group and others discovered that SARS-CoV-2 infection induces the transdifferentiation of human β cells[18] and damage of β cells.[19,20] In addition, the accumulation of macrophages has been reported in the lung[21] and heart[22] of COVID-19 patients. Further insight would benefit from robust human models to explore immune-cell-mediated host damage. Human pluripotent stem cells (hPSCs)[23] provide a powerful *in vitro* platform for studying disease mechanisms, developing cell therapy approaches, and drug screening.[24–26] Many efforts have applied hPSC-based platforms to study SARS-CoV-2 tropism[27] and host responses. Recently, we performed a two-dimensional (2D) co-culture system utilizing hPSC-derived cardiomyocytes and macrophages and identified a Janus kinase (JAK) inhibitor that effectively thwarts macrophage-mediated damage to cardiac cells.[22]

In this study, we applied spatial multi-omics assays to comprehensively analyze pancreatic autopsy samples of COVID-19 patients and identified the accumulation of proinflammatory macrophages in COVID-19 samples. Single-cell RNA sequencing (scRNA-seq) analysis confirmed the activation of proinflammatory macrophages and enrichment of the pyroptotic pathway in β cells of human islets exposed to SARS-CoV-2 or CVB4 viruses. Next, we developed a vascularized macrophage-islet (VMI) organoid model containing hPSC-derived endocrine cells, macrophages, and endothelial cells and found that proinflammatory macrophages induced β cell pyroptosis through the secretion of interleukin-1β (IL-1β) and interaction with β cells via the TNFSF12 (TNF superfamily member 12)-TNFRSF12A (the TNF receptor superfamily member Fn14) pathway. This study not only establishes a VMI organoid model to study macrophage-mediated host damage but also identifies the previously unknown role of TNFSF12-TNFRSF12A-mediated pyroptosis in β cell damage in infectious diseases.

RESULTS

Spatial multi-omics analysis to identify the activation of proinflammatory macrophages in pancreatic autopsy samples of COVID-19 patients

To systematically analyze the pancreatic damage of COVID-19 patients, we collected pancreatic autopsy samples from 7 COVID-19 patients and 8 age- and gender-matched control subjects (Table S1). GeoMx multi-omics assays were applied to analyze two adjacent tissue sections of each donor, providing paired analysis of changes at both transcriptome and protein levels (Figure S1A). For GeoMx analysis, we selected 6 regions of interests (ROIs) in the islet area, 3 ROIs in ductal area, and 3 ROIs in exocrine area per sample (Figures 1A and S1A). Through morphology marker staining (insulin [INS]/pan cytokeratin [Pan-CK]/TOTO-3 [Thiazole Red Homodimer]), we observed no obvious change of islet areas, but a lower percentage of INS^+ cells within islets of COVID-19 samples compared with control samples (Figures 1B and 1C), which is consistent with the previous reports.[18] Three-dimensional (3D) principal-component analysis (PCA) of whole transcriptome sequencing data from ROIs in the islet areas showed distinct transcriptional profiles in COVID-19 samples separated from control samples (Figure 1D). Pathway analysis of differentially expressed (DE) genes between ROIs in islet areas from COVID-19 and control samples highlighted viral-infection-associated pathways, such as viral mRNA translation, influenza infection, interferon (IFN) α/β signaling pathways, and stress-associated pathways, such as cellular response to stress or external stimuli pathways, and Toll-like receptor 2 (TLR2) cascade pathway (Figure 1E). Consistently, PCA of ROIs in ductal and exocrine areas also showed separation between COVID-19 and control samples (Figures S1B and S1C). Moreover, pathway analysis of DE genes between ROIs in ductal and exocrine areas in COVID-19 and control samples also revealed the enrichment of IFN signaling pathways in COVID-19 samples (Figures S1D and S1E).

To further analyze the changes in immune cell composition, we conducted CIBERSORT analysis (an analytical tool to impute gene expression profiles and estimate the abundances of cell

(B) Representative images illustrating the insulin (INS) staining in COVID-19 ($N = 7$) and control ($N = 8$) pancreatic autopsy samples. Dotted lines encircle the islet regions. Scale bars, 75 μm.
(C) Quantification of areas of islets and percentages of INS^+ β cells per islet in COVID-19 ($N = 7$) and control ($N = 8$) pancreatic autopsy samples.
(D) 3D PCA plot of data from human islet areas of COVID-19 ($N = 7$) and control ($N = 8$) pancreatic autopsy samples.
(E) Volcano plot of transcriptome sequencing data highlighting the pathways enriched in human islet areas of COVID-19 ($N = 7$) versus control ($N = 8$) pancreatic autopsy samples.
(F) Heatmap of the CIBERSORT analysis of immune cells (LM22) using the GeoMx whole transcriptome sequencing data of human islet areas of COVID-19 ($N = 7$) and control ($N = 8$) pancreatic autopsy samples.
(G) Normalized counts (log_2) of marker proteins associated with macrophages from human islet areas of COVID-19 ($N = 7$) and control ($N = 8$) pancreatic autopsy samples. Each dot represents one count in each ROI.
(H) Boxplot of normalized counts of macrophage-associated targets in human islet areas of COVID-19 ($N = 7$) and control ($N = 8$) pancreatic autopsy samples. Each dot represents one count in each ROI.
(I and J) Immunohistochemistry staining (I) and quantification (J) of CD80 in COVID-19 ($N = 3$) and control ($N = 3$) pancreatic autopsy samples. Dotted lines encircle the regions of the islets. Scale bars, 20 μm.
p values were calculated by unpaired two-tailed Student's t test. n.s., no significance; $^*p < 0.05$, $^{**}p < 0.01$, $^{***}p < 0.001$.
See also Figure S1.

CellPress
OPEN ACCESS

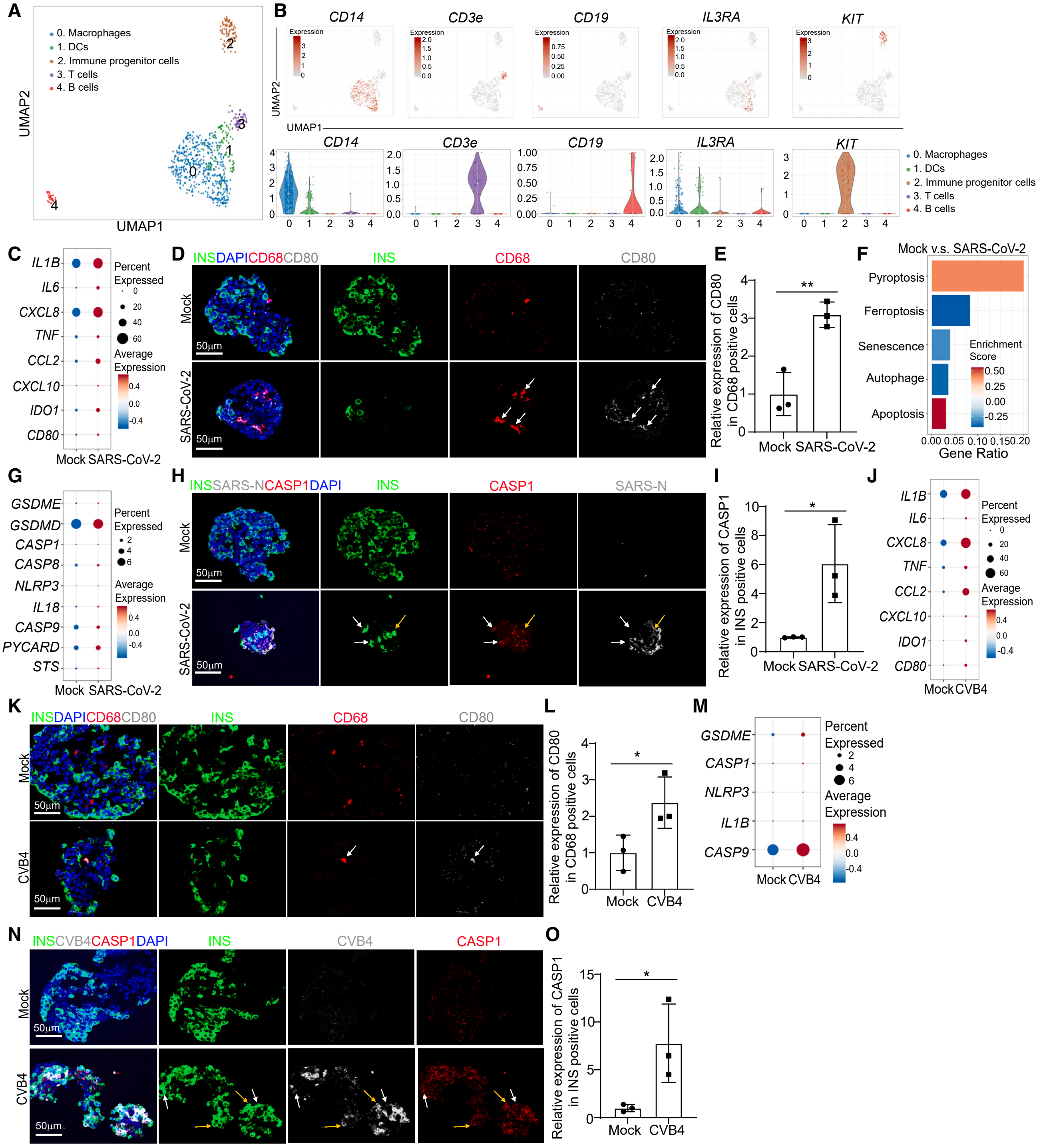

Figure 2. Single-cell RNA-seq analysis of human islets upon SARS-CoV-2 or CVB4 exposure

(A) UMAP of immune cell populations in human islets exposed to mock, SARS-CoV-2 (MOI = 1), or CVB4 (2×10^6 PFU/mL).

(B) UMAP and violin plots of immune cell markers.

(C) Dot plot analysis of proinflammatory-macrophage-associated genes in macrophages of human islets exposed to mock or SARS-CoV-2 (MOI = 1).

(D and E) Confocal images (D) and quantification (E) of the relative expression of CD80 in CD68$^+$ cells in human islets exposed to mock or SARS-CoV-2 (MOI = 0.5). The white arrows highlight the CD68$^+$CD80$^+$ cells. Scale bars, 50 μm.

(F) Pathway enrichment analysis of cell death pathways in β cell cluster of human islets exposed to mock or SARS-CoV-2 (MOI = 1).

(G) Dot plot analysis of pyroptosis-associated genes in β cell cluster of human islets exposed to mock or SARS-CoV-2 (MOI = 1).

(legend continued on next page)

types within a mixed cell population, using gene expression data) of transcriptome profiles from COVID-19 and control samples. Within the ROIs in islet areas of the 7 COVID-19 samples, we found 4 samples (#4–#7) enriched with proinflammatory macrophages and 2 samples (#2–#3) enriched with monocytes (Figure 1F). The enrichment of monocytes or proinflammatory macrophages was not dependent on the pre-existing T2D condition of the subjects (Figure S1F). Consistently, we detected enrichment of proinflammatory macrophages in ductal ROIs in 4 (#4–#7) out of 7 COVID-19 samples, and monocyte enrichment in ductal ROIs in 1 (#3) out of 7 COVID-19 samples (Figure S1G). In exocrine ROIs, we observed enrichment of proinflammatory macrophages in 4 (#4–#7) out of 7 COVID-19 samples (Figure S1H).

We further conducted GeoMx protein assays and found that macrophages were enriched in islet ROIs of COVID-19 samples compared with control samples (Figures 1G and 1H), while T cells, NK cells, B cells, and neutrophils were not enriched (Figure S1I). Moreover, the proteins related to T cell activation were not increased in islet ROIs of COVID-19 samples compared with control samples (Figure S1J). Notably, CD44, previously reported to regulate the TLR2-mediated macrophage activation and proinflammatory responses,[28,29] was also found to be significantly increased in ROIs in islet, ductal, and exocrine areas of COVID-19 samples (Figures 1G and S1K). CD163, which functions as the scavenger receptor, is highly upregulated in infiltrating macrophages in sites of inflammation.[30,31] Soluble CD163 was also identified as a biomarker of macrophage activation and associated with T2D mellitus (T2DM), INS resistance, and β cell dysfunction.[32] Finally, immunohistochemistry validated the accumulation of both $CD163^+$ macrophages and $CD80^+$ proinflammatory macrophages in pancreatic tissues of COVID-19 patients (Figures 1I, 1J, S1L, and S1M).

scRNA-seq analysis identifies activation of proinflammatory macrophages and β cell pyroptosis in SARS-CoV-2- or CVB4-exposed human islets

To further explore the status of macrophages upon virus exposure in human islets, we performed scRNA-seq of human islets upon exposure of SARS-CoV-2 or CVB4. Uniform manifold approximation and projection (UMAP) analysis revealed nine cell clusters within human islets (Figures S2A and S2B). In our previous publication, we have already characterized the SARS-CoV-2-infected human islets.[18] Here, we further characterized the CVB4-infected human islets. UMAP and violin plot showed the high expression of CVB4 virus *polyprotein* in endocrine cells (β cells, α cells, and δ cells), as well as mesenchymal cells, immune cells, and endothelial cells (Figures S2C and S2D). Immunostaining confirmed the colocalization of enterovirus (CVB4) and endocrine cell markers, including INS (β cells), glucagon (GCG, α cells), and somatostatin (SST, δ cells) (Figures S2E–S2I).

We then focused on the immune cell population and performed sub-clustering analysis, identifying five sub-clusters (Figure 2A). UMAP and violin plots confirmed the expression of marker genes for each subpopulation (Figure 2B). We compared the transcriptional profiles of macrophages and found increased expression of proinflammatory-macrophage-associated genes, including *IL1B*, *IL6*, *CXCL8*, and *TNF* in macrophages of human islets upon SARS-CoV-2 exposure (Figure 2C). Immunostaining also confirmed the activation of proinflammatory macrophages in human islets upon SARS-CoV-2 infection (Figures 2D, 2E, and S2J–S2M). We further analyzed several cell-death-associated pathways within β cell cluster of human islets exposed to SARS-CoV-2 virus. Interestingly, we found the activation of pyroptosis and apoptosis pathways in β cells of human islets exposed to SARS-CoV-2 (Figure 2F). Our previous studies have reported the activation of apoptosis of SARS-CoV-2-infected β cells.[27] In the current study, we focused on β cell pyroptosis. Dot plot analysis showed increased expression levels of pyroptosis-associated genes in both SARS-CoV-2+ and SARS-CoV-2− β cells of human islets exposed to SARS-CoV-2 (Figures 2G and S3A). Immunostaining further confirmed the increased expression of cleaved caspase-1 (CASP1) in β cells of human islets upon SARS-CoV-2 infection (Figures 2H and 2I). In addition, we found enrichment of pyroptosis pathway in other endocrine cell clusters (α and δ cell clusters) of human islets exposed to SARS-CoV-2 (Figure S3B). Moreover, the autophagy pathway was enriched in mesenchymal cell cluster, while ferroptosis and apoptosis pathways were enriched in endothelial cell cluster of human islets exposed to SARS-CoV-2 (Figure S3B).

Next, we analyzed human islets exposed to CVB4 virus. Similar to SARS-CoV-2 virus, CVB4 virus exposure also induced activation of proinflammatory macrophages (Figures 2J–2L and S3C–S3F). Furthermore, dot plots showed increased expression of pyroptotic-pathway-associated genes in both CVB4+ and CVB4− β cells of human islets upon CVB4 infection (Figures 2M and S3G). Finally, immunostaining confirmed the increased expression of CASP1 in β cells of human islets upon CVB4 infection (Figures 2N and 2O). Together, these data demonstrate the activation of proinflammatory macrophages

(H and I) Confocal images (H) and quantification (I) of the relative expression of CAPS1 in human islets exposed to mock or SARS-CoV-2 (MOI = 0.5). The yellow arrows highlight the expression of CASP1 in $SARS\text{-}N^+INS^+$ cells while the white arrows highlight the expression of CASP1 in $SARS\text{-}N^-INS^+$ cells. Scale bars, 50 μm.
(J) Dot plot analysis of proinflammatory-macrophage-associated genes in macrophage cluster of human islets exposed to mock or CVB4 (2×10^6 PFU/mL).
(K and L) Confocal images (K) and quantification (L) of the relative expression of CD80 in $CD68^+$ cells in human islets exposed to mock or CVB4 (2×10^6 PFU/mL). The white arrows highlight the $CD68^+CD80^+$ cells. Scale bars, 50 μm.
(M) Dot plot analysis of pyroptosis-pathway-associated genes in β cell cluster of human islets exposed to mock or CVB4 (2×10^6 PFU/mL).
(N and O) Fluorescent images (N) and quantification (O) of the relative expression of CAPS1 in human islets exposed to mock or CVB4 (2×10^6 PFU/mL). The yellow arrows highlight the expression of CASP1 in $SARS\text{-}N^+INS^+$ cells while the white arrows highlight the expression of CASP1 in $SARS\text{-}N^-INS^+$ cells. Scale bars, 50 μm.
$n = 3$ independent biological replicates. Data were presented as mean ± STDEV. p values were calculated by unpaired two-tailed Student's t test. $^*p < 0.05$, $^{**}p < 0.01$.
See also Figures S2 and S3.

CellPress
OPEN ACCESS

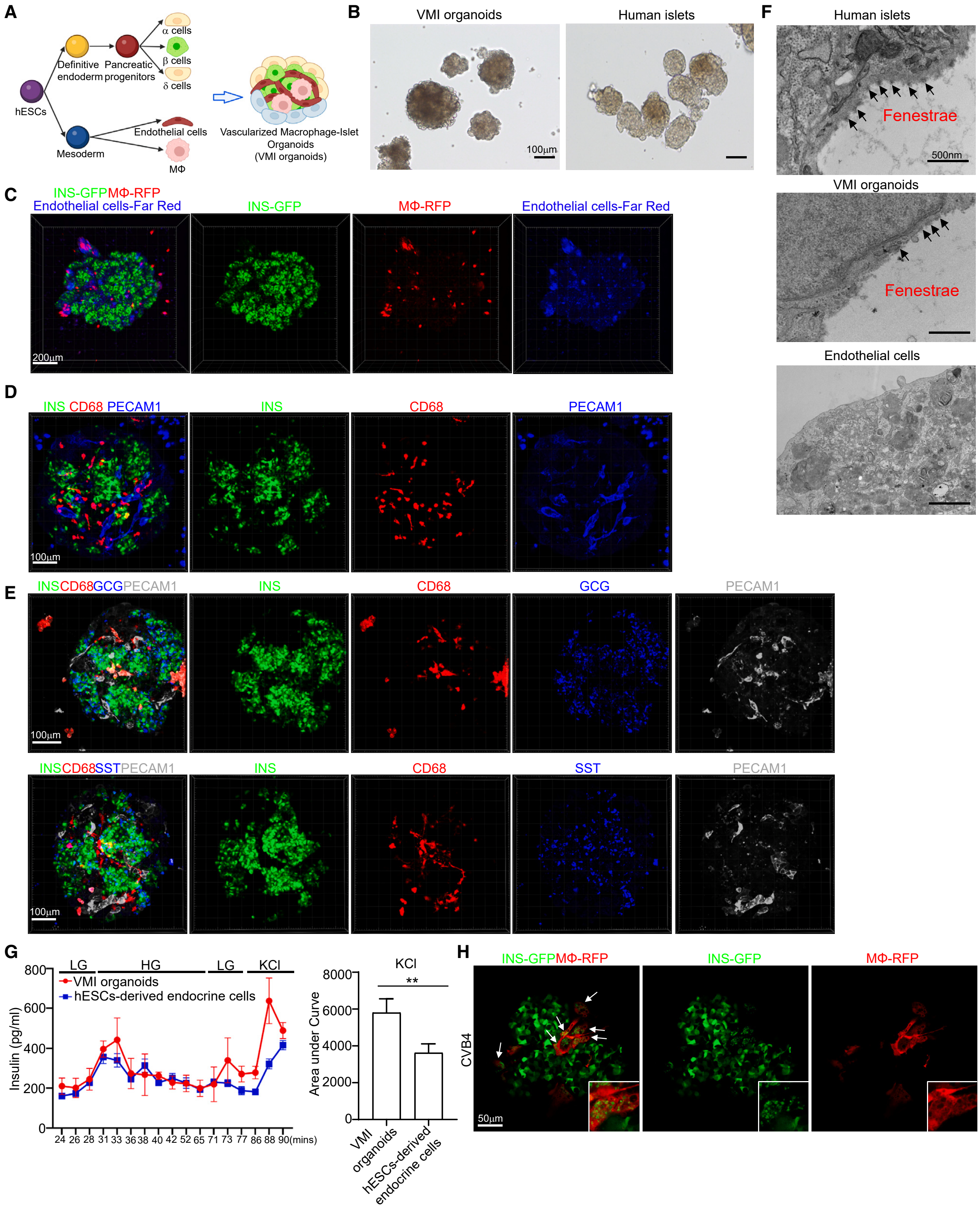

Figure 3. Construction of hPSC-derived VMI organoids

(A) Schematic representation of VMI organoids construction.

(B) Phase contract images of VMI organoids at day 14 after reaggregation and human islets. Scale bars, 200 μm.

(legend continued on next page)

and β cell pyroptosis in human islets exposed to SARS-CoV-2 or CVB4 viruses.

To further analyze the changes of immunogenicity profiles of β cells in response to viral infection, we analyzed the expression of human leukocyte antigen (HLA) molecules and autoantigen-associated genes. We observed a pattern of the increased expression of HLA class I genes in β cells of human islets exposed to SARS-CoV-2 versus mock (Figure S3H). In contrast, there was a trend of reduced expression of HLA class I genes in β cells of human islets exposed to CVB4 versus mock (Figure S3I). In terms of autoantigen expression, we observed a similar increase in the expression of *GAD2* and *IAPP* in β cells of human islets exposed to SARS-CoV-2 or CVB4 compared with mock conditions. For *CHGA* and *SLC30A8*, we noted different trends of expression, which increased in β cells of human islets exposed to SARS-CoV-2 but decreased in β cells of human islets exposed to CVB4 (Figures S3J and S3K). Moreover, we also examined the genes related to antigen presentation and found an increased expression of antigen-presentation-associated genes in β cells of human islets exposed to SARS-CoV-2 and a reduced expression of them in β cells of human islets exposed to CVB4 (Figures S3L and S3M).

Construction of a VMI organoid

To determine whether β cell pyroptosis is caused by proinflammatory macrophages activation, we constructed a VMI organoid model (Figure 3A). First, we differentiated MEL-1$^{INS/GFP}$ human embryonic stem cells (hESCs) into pancreatic endocrine cells (Figure S4A). At day 16, we detected the robust generation of INS$^+$ β cells, GCG$^+$ α cells, and SST$^+$ δ cells (Figure S4B). H9 hESCs were differentiated toward macrophages that expressed CD11B, CD14, and CD206, but not CD80 (Figures S4C and S4D). Functional assays confirmed that hESC-derived macrophages can engulf bacteria, indicating that they exhibited phagocytic activity similar to primary human macrophages (Figure S4E). Human islets are highly vascularized and endothelial cells play an important role in systemic inflammatory responses[33,34] as well as pancreatic cell development.[35–37] Thus, we decided to add endothelial cells to VMI organoids. ETV2 was reported to promote the development of endothelial cells.[38,39] Here, we overexpressed ETV2 to promote the differentiation and function of endothelial cells from H1 hESCs[39–41] (Figure S4F). Reverse-transcriptase quantitative PCR (RT-qPCR) confirmed the overexpression of ETV2 in H1 hESCs (Figure S4G) and immunostaining confirmed that differentiated endothelial cells expressed latelet endothelial cell adhesion molecule-1 (PECAM1, also known as CD31) (Figure S4H).

After optimizing the culture medium and cell ratios, we mixed the hESC-derived endocrine cells, unstimulated macrophages, and endothelial cells in 3D culture to form organoids (Figure 3A). The VMI organoids exhibited similar size and morphologies as primary human islets (Figure 3B). To perform live imaging of the VMI organoid, we labeled cells with fluorescent reporters or CellTrace dye. Pancreatic endocrine cells were derived from MEL-1$^{INS/GFP}$ hESCs, allowing real-time monitoring of INS-GFP$^+$ β cells. Macrophages were derived from red fluorescent protein-labeled H9 (RFP-H9) hESCs and purified using magnetic sorting before organoid formation. Additionally, H1-hESC-derived endothelial cells were selected by magnetic sorting and the purified endothelial cells were labeled with CellTrace proliferative far-red dye before forming 3D organoids. 3D live cell imaging confirmed the presence of INS-GFP$^+$ β cells, RFP$^+$ macrophages, and far red$^+$ endothelial cells in VMI organoids (Figure 3C; Video S1). Immunostaining of VMI organoids confirmed the presence of INS$^+$ β cells, CD68$^+$ macrophages, and PECAM1$^+$ endothelial cells (Figure 3D; Video S2). Most of the INS$^+$ β cells in VMI organoids co-expressed NKX6.1, a key transcription factor of β cells (Figure S4I; Video S3). Immunostaining further confirmed the presence of GCG$^+$ α cells and SST$^+$ δ cells in VMI organoids (Figure 3E; Videos S4 and S5).

Next, we used different assays to determine whether the cells in VMI organoids closely resembled the cells in primary islets. Initially, electron microscopy (EM) was used to observe fenestrae, which are transcellular pores found in endothelial cells facilitating the transfer of substances between blood and the extravascular space.[42] Indeed, the fenestrations were detected in the endothelial cells of both primary human islets and VMI organoids, but not in separately cultured endothelial cells (Figure 3F). We performed an acetylated low-density lipoprotein (Ac-LDL) uptake assay to assess the function of endothelial cells in VMI organoids. Ac-LDL can bind to the receptor on the surface of vascular endothelial cells, facilitating the delivery of cholesterol via endocytosis.[43,44] We found colocalization of Ac-LDL with PECAM1$^+$ endothelial cells (Figure S4J). Then, we performed dynamic glucose-stimulated INS secretion (GSIS) to examine the secretion of INS upon glucose or KCl stimulation. We found increased INS expression in VMI organoids compared with separately cultured hESC-derived endocrine cells under both high-glucose and KCl-stimulation conditions. The amount of INS secreted upon KCl stimulation was significantly higher in

(C) Composite z stack confocal images of live VMI organoids at day 14 after reaggregation. β cells, INS-GFP; macrophages, RFP; and endothelial cells, far red. Scale bars, 200 μm.
(D) Composite z stack confocal images of VMI organoids at day 14 after reaggregation stained with antibodies against INS, CD68, and PECAM1 (CD31). Scale bars, 100 μm.
(E) Composite z stack confocal images of VMI organoids at day 14 after reaggregation stained with antibodies against INS, CD68, GCG, SST, and PECAM1 (CD31). Scale bars, 100 μm.
(F) Transmission electron microscope (TEM) images of human islets, VMI organoids at day 14 after reaggregation, and endothelial cells without reaggregation. Arrows indicate fenestrae. Scale bars, 500 nm.
(G) Dynamic glucose stimulated insulin secretion of VMI organoids at day 14 after reaggregation and hPSC-derived endocrine cells. Low glucose (LG), 2 mM D-glucose; high glucose (HG), 20 mM D-glucose; KCl, 30 mM KCl. Quantification was performed using the areas under curve of KCl stimulation from 86 to 90 min.
(H) Composite z stack confocal images of VMI organoids at day 7 after reaggregation upon CVB4 infection (2×10^6 PFU/mL). β cells, INS-GFP; macrophages, RFP. Scale bars, 50 μm. Arrows highlight RFP$^+$ macrophages that have phagocytosed damaged INS-GFP$^+$ β cells.
$n = 3$ independent biological replicates. Data were presented as mean ± STDEV. p values were calculated by unpaired two-tailed Student's t test. $^{**}p < 0.01$.
See also Figure S4.

CellPress
OPEN ACCESS

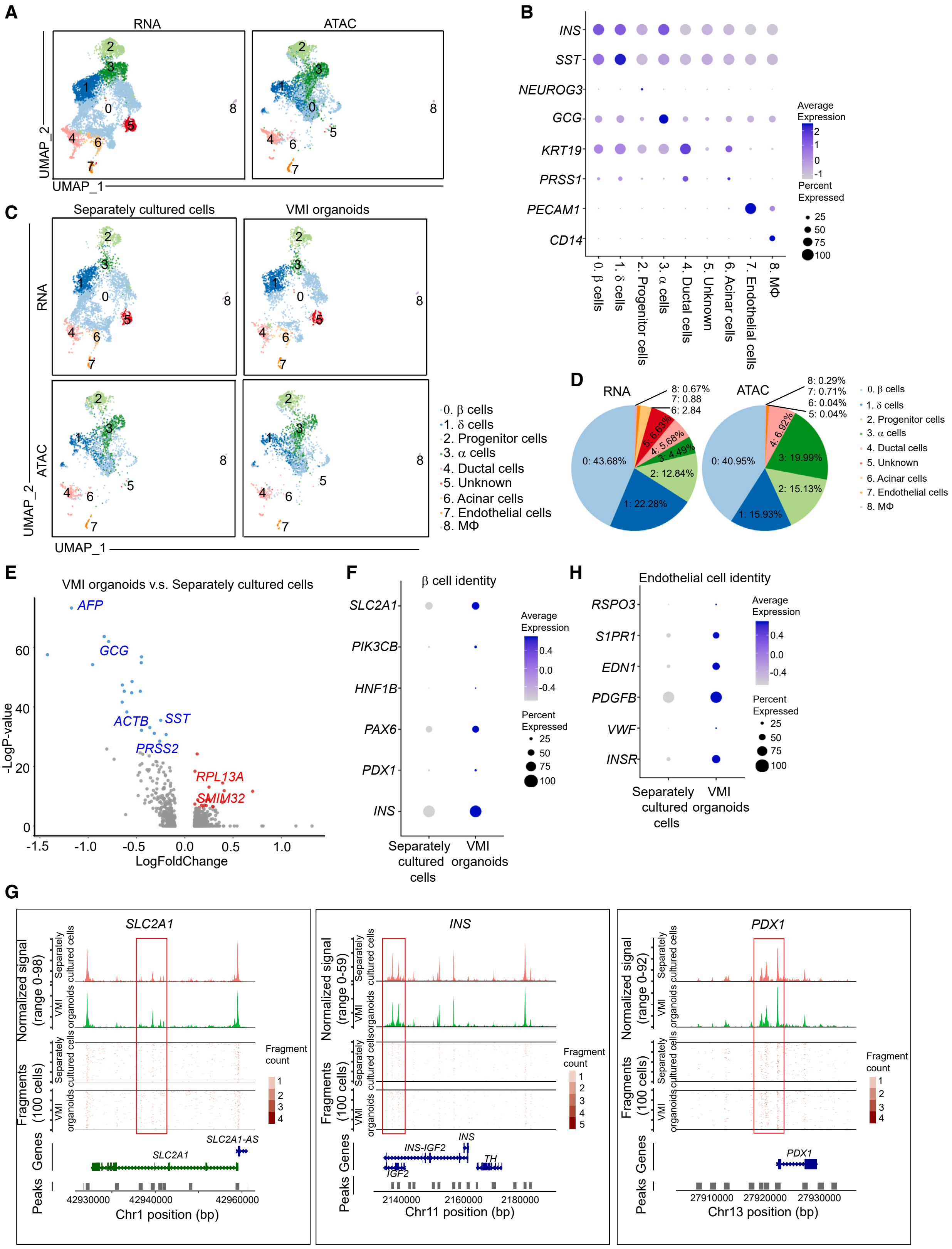

(legend on next page)

VMI organoids than separately cultured hESC-derived endocrine cells (Figure 3G). Besides, we also found a decrease in GCG secretion in VMI organoids compared with separately cultured endocrine cells (Figure S4K). To further elevate the function of β cells upon low-glucose and high-glucose stimulation, we performed dynamic calcium Flu4 imaging. We detected dynamic calcium mobilization in cells of VMI organoids upon high-glucose stimulation (Figure S4L; Video S6). Together, the data indicate that the pancreatic β cells and endothelial cells in VMI organoids are functionally more mature than cells that are cultured separately.

Finally, we exposed the VMI organoids to CVB4 virus and found macrophages engulfing the damaged β cells upon virus infection (Figure 3H; Video S7). To examine monocyte infiltration, we created organoids containing endocrine cells and endothelial cells (VI organoids) and monitored monocyte infiltration upon CVB4 infection. We first added monocytes to VI organoids, then introduced CVB4 and conducted live cell imaging at 24 and 48 hours post infection (hpi). We did not find obvious infiltration of monocytes into VI organoids (Figure S4M).

Single-cell multi-omics analysis of VMI organoids

We then performed scRNA-seq and single-nucleus assay for transposase-accessible chromatin using sequencing (snATAC-seq) to compare the cell compositions and transcriptional and epigenetic profiles of VMI organoids and separately cultured cells[45] (Figure 4A). Cells that were cultured in separate plates but mixed together before library preparation were compared with cells in VMI organoids (Figure S4N). UMAP analysis identified 9 cell clusters (Figure 4A). Dot plot of scRNA-seq analysis (Figure 4B) and integrative genomics viewer (IGV) plot of snATAC-seq (Figure S4O) confirmed the marker gene expression in each cluster. Consistent with previous studies,[46–48] *GCG* expression was detected in β cell cluster and *INS* expression was detected in α and δ cell clusters, suggesting the immature status of hESC-derived endocrine cells. Next, we compared the cells in VMI organoids with separately cultured cells (Figure 4C). The pie chart shows the relative proportions of major cell types in VMI organoids (Figure 4D). Volcano plot analysis of gene expression in β cell cluster showed decreased expression of non-β cell-associated genes, *AFP*,[49] *GCG*, *SST*, *ACTB*, and *PRSS2*, and increased expression of β cell-associated genes *RPL13A*[50] and *SMIM32*[51] in the β cell cluster of VMI organoids compared with separately cultured cells (Figure 4E). Dot plots and violin plots analysis also showed that genes associated with β cell identity and function, including *SLC2A1*,[52–54] *PIK3CB*,[55] *HNF1B*, *PAX6*, *PDX1*, and *INS*, are relatively increased in the β cell cluster of VMI organoids (Figures 4F and S4P). Consistently, snATAC-seq analysis showed increased open chromatin accessibility peaks of *SLC2A1*,[52–54] *INS*, and *PDX1* in β cells of VMI organoids compared with separately cultured cells, which might indicate the potential of increased gene expression of *SLC2A1*, *INS*, and *PDX1* (Figure 4G). In addition, dot plots and violin plots also revealed the upregulation of genes associated with endothelial cell function in endothelial cell cluster of VMI organoids compared with separately cultured cells, including *INSR*,[42] *VWF*,[56] *PDGFB*,[57] *EDN1*,[58] *S1PR1*,[59] and *RSPO3*[60] (Figures 4H and S4Q).

Proinflammatory macrophages cause β cell pyroptosis

We have shown the activation of proinflammatory macrophages, as well as upregulation of the pyroptotic pathway in β cell cluster of human islets exposed to SARS-CoV-2 or CVB4 viruses. Here, we also detected the activation of proinflammatory macrophages as well as β cell pyroptosis in VMI organoids exposed to SARS-CoV-2 or CVB4 viruses (Figures S5A–S5D). To determine whether proinflammatory macrophages cause β cell pyroptosis, we constructed the VMI organoids with proinflammatory or unstimulated macrophages. First, LPS and IFN-γ were used to stimulate macrophages into a proinflammatory status (Figure S5E). Both RNA-seq and ELISA analysis confirmed the increased expression of proinflammatory-associated genes and cytokines, including IL-1β and IL-6 in proinflammatory macrophages (Figures S5F and S5G). Then, we constructed VMI organoids using either unstimulated or proinflammatory macrophages. VMI organoids containing proinflammatory macrophages showed decreased expression levels of INS compared with VMI organoids containing unstimulated macrophages (Figures 5A and 5B; Videos S8 and S9). We collected the supernatant of VMI organoids containing proinflammatory or unstimulated macrophages and confirmed the increased expression of IL-1β, IL-6, and tumor necrosis factor alpha (TNF-α) in the supernatant of VMI organoids containing proinflammatory macrophages (Figure 5C).

Next, scRNA-seq and snATAC-seq were performed to analyze the VMI organoids containing proinflammatory or unstimulated macrophages. Consistent with the previous analysis, 9 cell clusters were identified in VMI organoids. UMAP showed a decrease of the β cell cluster in VMI organoids with proinflammatory macrophages, which was also confirmed by quantification of the percentage of β cells (Figures 5D and 5E). Moreover, a volcano plot comparing the β cell cluster of VMI organoids containing proinflammatory macrophages to that of organoids containing unstimulated macrophages showed the downregulation of

Figure 4. Single-cell multi-omics analysis of VMI organoids

(A) Integrative UMAP of scRNA-seq and snATAC-seq analysis of VMI organoids at day 7 after reaggregation and separately cultured cells.
(B) Dot plot displaying cell markers of each cluster using scRNA-seq dataset.
(C) Individual UMAP of scRNA-seq and snATAC-seq analysis of VMI organoids at day 7 after reaggregation and separately cultured cells.
(D) Pie chart shows the relative percentages of each cell types in VMI organoids at day 7 after reaggregation.
(E) Volcano plot of DE genes in β cell cluster of VMI organoids at day 7 after reaggregation versus separately cultured cells.
(F) Dot plot analysis of β cell-associated genes in β cell cluster of VMI organoids at day 7 after reaggregation and separately cultured cells.
(G) Chromatin accessibility signals of *SLC2A1*, *INS*, and *PDX1* in β cell cluster of VMI organoids at day 7 after reaggregation and separately cultured cells. The normalized signal shows the averaged frequency of sequenced DNA fragments within a genomic region. The fragment shows the frequency of sequenced fragments within a genomic region for individual cells.
(H) Dot plot analysis of endothelial-cell-associated genes in endothelial cell cluster of VMI organoids at day 7 after reaggregation and separately cultured cells.
See also Figure S4.

CellPress
OPEN ACCESS

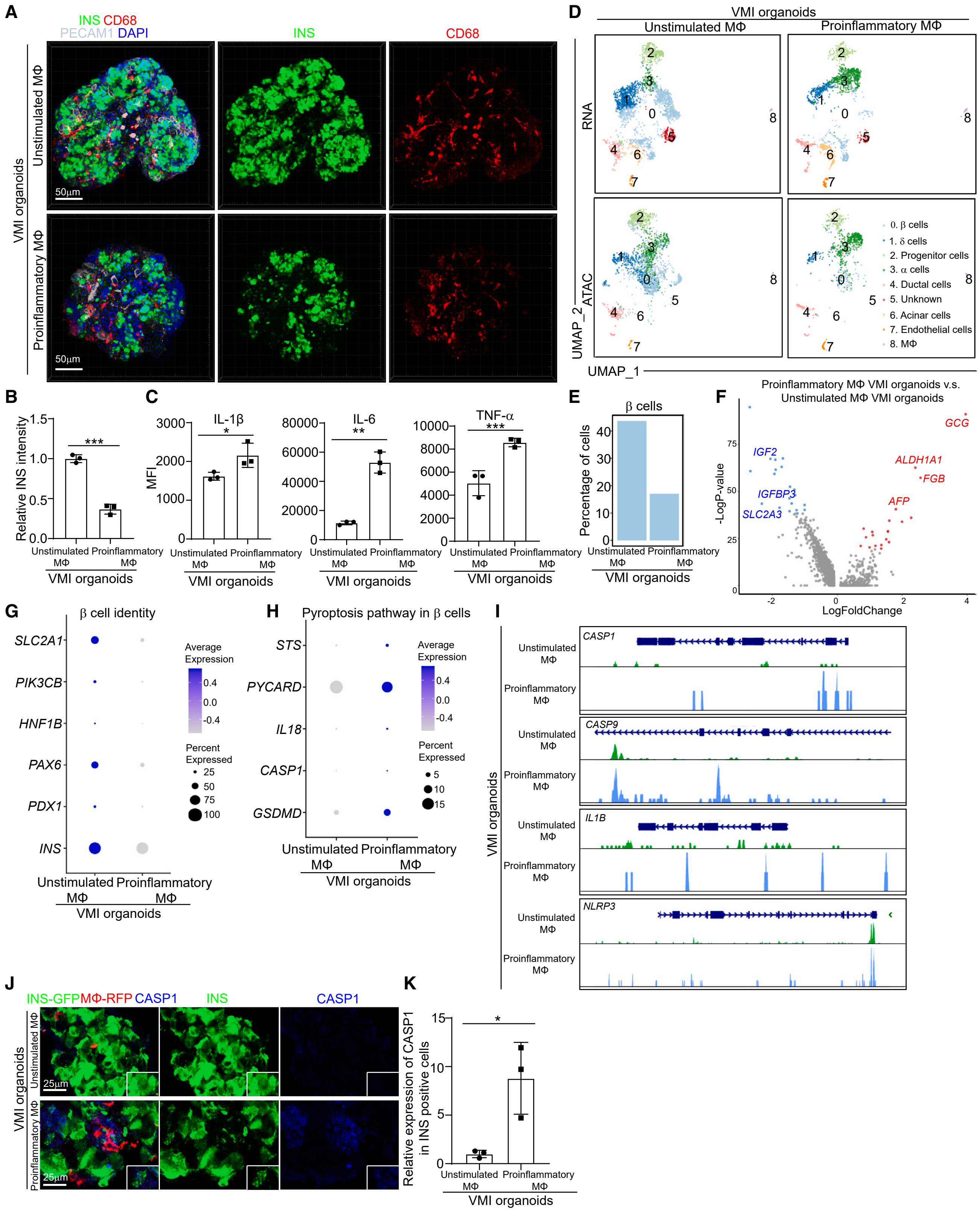

Figure 5. Construction and multi-omics analysis of VMI organoids containing unstimulated and proinflammatory macrophages

(A and B) Composite z stack confocal images (A) and quantification (B) of INS intensity in INS$^+$ cells of VMI organoids at day 7 after reaggregation containing unstimulated or proinflammatory macrophages stained with the antibodies against INS, CD68, and PECAM1 (CD31). Scale bars, 50 μm.

(legend continued on next page)

genes associated with β cell identity and function, including the decreased expression levels of *IGF2*,[61] *IGFBP3*,[62] and *SLC2A3*,[63] and upregulation of genes associated with non-β cell identity and function, *GCG*, *ALDH1A1*,[64] *FGB*,[65] and *AFP*[49] (Figure 5F). Dot plot analysis further confirmed the downregulation of genes associated with β cell identity and function, including *SLC2A1*,[52–54] *PIK3CB*,[55] *HNF1B*, *PAX6*, *PDX1*, and *INS*, in the β cluster of VMI organoids containing proinflammatory macrophages compared with VMI organoids containing unstimulated macrophages (Figure 5G). Consistently, snATAC-seq analysis also showed decreased open chromatin accessibility peaks of *INS* and *PDX1* in β cells of VMI organoids containing proinflammatory macrophages (Figure S5H). Furthermore, dot plot analysis of scRNA-seq analysis also showed increased expression of pyroptotic-pathway-associated genes in the β cluster of VMI organoids containing proinflammatory macrophages (Figure 5H). Consistent with increased expression of pyroptotic-pathway-associated genes, snATAC-seq analysis showed increased open chromatin accessibility peaks of *CASP1*, *CASP9*, *IL1B*, and *NLRP3* in the β cell cluster of VMI organoids containing proinflammatory macrophages (Figure 5I). Upregulation of the pyroptotic pathway in the β cell cluster of VMI organoids containing proinflammatory macrophages was further confirmed by immunostaining using an antibody against CASP1 (Figures 5J and 5K). Apart from β cell pyroptosis, we did not observe β cell dedifferentiation in VMI organoids with proinflammatory macrophages (Figure S5I). Together, these data suggest that proinflammatory macrophages can induce β cell pyroptosis.

Mechanistic studies identify pathways contributing to proinflammatory-macrophage-mediated β cell pyroptosis

To determine the potential mechanisms by which proinflammatory macrophages induce β cell pyropotosis, we performed cell-cell interaction (cell-chat) analysis and focused on the interactions between macrophages to β cells. First, when comparing differential signaling from macrophages to β cells in VMI organoids containing proinflammatory macrophages to VMI organoids containing unstimulated macrophages, we identified four enhanced macrophage-to-β cell interaction pathways, including *TNFSF12-TNFRSF12A*, *SPP1-ITGAV+ITGB1*, *F11R-F11R*, and *DSC2-DSG2* (Figure 6A). Next, we examined cell-cell interactions from macrophages to β cells in human islets exposed to CVB4 virus and also found increased communication probability of the *TNFSF12-TNFRSF12A* pathway (Figure 6B). Furthermore, the expression level of *TNFSF12* was increased in macrophages of human islets exposed to SARS-CoV-2 (Figure 6C). Immunostaining confirmed the increased expression of TNFSF12 in both human islets exposed to viruses and VMI organoids containing proinflammatory macrophages (Figures S6A and S6B). These data indicate that the TNFSF12-TNFRSF12A pathway might contribute to proinflammatory-macrophage-mediated β cell pyroptosis.

To validate the role of TNFSF12-TNFRSF12A in proinflammatory-macrophage-mediated β cells pyroptosis, we treated human islets and VMI organoids containing unstimulated macrophages with TNFSF12 protein and detected increased CAPS1 expression in INS$^+$ β cells in both cases (Figures 6D–6G). Next, we tested TNFSF12 neutralization antibody and found that it partially blocked β cell pyroptosis caused by SARS-CoV-2 or CVB4 exposure (Figures 6H and 6I), suggesting that other factors might also contribute to this process. One candidate is IL-1β, which was detected in the supernatant of VMI organoids containing proinflammatory macrophages and macrophages of human islets exposed to SARS-CoV-2 or CVB4 (Figures 2C, 2J, 5C, S2J, S2K, S3C, and S3D) and was reported to contribute to cell pyroptosis.[66,67] Indeed, IL-1β neutralization antibody partially blocked the increased β cell pyroptosis in human islets exposed to SARS-CoV-2 or CVB4. Furthermore, the combination of IL-1β and TNFSF12 neutralization antibodies showed an add-on/synergistic effect to further decrease the CASP1 expression levels in human islets exposed to SARS-CoV-2 or CVB4 (Figures 6H and 6I) and VMI organoids with proinflammatory macrophages or VMI organoids exposed to SARS-CoV-2 or CVB4 (Figures 6J, 6K, and S6C–S6F). Finally, we stained the pancreatic autopsy samples and confirmed the increased CASP1 expression in COVID-19 samples compared with control samples (Figures 6L and 6M). Moreover, the increased CASP1 expression was independent of T2D conditions (Figures 6L and 6M). GeoMx transcriptomic data also showed increased expression of pyroptosis-associated genes in ROIs of islets in COVID-19 samples compared with control samples (Figure S6G).

(C) Measurements of cytokine secretions in the supernatant of VMI organoids at day 5 after reaggregation containing unstimulated or proinflammatory macrophages.
(D) Integrative UMAP of VMI organoids at day 7 after reaggregation containing unstimulated or proinflammatory macrophages.
(E) Percentage of cells in β cell cluster in VMI organoids at day 7 after reaggregation containing unstimulated or proinflammatory macrophages.
(F) Volcano plot of DE genes in β cell cluster of VMI organoids at day 7 after reaggregation containing proinflammatory versus unstimulated macrophages.
(G) Dot plot analysis of β cell-identity-associated genes in β cell cluster of VMI organoids at day 7 after reaggregation containing unstimulated or proinflammatory macrophages.
(H) Dot plot analysis of pyroptosis-pathway-associated genes in β cell cluster of VMI organoids at day 7 after reaggregation containing unstimulated or proinflammatory macrophages.
(I) Chromatin accessibility signals of *CASP1*, *CASP9, IL1B*, and *NLRP3* in β cell cluster of VMI organoids at day 7 after reaggregation containing unstimulated or proinflammatory macrophages. The normalized signal shows the averaged frequency of sequenced DNA fragments within a genomic region. The fragment shows the frequency of sequenced fragments within a genomic region for individual cells.
(J and K) Immunostaining (J) and quantification (K) of CASP1 staining in INS+ cells of VMI organoids at day 7 after reaggregation containing unstimulated or proinflammatory macrophages. β cells, INS-GFP; macrophages, RFP; endothelial cells, far red. CASP1, gray. Scale bars, 25 μm.
$n = 3$ independent biological replicates. Data were presented as mean ± STDEV. p values were calculated by unpaired two-tailed Student's t test. *$p < 0.01$, **$p < 0.05$, ***$p < 0.001$.
See also Figure S5.

CellPress
OPEN ACCESS

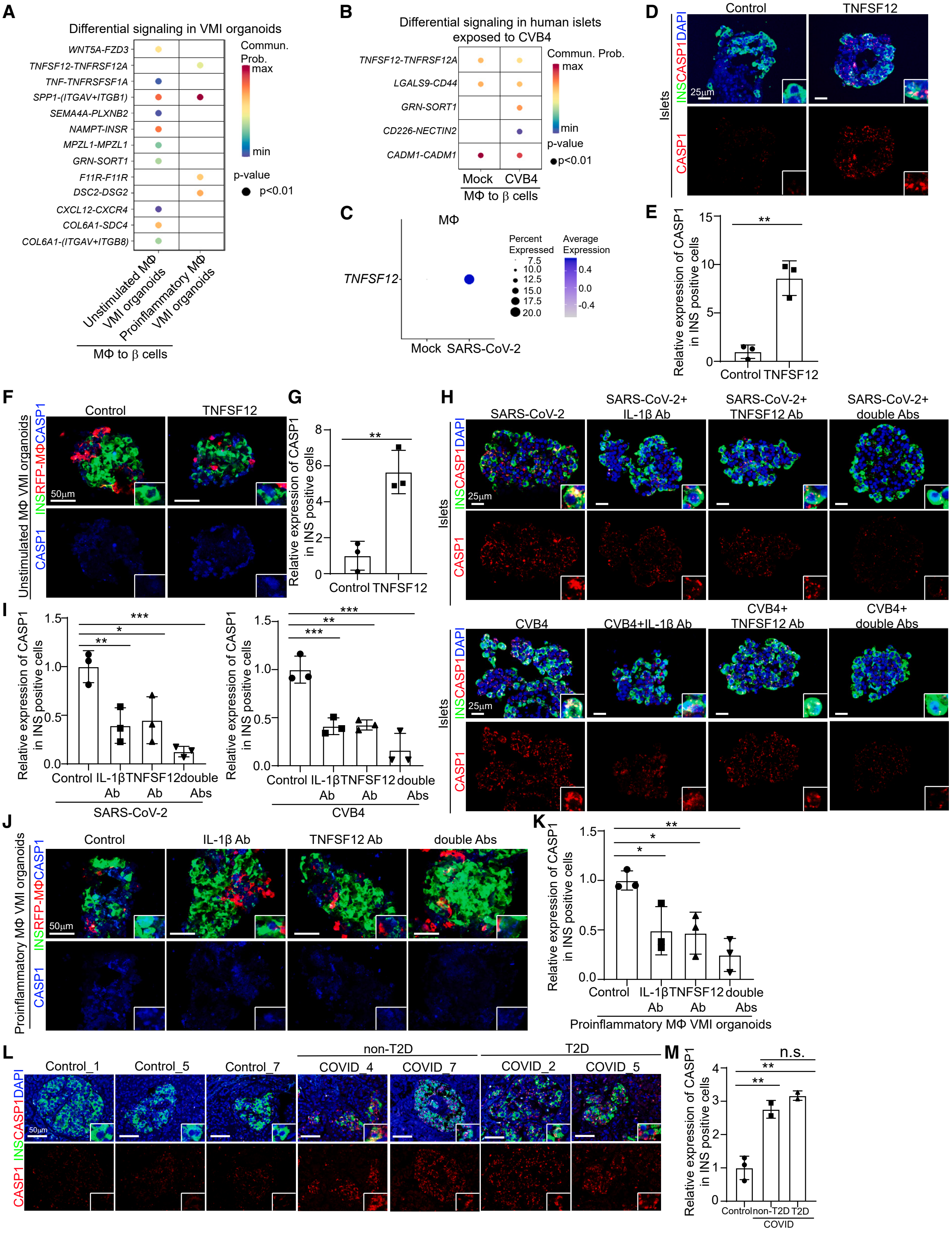

Figure 6. TNFSF12-TNFRSF12A as a candidate pathway that contributes to proinflammatory-macrophage-mediated β cell pyroptosis

(A) Dot plot showed the differential signaling from macrophages to β cells in VMI organoids containing unstimulated or proinflammatory macrophages at day 7 after reaggregation.

(legend continued on next page)

DISCUSSION

Although several spatial transcriptomic analyses have been applied to study COVID-19 autopsy samples, they have focused on lung,[68–70] liver,[71] heart,[72] and placenta.[73] In this study, we used the GeoMx spatial transcriptomics and proteomics platform to comprehensively analyze changes in the immune cell composition and endocrine cell damage of COVID-19 pancreatic samples. Our findings revealed accumulation of proinflammatory macrophages in islets of COVID-19 samples, which highlights the critical role of macrophages in pathological changes observed in host tissues in COVID-19 patients. Previous study has shown that SARS-CoV-2 induces a pro-fibrotic signature in monocytes, which includes CD163, a marker not expressed in homeostatic monocytes.[74] We also see an increase of CD163$^+$ macrophages in islets of COVID-19 samples, which could be an indication of fibrogenic monocyte infiltration. Fibrosis might also play a role in pancreatitis, new-onset diabetes, and thus β cell damage.[75] Fibrotic alterations might be another potential driver of tissue dysfunction besides β cell pyroptosis. Surprisingly, in our spatial transcriptomics data, we did not see an increase of T cells in islets of COVID-19 samples, although both CD4 and CD8 T cells contribute to T1D development.[76,77] Upon thorough examination of published studies, no reports were identified regarding T cell infiltrations in the islets of COVID-19 samples. This underscores the need to impartially assess immune cell accumulation and expand the scope of investigation by examining additional COVID-19 pancreas samples.

In our study, we found that β cells in VMI organoids showed improved maturity. Islet vascular endothelial cells were reported to promote INS production and secretion, as well as β cell proliferation, survival, and maturation, by secreting a variety of growth factors, components of the extracellular matrix (ECM), and other molecules.[78–80] Macrophages exist in the pancreas from the embryonic stage onward. Although the role of macrophages in islet morphogenesis is not well understood, various observations underscore their significance in the formation of the endocrine pancreas, especially in the development of β cells.[81,82]

Although immune-mediated host damage is recognized as a critical factor in various diseases, there is a scarcity of suitable human *in vitro* models. Here, we constructed a hPSC-derived VMI organoid model, which allowed us to dissect molecular mechanisms of macrophage-mediated host damage. Through cell-cell interaction analysis, we found that proinflammatory macrophages induce β cell pyroptosis through the TNFSF12-TNFRSF12A pathway. Previous studies in the context of cholestasis demonstrated that bile acids induce TNFRSF12A expression, subsequently initiating hepatocyte pyroptosis through the nuclear factor κB (NF-κB)/CASP1/gasdermin (GSDMD) signaling pathway.[83] Persistent TNFSF12-TNFRSF12A signaling has been implicated in the pathogenesis of numerous diseases, including atherosclerosis, ischemic stroke, rheumatoid arthritis (RA), and inflammatory bowel diseases.[84,85] Some of the TNFSF12-TNFRSF12A-targeted therapeutic agents are under development for these conditions.[86] Enavatuzumab, BIIB036, and RG7212, the humanized monoclonal antibodies targeting TNFSF12-TNFRSF12A signaling, were tested in patients with tumors.[87–89] BIIB023 was also tested in patients with RA (NCT00771329) and lupus nephritis (NCT01499355) in clinical trials.[90] Here, we identified a previously unknown role of TNFSF12-TNFRSF12A in macrophage-induced β cell pyroptosis. Besides, we also explored the cell-cell interactions from β cells to immune cells (Figure S6H).

β cell death constitutes a pathophysiological cornerstone in the natural progression of diabetes. Previous investigations into β cell death have primarily centered on apoptosis, necrosis, and autophagy. In this study, we uncovered a previously unknown mechanism in which proinflammatory macrophages induce β cell pyroptosis. An expanding body of research has linked β cell pyroptosis to diabetes.[91,92] These findings suggest that macrophage-mediated β cell pyroptosis may contribute to the increased incidence of diabetes among COVID-19 patients.

Limitations of the study

In this study, we analyzed the pancreatic autopsy samples from control and COVID19 subjects. COVID-19 subjects might have experienced bed resting and starvation (intensive care unit, ICU), which could have influenced the β cell phenotype, including INS content. Additionally, the inherent variability/heterogeneity of studying pancreatic autopsy samples could pose analytical challenges in distinguishing genuine disease pathology or differences between human donors from experimental noise.[93] The modest sample size and potential confounders in the clinical samples could also be limitations in this study. In VMI organoids, we found that some endothelial cells can form

(B) Dot plot showed the differential signaling from macrophages to β cells in human islets exposed to mock or CVB4 virus (2×10^6 PFU/mL).
(C) Dot plot of the expression level of *TNFSF12* in macrophage of human islets exposed to mock or SARS-CoV-2 virus (MOI = 1).
(D and E) Confocal images (D) and quantification (E) of CASP1 expression in INS$^+$ cells in control or 10 ng/mL TNFSF12-treated human islets. Scale bars, 25 μm.
(F and G) Confocal images (F) and quantification (G) of CASP1 expression in INS$^+$ cells in control or 10 ng/mL TNFSF12-treated VMI organoids at day 7 after reaggregation. Scale bars, 50 μm.
(H and I) Confocal images (H) and quantification (I) of CASP1 expression in INS$^+$ cells of SARS-CoV-2-exposed (MOI = 0.5) or CVB4-exposed (2×10^6 PFU/mL) human islets treated with control, 10 μg/mL TNFSF12 blocking antibody, 5 μg/mL IL-1β blocking antibody, or 10 μg/mL TNFSF12 + 5 μg/mL IL-1β blocking antibodies. Scale bars, 25 μm.
(J and K) Confocal images (J) and quantification (K) of CASP1 expression in INS$^+$ cells of VMI organoids containing proinflammatory macrophages at day 7 after reaggregation and treated with control, 10 μg/mL TNFSF12 blocking antibody, 5 μg/mL IL-1β blocking antibody, or 10 μg/mL TNFSF12 + 5 μg/mL IL-1β blocking antibodies. Scale bars, 50 μm.
(L and M) Confocal images (L) and quantification (M) of the CASP1 expression in INS$^+$ cells of pancreas autopsy samples from control ($N = 3$) and COVID-19 ($N = 4$) subjects. The insert shows a high magnification of cells. Scale bars, 50 μm.
$n = 3$ independent biological replicates. Data were presented as mean ± STDEV. p values were calculated by unpaired two-tailed Student's t test or one-way ANOVA with a common control. n.s., no significance; *$p < 0.05$, **$p < 0.01$, ***$p < 0.001$.
See also Figure S6.

small vessels. However, they cannot form intact blood vessels, which are likely required for monocyte infiltration into tissue. The vascular structure of VMI organoids is not fully functional yet, suggesting a need for further modification of the culture conditions. This might also be why we did not find obvious infiltration of monocytes into organoids containing endocrine cells and endothelial cells. Using this VMI organoid model, we observed that proinflammatory macrophage activation induced β cell death. However, we cannot distinguish whether the observed effects were derived from monocyte-derived or tissue-resident macrophages.

RESOURCE AVAILABILITY

Lead contact

Further information and requests for resources, reagents or codes should be directed to and will be fulfilled by the lead contact, Shuibing Chen (shc2034@med.cornell.edu).

Materials availability

This study did not generate new unique reagents.

Data and code availability

scRNA-seq, snATAC-seq, and RNA-seq data have been deposited at GEO: GSE247809 and are publicly available as of the date of publication. Accession numbers are listed in the key resources table. All original code has been deposited at Github and is publicly available as of the date of publication. DOI: https://doi.org/10.5281/zenodo.13231249 is listed in the key resources table. Any additional information required to reanalyze the data reported in this paper is available from the lead contact upon request.

ACKNOWLEDGMENTS

This work was supported by the National Institute of Diabetes, Digestive, and Kidney Diseases (NIDDK; R01DK137517, R01 DK124463, and 1R01DK 130454, S.C.), Bill and Melinda Gates foundation (INV-037420, S.C.), Department of Surgery, Weill Cornell Medicine (T.E. and S.C.), and R01DK121072 Department of Medicine, Weill Cornell Medicine (R.E.S.). In addition, S.C. and R.E.S. were supported as Irma Hirschl Trust Research Award Scholars. Human islets were received from the University of Pennsylvania human islet center with funding provided by the NIDDK-supported Human Pancreas Analysis Program (HPAP) (https://hpap.pmacs.upenn.edu/citation) grant UC4 DK112217 to A.N. Integrated Islet Isolation and Distribution Program (IIDP) was funded by NIH grant UC4DK098085. The authors would like to thank Didier Hober for kindly providing CVB4 virus. The authors would like to thank the Electron Microscopy & Histology services of the Weill Cornell Medicine Microscopy & Image Analysis Core and funds from an NIH Shared Instrumentation Grant for Shared Resources (S10RR027699). The authors also thank Dr. Mike Erdos from National Human Genome Research Institute for his help on islet collection.

AUTHOR CONTRIBUTIONS

S.C., R.E.S., L.Y., J.Q., S.C.J.P., and T.E. conceived and designed the experiments. L.Y., Y.H., T.Z., X.D. T.L., J.J.V., N.d.S, and A.R. performed cell differentiation and immunostaining. C.P., Y.S., and J.Z.X. assisted with the library preparation. J.G. performed SARS-CoV-2 infections. C.C.R provided feedback on bioinformatic analyses and manuscript writing. C.L., W.W., and A.N. prepared the human islets and human pancreas samples. R.E.S. provided the autopsy sample of COVID-19 patients. T.Z. and J.Z. performed the bioinformatics analyses.

DECLARATION OF INTERESTS

R.E.S. is on the scientific advisory board of Miromatrix Inc. and Lime Therapeutics and is a consultant and speaker for Alnylam Inc. S.C. and T.E are the co-founders of OncoBeat, LLC. S.C. is a consultant of Vesalius Therapeutics and co-founder of iOrganBio.

STAR★METHODS

Detailed methods are provided in the online version of this paper and include the following:

- KEY RESOURCES TABLE
- EXPERIMENTAL MODEL AND STUDY PARTICIPANT DETAILS
  - Human subjects
  - Sample size estimation
  - How subjects/samples were allocated to experimental groups
  - Virus strains
  - Cell Lines
- METHOD DETAILS
  - hPSC maintenance and pancreatic differentiation
  - hPSC differentiation toward endothelial cells
  - hPSCs differentiation towards macrophages
  - GeoMx transcriptomic and protein assays
  - Construction of VMI organoids
  - SARS-CoV-2 infection
  - CVB4 infection
  - Immunohistochemistry
  - Single-cell RNA-seq data analysis of human islets upon SARS-CoV-2 or CVB4 infection
  - Single-cell RNA-seq data analysis of VMI organoids
  - Single-nuclear ATAC-seq data analysis of VMI organoids
  - Bulk RNA-seq data analysis
  - RNA-Seq
- QUANTIFICATION AND STATISTICAL ANALYSIS

SUPPLEMENTAL INFORMATION

Supplemental information can be found online at https://doi.org/10.1016/j.stem.2024.08.007.

Received: November 17, 2023
Revised: June 5, 2024
Accepted: August 9, 2024
Published: September 3, 2024

REFERENCES

1. Hollstein, T., Schulte, D.M., Schulz, J., Glück, A., Ziegler, A.G., Bonifacio, E., Wendorff, M., Franke, A., Schreiber, S., Bornstein, S.R., and Laudes, M. (2020). Autoantibody-negative insulin-dependent diabetes mellitus after SARS-CoV-2 infection: a case report. Nat. Metab. *2*, 1021–1024. https://doi.org/10.1038/s42255-020-00281-8.
2. Soliman, A.T., Al-Amri, M., Alleethy, K., Alaaraj, N., Hamed, N., and De Sanctis, V. (2020). Newly-onset type 1 diabetes mellitus precipitated by COVID-19 in an 8-month-old infant. ahead of print. Acta Biomed. *91*. https://doi.org/10.23750/abm.v91i3.10074.
3. Heaney, A.I., Griffin, G.D., and Simon, E.L. (2020). Newly diagnosed diabetes and diabetic ketoacidosis precipitated by COVID-19 infection. Am. J. Emerg. Med. *38*, 2491.e3–2491.e4. https://doi.org/10.1016/j.ajem.2020.05.114.
4. Unsworth, R., Wallace, S., Oliver, N.S., Yeung, S., Kshirsagar, A., Naidu, H., Kwong, R.M.W., Kumar, P., and Logan, K.M. (2020). New-Onset Type 1 Diabetes in Children During COVID-19: Multicenter Regional Findings in the U.K. Diabetes Care *43*, e170–e171. https://doi.org/10.2337/dc20-1551.
5. Chee, Y.J., Ng, S.J.H., and Yeoh, E. (2020). Diabetic ketoacidosis precipitated by Covid-19 in a patient with newly diagnosed diabetes mellitus. Diabetes Res. Clin. Pract. *164*, 108166. https://doi.org/10.1016/j.diabres.2020.108166.

6. Vlad, A., Serban, V., Timar, R., Sima, A., Botea, V., Albai, O., Timar, B., and Vlad, M. (2021). Increased Incidence of Type 1 Diabetes during the COVID-19 Pandemic in Romanian Children. Medicina (Kaunas) *57*, 973. https://doi.org/10.3390/medicina57090973.
7. Rahmati, M., Keshvari, M., Mirnasuri, S., Yon, D.K., Lee, S.W., Il Shin, J., and Smith, L. (2022). The global impact of COVID-19 pandemic on the incidence of pediatric new-onset type 1 diabetes and ketoacidosis: A systematic review and meta-analysis. J. Med. Virol. *94*, 5112–5127. https://doi.org/10.1002/jmv.27996.
8. D'Souza, D., Empringham, J., Pechlivanoglou, P., Uleryk, E.M., Cohen, E., and Shulman, R. (2023). Incidence of Diabetes in Children and Adolescents During the COVID-19 Pandemic: A Systematic Review and Meta-Analysis. JAMA Netw. Open *6*, e2321281. https://doi.org/10.1001/jamanetworkopen.2023.21281.
9. Mefford, M.T., Wei, R., Lustigova, E., Martin, J.P., and Reynolds, K. (2023). Incidence of Diabetes Among Youth Before and During the COVID-19 Pandemic. JAMA Netw. Open *6*, e2334953. https://doi.org/10.1001/jamanetworkopen.2023.34953.
10. Vehik, K., Lynch, K.F., Wong, M.C., Tian, X., Ross, M.C., Gibbs, R.A., Ajami, N.J., Petrosino, J.F., Rewers, M., Toppari, J., et al. (2019). Prospective virome analyses in young children at increased genetic risk for type 1 diabetes. Nat. Med. *25*, 1865–1872. https://doi.org/10.1038/s41591-019-0667-0.
11. Krogvold, L., Edwin, B., Buanes, T., Frisk, G., Skog, O., Anagandula, M., Korsgren, O., Undlien, D., Eike, M.C., Richardson, S.J., et al. (2015). Detection of a low-grade enteroviral infection in the islets of langerhans of living patients newly diagnosed with type 1 diabetes. Diabetes *64*, 1682–1687. https://doi.org/10.2337/db14-1370.
12. Hyöty, H., Hiltunen, M., Knip, M., Laakkonen, M., Vähäsalo, P., Karjalainen, J., Koskela, P., Roivainen, M., Leinikki, P., and Hovi, T. (1995). A prospective study of the role of coxsackie B and other enterovirus infections in the pathogenesis of IDDM. Childhood Diabetes in Finland (DiMe) Study Group. Diabetes *44*, 652–657. https://doi.org/10.2337/diab.44.6.652.
13. Anagandula, M., Richardson, S.J., Oberste, M.S., Sioofy-Khojine, A.B., Hyöty, H., Morgan, N.G., Korsgren, O., and Frisk, G. (2014). Infection of human islets of Langerhans with two strains of Coxsackie B virus serotype 1: assessment of virus replication, degree of cell death and induction of genes involved in the innate immunity pathway. J. Med. Virol. *86*, 1402–1411. https://doi.org/10.1002/jmv.23835.
14. Honeyman, M.C., Coulson, B.S., Stone, N.L., Gellert, S.A., Goldwater, P.N., Steele, C.E., Couper, J.J., Tait, B.D., Colman, P.G., and Harrison, L.C. (2000). Association between rotavirus infection and pancreatic islet autoimmunity in children at risk of developing type 1 diabetes. Diabetes *49*, 1319–1324. https://doi.org/10.2337/diabetes.49.8.1319.
15. Hyöty, H., Leinikki, P., Reunanen, A., Ilonen, J., Surcel, H.M., Rilva, A., Käär, M.L., Huupponen, T., Hakulinen, A., and Mäkelä, A.L. (1988). Mumps infections in the etiology of type 1 (insulin-dependent) diabetes. Diabetes Res. *9*, 111–116.
16. Wang, X., Chen, J., Cao, Z., and Yu, X. (2023). Associations between human cytomegalovirus infection and type 2 diabetes mellitus: a systematic review and meta-analysis. BMJ Open *13*, e071934. https://doi.org/10.1136/bmjopen-2023-071934.
17. Bernard, H., Teijeiro, A., Chaves-Pérez, A., Perna, C., Satish, B., Novials, A., Wang, J.P., and Djouder, N. (2020). Coxsackievirus B Type 4 Infection in beta Cells Downregulates the Chaperone Prefoldin URI to Induce a MODY4-like Diabetes via Pdx1 Silencing. Cell Rep. Med. *1*, 100125. https://doi.org/10.1016/j.xcrm.2020.100125.
18. Tang, X., Uhl, S., Zhang, T., Xue, D., Li, B., Vandana, J.J., Acklin, J.A., Bonnycastle, L.L., Narisu, N., Erdos, M.R., et al. (2021). SARS-CoV-2 infection induces beta cell transdifferentiation. Cell Metab. *33*, 1577–1591.e7. https://doi.org/10.1016/j.cmet.2021.05.015.
19. Müller, J.A., Groß, R., Conzelmann, C., Krüger, J., Merle, U., Steinhart, J., Weil, T., Koepke, L., Bozzo, C.P., Read, C., et al. (2021). SARS-CoV-2 infects and replicates in cells of the human endocrine and exocrine pancreas. Nat. Metab. *3*, 149–165. https://doi.org/10.1038/s42255-021-00347-1.
20. Wu, C.T., Lidsky, P.V., Xiao, Y., Lee, I.T., Cheng, R., Nakayama, T., Jiang, S., Demeter, J., Bevacqua, R.J., Chang, C.A., et al. (2021). SARS-CoV-2 infects human pancreatic β cells and elicits β cell impairment. Cell Metab. *33*, 1565–1576.e5. https://doi.org/10.1016/j.cmet.2021.05.013.
21. Bain, C.C., Lucas, C.D., and Rossi, A.G. (2022). Pulmonary macrophages and SARS-Cov2 infection. Int. Rev. Cell Mol. Biol. *367*, 1–28. https://doi.org/10.1016/bs.ircmb.2022.01.001.
22. Yang, L., Han, Y., Jaffré, F., Nilsson-Payant, B.E., Bram, Y., Wang, P., Zhu, J., Zhang, T., Redmond, D., Houghton, S., et al. (2021). An Immuno-Cardiac Model for Macrophage-Mediated Inflammation in COVID-19 Hearts. Circ. Res. *129*, 33–46. https://doi.org/10.1161/CIRCRESAHA.121.319060.
23. Yamanaka, S. (2020). Pluripotent Stem Cell-Based Cell Therapy-Promise and Challenges. Cell Stem Cell *27*, 523–531. https://doi.org/10.1016/j.stem.2020.09.014.
24. Dutta, D., Heo, I., and Clevers, H. (2017). Disease Modeling in Stem Cell-Derived 3D Organoid Systems. Trends Mol. Med. *23*, 393–410. https://doi.org/10.1016/j.molmed.2017.02.007.
25. Kolios, G., and Moodley, Y. (2013). Introduction to stem cells and regenerative medicine. Respiration *85*, 3–10. https://doi.org/10.1159/000345615.
26. Wang, X. (2019). Stem cells in tissues, organoids, and cancers. Cell. Mol. Life Sci. *76*, 4043–4070. https://doi.org/10.1007/s00018-019-03199-x.
27. Yang, L., Han, Y., Nilsson-Payant, B.E., Gupta, V., Wang, P., Duan, X., Tang, X., Zhu, J., Zhao, Z., Jaffré, F., et al. (2020). A Human Pluripotent Stem Cell-based Platform to Study SARS-CoV-2 Tropism and Model Virus Infection in Human Cells and Organoids. Cell Stem Cell *27*, 125–136.e7. https://doi.org/10.1016/j.stem.2020.06.015.
28. Hollingsworth, J.W., Li, Z., Brass, D.M., Garantziotis, S., Timberlake, S.H., Kim, A., Hossain, I., Savani, R.C., and Schwartz, D.A. (2007). CD44 regulates macrophage recruitment to the lung in lipopolysaccharide-induced airway disease. Am. J. Respir. Cell Mol. Biol. *37*, 248–253. https://doi.org/10.1165/rcmb.2006-0363OC.
29. Qadri, M., Almadani, S., Jay, G.D., and Elsaid, K.A. (2018). Role of CD44 in Regulating TLR2 Activation of Human Macrophages and Downstream Expression of Proinflammatory Cytokines. J. Immunol. *200*, 758–767. https://doi.org/10.4049/jimmunol.1700713.
30. Etzerodt, A., and Moestrup, S.K. (2013). CD163 and inflammation: biological, diagnostic, and therapeutic aspects. Antioxid. Redox Signal. *18*, 2352–2363. https://doi.org/10.1089/ars.2012.4834.
31. De Vito, R., Alisi, A., Masotti, A., Ceccarelli, S., Panera, N., Citti, A., Salata, M., Valenti, L., Feldstein, A.E., and Nobili, V. (2012). Markers of activated inflammatory cells correlate with severity of liver damage in children with nonalcoholic fatty liver disease. Int. J. Mol. Med. *30*, 49–56. https://doi.org/10.3892/ijmm.2012.965.
32. Semnani-Azad, Z., Blanco Mejia, S., Connelly, P.W., Bazinet, R.P., Retnakaran, R., Jenkins, D.J.A., Harris, S.B., and Hanley, A.J. (2021). The association of soluble CD163, a novel biomarker of macrophage activation, with type 2 diabetes mellitus and its underlying physiological disorders: A systematic review. Obes. Rev. *22*, e13257. https://doi.org/10.1111/obr.13257.
33. Huang, A.L., and Vita, J.A. (2006). Effects of systemic inflammation on endothelium-dependent vasodilation. Trends Cardiovasc. Med. *16*, 15–20. https://doi.org/10.1016/j.tcm.2005.10.002.
34. Rose-John, S. (2017). The Soluble Interleukin 6 Receptor: Advanced Therapeutic Options in Inflammation. Clin. Pharmacol. Ther. *102*, 591–598. https://doi.org/10.1002/cpt.782.
35. Lammert, E., Cleaver, O., and Melton, D. (2001). Induction of pancreatic differentiation by signals from blood vessels. Science *294*, 564–567. https://doi.org/10.1126/science.1064344.

CellPress
OPEN ACCESS

36. Lammert, E., Cleaver, O., and Melton, D. (2003). Role of endothelial cells in early pancreas and liver development. Mech. Dev. *120*, 59–64. https://doi.org/10.1016/s0925-4773(02)00332-5.

37. Ranjan, A.K., Joglekar, M.V., and Hardikar, A.A. (2009). Endothelial cells in pancreatic islet development and function. Islets *1*, 2–9. https://doi.org/10.4161/isl.1.1.9054.

38. Oh, S.Y., Kim, J.Y., and Park, C. (2015). The ETS Factor, ETV2: a Master Regulator for Vascular Endothelial Cell Development. Mol. Cells *38*, 1029–1036. https://doi.org/10.14348/molcells.2015.0331.

39. Zhang, H., Yamaguchi, T., Kokubu, Y., and Kawabata, K. (2022). Transient ETV2 Expression Promotes the Generation of Mature Endothelial Cells from Human Pluripotent Stem Cells. Biol. Pharm. Bull. *45*, 483–490. https://doi.org/10.1248/bpb.b21-00929.

40. Elcheva, I., Brok-Volchanskaya, V., Kumar, A., Liu, P., Lee, J.H., Tong, L., Vodyanik, M., Swanson, S., Stewart, R., Kyba, M., et al. (2014). Direct induction of haematoendothelial programs in human pluripotent stem cells by transcriptional regulators. Nat. Commun. *5*, 4372. https://doi.org/10.1038/ncomms5372.

41. Morita, R., Suzuki, M., Kasahara, H., Shimizu, N., Shichita, T., Sekiya, T., Kimura, A., Sasaki, K., Yasukawa, H., and Yoshimura, A. (2015). ETS transcription factor ETV2 directly converts human fibroblasts into functional endothelial cells. Proc. Natl. Acad. Sci. USA *112*, 160–165. https://doi.org/10.1073/pnas.1413234112.

42. Hogan, M.F., and Hull, R.L. (2017). The islet endothelial cell: a novel contributor to beta cell secretory dysfunction in diabetes. Diabetologia *60*, 952–959. https://doi.org/10.1007/s00125-017-4272-9.

43. Voyta, J.C., Via, D.P., Butterfield, C.E., and Zetter, B.R. (1984). Identification and isolation of endothelial cells based on their increased uptake of acetylated-low density lipoprotein. J. Cell Biol. *99*, 2034–2040. https://doi.org/10.1083/jcb.99.6.2034.

44. Shin, J.H., Seo, B.G., Lee, I.W., Kim, H.J., Seo, E.C., Lee, K.M., Jeon, S.B., Baek, S.K., Kim, T.S., Lee, J.H., et al. (2022). Functional Characterization of Endothelial Cells Differentiated from Porcine Epiblast Stem Cells. Cells *11*, 1524. https://doi.org/10.3390/cells11091524.

45. Cusanovich, D.A., Hill, A.J., Aghamirzaie, D., Daza, R.M., Pliner, H.A., Berletch, J.B., Filippova, G.N., Huang, X., Christiansen, L., DeWitt, W.S., et al. (2018). A Single-Cell Atlas of In Vivo Mammalian Chromatin Accessibility. Cell *174*, 1309–1324.e18. https://doi.org/10.1016/j.cell.2018.06.052.

46. Krentz, N.A.J., Lee, M.Y.Y., Xu, E.E., Sproul, S.L.J., Maslova, A., Sasaki, S., and Lynn, F.C. (2018). Single-Cell Transcriptome Profiling of Mouse and hESC-Derived Pancreatic Progenitors. Stem Cell Rep. *11*, 1551–1564. https://doi.org/10.1016/j.stemcr.2018.11.008.

47. Zhu, H., Wang, G., Nguyen-Ngoc, K.V., Kim, D., Miller, M., Goss, G., Kovsky, J., Harrington, A.R., Saunders, D.C., Hopkirk, A.L., et al. (2023). Understanding cell fate acquisition in stem-cell-derived pancreatic islets using single-cell multiome-inferred regulomes. Dev. Cell *58*, 727–743.e11. https://doi.org/10.1016/j.devcel.2023.03.011.

48. Sharon, N., Vanderhooft, J., Straubhaar, J., Mueller, J., Chawla, R., Zhou, Q., Engquist, E.N., Trapnell, C., Gifford, D.K., and Melton, D.A. (2019). Wnt Signaling Separates the Progenitor and Endocrine Compartments during Pancreas Development. Cell Rep. *27*, 2281–2291.e5. https://doi.org/10.1016/j.celrep.2019.04.083.

49. Kim, C.Y., Lee, S.H., Jeon, H.M., Kim, H.K., Kang, C.M., and Lee, W.J. (2014). AFP-producing acinar cell carcinoma treated by pancreaticoduodenectomy in a patient with a previous radical subtotal gastrectomy by gastric cancer. Korean J. Hepatobiliary Pancreat. Surg. *18*, 33–37. https://doi.org/10.14701/kjhbps.2014.18.1.33.

50. Horn, S., Kirkegaard, J.S., Hoelper, S., Seymour, P.A., Rescan, C., Nielsen, J.H., Madsen, O.D., Jensen, J.N., Krüger, M., Grønborg, M., and Ahnfelt-Ronne, J. (2016). Research Resource: A Dual Proteomic Approach Identifies Regulated Islet Proteins During beta-Cell Mass Expansion In Vivo. Mol. Endocrinol. *30*, 133–143. https://doi.org/10.1210/me.2015-1208.

51. Bevacqua, R.J., Lam, J.Y., Peiris, H., Whitener, R.L., Kim, S., Gu, X., Friedlander, M.S.H., and Kim, S.K. (2021). SIX2 and SIX3 coordinately regulate functional maturity and fate of human pancreatic beta cells. Genes Dev. *35*, 234–249. https://doi.org/10.1101/gad.342378.120.

52. Kahn, S.E., Chen, Y.C., Esser, N., Taylor, A.J., van Raalte, D.H., Zraika, S., and Verchere, C.B. (2021). The beta Cell in Diabetes: Integrating Biomarkers With Functional Measures. Endocr. Rev. *42*, 528–583. https://doi.org/10.1210/endrev/bnab021.

53. Berger, C., and Zdzieblo, D. (2020). Glucose transporters in pancreatic islets. Pflugers Arch. *472*, 1249–1272. https://doi.org/10.1007/s00424-020-02383-4.

54. Thomsen, S.K., and Gloyn, A.L. (2014). The pancreatic beta cell: recent insights from human genetics. Trends Endocrinol. Metab. *25*, 425–434. https://doi.org/10.1016/j.tem.2014.05.001.

55. Kolic, J., Spigelman, A.F., Plummer, G., Leung, E., Hajmrle, C., Kin, T., Shapiro, A.M., Manning Fox, J.E., and MacDonald, P.E. (2013). Distinct and opposing roles for the phosphatidylinositol 3-OH kinase catalytic subunits p110alpha and p110beta in the regulation of insulin secretion from rodent and human beta cells. Diabetologia *56*, 1339–1349. https://doi.org/10.1007/s00125-013-2882-4.

56. Jonsson, A., Hedin, A., Müller, M., Skog, O., and Korsgren, O. (2020). Transcriptional profiles of human islet and exocrine endothelial cells in subjects with or without impaired glucose metabolism. Sci. Rep. *10*, 22315. https://doi.org/10.1038/s41598-020-79313-y.

57. Lindblom, P., Gerhardt, H., Liebner, S., Abramsson, A., Enge, M., Hellstrom, M., Backstrom, G., Fredriksson, S., Landegren, U., Nystrom, H.C., et al. (2003). Endothelial PDGF-B retention is required for proper investment of pericytes in the microvessel wall. Genes Dev. *17*, 1835–1840. https://doi.org/10.1101/gad.266803.

58. Mazier, W., and Cota, D. (2017). Islet Endothelial Cell: Friend and Foe. Endocrinology *158*, 226–228. https://doi.org/10.1210/en.2016-1925.

59. Gaengel, K., Niaudet, C., Hagikura, K., Laviña, B., Muhl, L., Hofmann, J.J., Ebarasi, L., Nyström, S., Rymo, S., Chen, L.L., et al. (2012). The sphingosine-1-phosphate receptor S1PR1 restricts sprouting angiogenesis by regulating the interplay between VE-cadherin and VEGFR2. Dev. Cell *23*, 587–599. https://doi.org/10.1016/j.devcel.2012.08.005.

60. Scholz, B., Korn, C., Wojtarowicz, J., Mogler, C., Augustin, I., Boutros, M., Niehrs, C., and Augustin, H.G. (2016). Endothelial RSPO3 Controls Vascular Stability and Pruning through Non-canonical WNT/Ca(2+)/NFAT Signaling. Dev. Cell *36*, 79–93. https://doi.org/10.1016/j.devcel.2015.12.015.

61. Modi, H., Jacovetti, C., Tarussio, D., Metref, S., Madsen, O.D., Zhang, F.P., Rantakari, P., Poutanen, M., Nef, S., Gorman, T., et al. (2015). Autocrine Action of IGF2 Regulates Adult beta-Cell Mass and Function. Diabetes *64*, 4148–4157. https://doi.org/10.2337/db14-1735.

62. D’Addio, F., Maestroni, A., Assi, E., Ben Nasr, M., Amabile, G., Usuelli, V., Loretelli, C., Bertuzzi, F., Antonioli, B., Cardarelli, F., et al. (2022). The IGFBP3/TMEM219 pathway regulates beta cell homeostasis. Nat. Commun. *13*, 684. https://doi.org/10.1038/s41467-022-28360-2.

63. McCulloch, L.J., van de Bunt, M., Braun, M., Frayn, K.N., Clark, A., and Gloyn, A.L. (2011). GLUT2 (SLC2A2) is not the principal glucose transporter in human pancreatic beta cells: implications for understanding genetic association signals at this locus. Mol. Genet. Metab. *104*, 648–653. https://doi.org/10.1016/j.ymgme.2011.08.026.

64. Li, J., Feng, Z.C., Yeung, F.S.H., Wong, M.R.M., Oakie, A., Fellows, G.F., Goodyer, C.G., Hess, D.A., and Wang, R. (2014). Aldehyde dehydrogenase 1 activity in the developing human pancreas modulates retinoic acid signalling in mediating islet differentiation and survival. Diabetologia *57*, 754–764. https://doi.org/10.1007/s00125-013-3147-y.

65. Moffitt, R.A., Marayati, R., Flate, E.L., Volmar, K.E., Loeza, S.G.H., Hoadley, K.A., Rashid, N.U., Williams, L.A., Eaton, S.C., Chung, A.H., et al. (2015). Virtual microdissection identifies distinct tumor- and stroma-specific subtypes of pancreatic ductal adenocarcinoma. Nat. Genet. *47*, 1168–1178. https://doi.org/10.1038/ng.3398.

66. He, W.T., Wan, H., Hu, L., Chen, P., Wang, X., Huang, Z., Yang, Z.H., Zhong, C.Q., and Han, J. (2015). Gasdermin D is an executor of pyroptosis and required for interleukin-1beta secretion. Cell Res. *25*, 1285–1298. https://doi.org/10.1038/cr.2015.139.

67. Karmakar, M., Minns, M., Greenberg, E.N., Diaz-Aponte, J., Pestonjamasp, K., Johnson, J.L., Rathkey, J.K., Abbott, D.W., Wang, K., Shao, F., et al. (2020). N-GSDMD trafficking to neutrophil organelles facilitates IL-1beta release independently of plasma membrane pores and pyroptosis. Nat. Commun. *11*, 2212. https://doi.org/10.1038/s41467-020-16043-9.

68. Rendeiro, A.F., Ravichandran, H., Bram, Y., Chandar, V., Kim, J., Meydan, C., Park, J., Foox, J., Hether, T., Warren, S., et al. (2021). The spatial landscape of lung pathology during COVID-19 progression. Nature *593*, 564–569. https://doi.org/10.1038/s41586-021-03475-6.

69. Cross, A.R., de Andrea, C.E., Villalba-Esparza, M., Landecho, M.F., Cerundolo, L., Weeratunga, P., Etherington, R.E., Denney, L., Ogg, G., Ho, L.P., et al. (2023). Spatial transcriptomic characterization of COVID-19 pneumonitis identifies immune circuits related to tissue injury. JCI Insight *8*, e157837. https://doi.org/10.1172/jci.insight.157837.

70. Mothes, R., Pascual-Reguant, A., Koehler, R., Liebeskind, J., Liebheit, A., Bauherr, S., Philipsen, L., Dittmayer, C., Laue, M., von Manitius, R., et al. (2023). Distinct tissue niches direct lung immunopathology via CCL18 and CCL21 in severe COVID-19. Nat. Commun. *14*, 791. https://doi.org/10.1038/s41467-023-36333-2.

71. Pita-Juarez, Y., Karagkouni, D., Kalavros, N., Melms, J.C., Niezen, S., Delorey, T.M., Essene, A.L., Brook, O.R., Pant, D., Skelton-Badlani, D., et al. (2022). A single-nucleus and spatial transcriptomic atlas of the COVID-19 liver reveals topological, functional, and regenerative organ disruption in patients. Preprint at bioRxiv. https://doi.org/10.1101/2022.10.27.514070.

72. Kulasinghe, A., Liu, N., Tan, C.W., Monkman, J., Sinclair, J.E., Bhuva, D.D., Godbolt, D., Pan, L., Nam, A., Sadeghirad, H., et al. (2023). Transcriptomic profiling of cardiac tissues from SARS-CoV-2 patients identifies DNA damage. Immunology *168*, 403–419. https://doi.org/10.1111/imm.13577.

73. Barrozo, E.R., Seferovic, M.D., Castro, E.C.C., Major, A.M., Moorshead, D.N., Jochum, M.D., Rojas, R.F., Shope, C.D., and Aagaard, K.M. (2023). SARS-CoV-2 niches in human placenta revealed by spatial transcriptomics. Med. *4*, 612–634.e4. https://doi.org/10.1016/j.medj.2023.06.003.

74. Wendisch, D., Dietrich, O., Mari, T., von Stillfried, S., Ibarra, I.L., Mittermaier, M., Mache, C., Chua, R.L., Knoll, R., Timm, S., et al. (2021). SARS-CoV-2 infection triggers profibrotic macrophage responses and lung fibrosis. Cell *184*, 6243–6261.e27. https://doi.org/10.1016/j.cell.2021.11.033.

75. Qadir, M.M.F., Bhondeley, M., Beatty, W., Gaupp, D.D., Doyle-Meyers, L.A., Fischer, T., Bandyopadhyay, I., Blair, R.V., Bohm, R., Rappaport, J., et al. (2021). SARS-CoV-2 infection of the pancreas promotes thrombofibrosis and is associated with new-onset diabetes. JCI Insight *6*, e151551. https://doi.org/10.1172/jci.insight.151551.

76. Babon, J.A.B., DeNicola, M.E., Blodgett, D.M., Crèvecoeur, I., Buttrick, T.S., Maehr, R., Bottino, R., Naji, A., Kaddis, J., Elyaman, W., et al. (2016). Analysis of self-antigen specificity of islet-infiltrating T cells from human donors with type 1 diabetes. Nat. Med. *22*, 1482–1487. https://doi.org/10.1038/nm.4203.

77. Coppieters, K.T., Dotta, F., Amirian, N., Campbell, P.D., Kay, T.W.H., Atkinson, M.A., Roep, B.O., and von Herrath, M.G. (2012). Demonstration of islet-autoreactive CD8 T cells in insulitic lesions from recent onset and long-term type 1 diabetes patients. J. Exp. Med. *209*, 51–60. https://doi.org/10.1084/jem.20111187.

78. Brissova, M., Aamodt, K., Brahmachary, P., Prasad, N., Hong, J.Y., Dai, C., Mellati, M., Shostak, A., Poffenberger, G., Aramandla, R., et al. (2014). Islet microenvironment, modulated by vascular endothelial growth factor-A signaling, promotes beta cell regeneration. Cell Metab. *19*, 498–511. https://doi.org/10.1016/j.cmet.2014.02.001.

79. Burganova, G., Bridges, C., Thorn, P., and Landsman, L. (2021). The Role of Vascular Cells in Pancreatic Beta-Cell Function. Front. Endocrinol. (Lausanne) *12*, 667170. https://doi.org/10.3389/fendo.2021.667170.

80. Nikolova, G., Jabs, N., Konstantinova, I., Domogatskaya, A., Tryggvason, K., Sorokin, L., Fässler, R., Gu, G., Gerber, H.P., Ferrara, N., et al. (2006). The vascular basement membrane: a niche for insulin gene expression and Beta cell proliferation. Dev. Cell *10*, 397–405. https://doi.org/10.1016/j.devcel.2006.01.015.

81. Banaei-Bouchareb, L., Gouon-Evans, V., Samara-Boustani, D., Castellotti, M.C., Czernichow, P., Pollard, J.W., and Polak, M. (2004). Insulin cell mass is altered in Csf1op/Csf1op macrophage-deficient mice. J. Leukoc. Biol. *76*, 359–367. https://doi.org/10.1189/jlb.1103591.

82. Cosentino, C., and Regazzi, R. (2021). Crosstalk between Macrophages and Pancreatic beta-Cells in Islet Development, Homeostasis and Disease. Int. J. Mol. Sci. *22*, 1765. https://doi.org/10.3390/ijms22041765.

83. Liao, M., Liao, J., Qu, J., Shi, P., Cheng, Y., Pan, Q., Zhao, N., Zhang, X., Zhang, L., Tan, Y., et al. (2023). Hepatic TNFRSF12A promotes bile acid-induced hepatocyte pyroptosis through NFkappaB/Caspase-1/GSDMD signaling in cholestasis. Cell Death Discov. *9*, 26. https://doi.org/10.1038/s41420-023-01326-z.

84. Burkly, L.C., Michaelson, J.S., and Zheng, T.S. (2011). TWEAK/Fn14 pathway: an immunological switch for shaping tissue responses. Immunol. Rev. *244*, 99–114. https://doi.org/10.1111/j.1600-065X.2011.01054.x.

85. Dohi, T., and Burkly, L.C. (2012). The TWEAK/Fn14 pathway as an aggravating and perpetuating factor in inflammatory diseases: focus on inflammatory bowel diseases. J. Leukoc. Biol. *92*, 265–279. https://doi.org/10.1189/jlb.0112042.

86. Cheng, E., Armstrong, C.L., Galisteo, R., and Winkles, J.A. (2013). TWEAK/Fn14 Axis-Targeted Therapeutics: Moving Basic Science Discoveries to the Clinic. Front. Immunol. *4*, 473. https://doi.org/10.3389/fimmu.2013.00473.

87. Lam, E.T., Eckhardt, S.G., Messersmith, W., Jimeno, A., O'Bryant, C.L., Ramanathan, R.K., Weiss, G.J., Chadha, M., Oey, A., Ding, H.T., et al. (2018). Phase I Study of Enavatuzumab, a First-in-Class Humanized Monoclonal Antibody Targeting the TWEAK Receptor, in Patients with Advanced Solid Tumors. Mol. Cancer Ther. *17*, 215–221. https://doi.org/10.1158/1535-7163.MCT-17-0330.

88. Lassen, U.N., Meulendijks, D., Siu, L.L., Karanikas, V., Mau-Sorensen, M., Schellens, J.H.M., Jonker, D.J., Hansen, A.R., Simcox, M.E., Schostack, K.J., et al. (2015). A phase I monotherapy study of RG7212, a first-in-class monoclonal antibody targeting TWEAK signaling in patients with advanced cancers. Clin. Cancer Res. *21*, 258–266. https://doi.org/10.1158/1078-0432.CCR-14-1334.

89. Michaelson, J.S., Kelly, R., Yang, L., Zhang, X., Wortham, K., and Joseph, I.B.J.K. (2012). The anti-Fn14 antibody BIIB036 inhibits tumor growth in xenografts and patient derived primary tumor models and enhances efficacy of chemotherapeutic agents in multiple xenograft models. Cancer Biol. Ther. *13*, 812–821. https://doi.org/10.4161/cbt.20564.

90. Wisniacki, N., Amaravadi, L., Galluppi, G.R., Zheng, T.S., Zhang, R., Kong, J., and Burkly, L.C. (2013). Safety, tolerability, pharmacokinetics, and pharmacodynamics of anti-TWEAK monoclonal antibody in patients with rheumatoid arthritis. Clin. Ther. *35*, 1137–1149. https://doi.org/10.1016/j.clinthera.2013.06.008.

91. Carlos, D., Costa, F.R.C., Pereira, C.A., Rocha, F.A., Yaochite, J.N.U., Oliveira, G.G., Carneiro, F.S., Tostes, R.C., Ramos, S.G., Zamboni, D.S., et al. (2017). Mitochondrial DNA Activates the NLRP3 Inflammasome and Predisposes to Type 1 Diabetes in Murine Model. Front. Immunol. *8*, 164. https://doi.org/10.3389/fimmu.2017.00164.

92. Kim, Y., Wang, W., Okla, M., Kang, I., Moreau, R., and Chung, S. (2016). Suppression of NLRP3 inflammasome by γ-tocotrienol ameliorates type 2 diabetes. J. Lipid Res. *57*, 66–76. https://doi.org/10.1194/jlr.M062828.

93. Gloyn, A.L., Ibberson, M., Marchetti, P., Powers, A.C., Rorsman, P., Sander, M., and Solimena, M. (2022). Every islet matters: improving the

CellPress
OPEN ACCESS

impact of human islet research. Nat. Metab. *4*, 970–977. https://doi.org/10.1038/s42255-022-00607-8.

94. Chehadeh, W., Kerr-Conte, J., Pattou, F., Alm, G., Lefebvre, J., Wattré, P., and Hober, D. (2000). Persistent infection of human pancreatic islets by coxsackievirus B is associated with alpha interferon synthesis in beta cells. J. Virol. *74*, 10153–10164. https://doi.org/10.1128/jvi.74.21.10153-10164.2000.

95. Ricordi, C., Goldstein, J.S., Balamurugan, A.N., Szot, G.L., Kin, T., Liu, C., Czarniecki, C.W., Barbaro, B., Bridges, N.D., Cano, J., et al. (2016). National Institutes of Health-Sponsored Clinical Islet Transplantation Consortium Phase 3 Trial: Manufacture of a Complex Cellular Product at Eight Processing Facilities. Diabetes *65*, 3418–3428. https://doi.org/10.2337/db16-0234.

96. Zeng, H., Guo, M., Zhou, T., Tan, L., Chong, C.N., Zhang, T., Dong, X., Xiang, J.Z., Yu, A.S., Yue, L., et al. (2016). An Isogenic Human ESC Platform for Functional Evaluation of Genome-wide-Association-Study-Identified Diabetes Genes and Drug Discovery. Cell Stem Cell *19*, 326–340. https://doi.org/10.1016/j.stem.2016.07.002.

97. Harding, A., Cortez-Toledo, E., Magner, N.L., Beegle, J.R., Coleal-Bergum, D.P., Hao, D., Wang, A., Nolta, J.A., and Zhou, P. (2017). Highly Efficient Differentiation of Endothelial Cells from Pluripotent Stem Cells Requires the MAPK and the PI3K Pathways. Stem Cells *35*, 909–919. https://doi.org/10.1002/stem.2577.

98. Cao, X., Yakala, G.K., van den Hil, F.E., Cochrane, A., Mummery, C.L., and Orlova, V.V. (2019). Differentiation and Functional Comparison of Monocytes and Macrophages from hiPSCs with Peripheral Blood Derivatives. Stem Cell Rep. *12*, 1282–1297. https://doi.org/10.1016/j.stemcr.2019.05.003.

99. Lun, A.T.L., Bach, K., and Marioni, J.C. (2016). Pooling across cells to normalize single-cell RNA sequencing data with many zero counts. Genome Biol. *17*, 75. https://doi.org/10.1186/s13059-016-0947-7.

100. Bernstein, N.J., Fong, N.L., Lam, I., Roy, M.A., Hendrickson, D.G., and Kelley, D.R. (2020). Solo: Doublet Identification in Single-Cell RNA-Seq via Semi-Supervised Deep Learning. Cell Syst. *11*, 95–101.e5. https://doi.org/10.1016/j.cels.2020.05.010.

101. Haghverdi, L., Lun, A.T.L., Morgan, M.D., and Marioni, J.C. (2018). Batch effects in single-cell RNA-sequencing data are corrected by matching mutual nearest neighbors. Nat. Biotechnol. *36*, 421–427. https://doi.org/10.1038/nbt.4091.

102. Wu, T., Hu, E., Xu, S., Chen, M., Guo, P., Dai, Z., Feng, T., Zhou, L., Tang, W., Zhan, L., et al. (2021). clusterProfiler 4.0: A universal enrichment tool for interpreting omics data. Innovation (Camb) *2*, 100141. https://doi.org/10.1016/j.xinn.2021.100141.

103. McGinnis, C.S., Murrow, L.M., and Gartner, Z.J. (2019). DoubletFinder: Doublet Detection in Single-Cell RNA Sequencing Data Using Artificial Nearest Neighbors. Cell Syst. *8*, 329–337.e4. https://doi.org/10.1016/j.cels.2019.03.003.

104. Hao, Y., Hao, S., Andersen-Nissen, E., Mauck, W.M., 3rd, Zheng, S., Butler, A., Lee, M.J., Wilk, A.J., Darby, C., Zager, M., et al. (2021). Integrated analysis of multimodal single-cell data. Cell *184*, 3573–3587.e29. https://doi.org/10.1016/j.cell.2021.04.048.

105. Jin, S., Guerrero-Juarez, C.F., Zhang, L., Chang, I., Ramos, R., Kuan, C.H., Myung, P., Plikus, M.V., and Nie, Q. (2021). Inference and analysis of cell-cell communication using CellChat. Nat. Commun. *12*, 1088. https://doi.org/10.1038/s41467-021-21246-9.

106. Stuart, T., Srivastava, A., Madad, S., Lareau, C.A., and Satija, R. (2021). Single-cell chromatin state analysis with Signac. Nat. Methods *18*, 1333–1341. https://doi.org/10.1038/s41592-021-01282-5.

107. Dobin, A., Davis, C.A., Schlesinger, F., Drenkow, J., Zaleski, C., Jha, S., Batut, P., Chaisson, M., and Gingeras, T.R. (2013). STAR: ultrafast universal RNA-seq aligner. Bioinformatics *29*, 15–21. https://doi.org/10.1093/bioinformatics/bts635.

108. Anders, S., Pyl, P.T., and Huber, W. (2015). HTSeq–a Python framework to work with high-throughput sequencing data. Bioinformatics *31*, 166–169. https://doi.org/10.1093/bioinformatics/btu638.

109. Love, M.I., Huber, W., and Anders, S. (2014). Moderated estimation of fold change and dispersion for RNA-seq data with DESeq2. Genome Biol. *15*, 550. https://doi.org/10.1186/s13059-014-0550-8.

110. Ziemann, M., Kaspi, A., and El-Osta, A. (2019). Digital expression explorer 2: a repository of uniformly processed RNA sequencing data. GigaScience *8*, giz022. https://doi.org/10.1093/gigascience/giz022.

111. Jiang, H., Lei, R., Ding, S.W., and Zhu, S. (2014). Skewer: a fast and accurate adapter trimmer for next-generation sequencing paired-end reads. BMC Bioinformatics *15*, 182. https://doi.org/10.1186/1471-2105-15-182.

112. Davis, M.P.A., van Dongen, S., Abreu-Goodger, C., Bartonicek, N., and Enright, A.J. (2013). Kraken: a set of tools for quality control and analysis of high-throughput sequence data. Methods *63*, 41–49. https://doi.org/10.1016/j.ymeth.2013.06.027.

113. Ramírez, F., Ryan, D.P., Grüning, B., Bhardwaj, V., Kilpert, F., Richter, A.S., Heyne, S., Dündar, F., and Manke, T. (2016). deepTools2: a next generation web server for deep-sequencing data analysis. Nucleic Acids Res. *44*, W160–W165. https://doi.org/10.1093/nar/gkw257.

114. Robinson, M.D., McCarthy, D.J., and Smyth, G.K. (2010). edgeR: a Bioconductor package for differential expression analysis of digital gene expression data. Bioinformatics *26*, 139–140. https://doi.org/10.1093/bioinformatics/btp616.

STAR★METHODS

KEY RESOURCES TABLE

REAGENT or RESOURCE	SOURCE	IDENTIFIER
Antibodies		
Polyclonal Guinea Pig Anti-Insulin	Dako	Cat #A0564; RRID: AB_10013624
Glucagon Rabbit Ab	Cell Signaling	Cat #2760; RRID: AB_659831
Polyclonal Rabbit Anti-Somatostatin	Dako	Cat #A0566; RRID: AB_2688022
Human CD31/PECAM-1 Antibody	R&D Systems	Cat #AF806; RRID: AB_355617
Purified anti-human CD68 Antibody	Biolegend	Cat # 333802; RRID: AB_1089058
Cleaved Caspase-1 (Asp297)	Cell Signaling	Cat # 4199; RRID: AB_1903916
hPDX-1 Affinity purified goat igG	R&D Systems	Cat # AF2419; RRID: AB_355257
Nkx6.1 (D8O4R) Rabbit mAb	Cell Signaling	Cat #54551; RRID: AB_2722625
APC anti-mouse/human CD11b Antibody	Biolegend	Cat #101212; RRID: AB_312795
APC anti-human CD206 (MMR) Antibody	Biolegend	Cat #321109; RRID: AB_571884
APC anti-human CD14	Biolegend	Cat #301808; RRID: AB_314190
Insulin Monoclonal Antibody (ICBTACLS), Alexa Fluor™ 488	Thermo Fisher Scientific	Cat # 53-9769-82; RRID: AB_2574469
Cytokeratin, pan Antibody (AE-1/AE-3) [DyLight 594]	Novus Biological	Cat # NBP2- 33200DL594; RRID: AB_2924199
caspase-1 Antibody (14F468)	Santa Cruz	Cat #sc-56036; RRID: AB_781816
Enterovirus (Concentrate)	Dako	Cat #M7064; RRID: RRID: AB_2118128
CD163 (D6U1J) Rabbit mAb	Cell Signaling	Cat #93498; RRID: AB_2800204
Anti-PRSS1 antibody produced in rabbit	Sigma Aldrich	Cat #HPA063471; RRID: AB_2685017
Purified anti-Cytokeratin 19	Biolegend	Cat #628502; RRID: AB_439773
Human B7-1/CD80 MAb (Clone 37711)	RnD	Cat # MAB140-100; RRID: AB_2244549
Alexa Fluor 488 AffiniPure Donkey Anti-Guinea Pig IgG (H+L)	Jackson Immunoresearch Labs	Cat #706-545-148; RRID: AB_2340472
Donkey anti-Mouse IgG (H+L) Highly Cross-Adsorbed Secondary Antibody, Alexa Fluor 594	Thermo Fisher Scientific	Cat #A-21203; RRID: AB_2535789
Donkey anti-Rabbit IgG (H+L) Secondary Antibody, Alexa Fluor 594 conjugate	Thermo Fisher Scientific	Cat #A-21207; RRID: AB_141637
Donkey anti-Rabbit IgG (H+L) Secondary Antibody, Alexa Fluor 647 conjugate	Thermo Fisher Scientific	Cat #A-31573; RRID: AB_2536183
Donkey anti-Mouse IgG (H+L) Secondary Antibody, Alexa Fluor 647	Thermo Fisher Scientific	Cat #A-31571; RRID: AB_162542
Donkey anti-Goat IgG (H+L) Cross-Adsorbed Secondary Antibody, Alexa Fluor 647	Thermo Fisher Scientific	Cat #A-21447; RRID: AB_2535864
Donkey anti-Sheep IgG (H+L) Cross-Adsorbed Secondary Antibody, Alexa Fluor 647	Thermo Fisher Scientific	Cat #A-21448; RRID: AB_2535865
Donkey anti-Mouse IgG (H+L) Highly Cross-Adsorbed Secondary Antibody, Alexa Fluor Plus 405	Thermo Fisher Scientific	Cat #A48257; RRID: AB_2884884
Bacterial and virus strains		
SARS-CoV-2: hCoV/USA/WA1/2020	WRCEVA (UTMB Health)	https://www.utmb.edu/wrceva/viruses
CVB4 E2	Didier Hober lab[94]	N/A

(Continued on next page)

Continued		
REAGENT or RESOURCE	SOURCE	IDENTIFIER
**Biological samples**		
Human samples and refer to Table S1 for all further details	N/A	N/A
**Chemicals, peptides, and recombinant proteins**		
Y-27632	MedchemExpress	Cat #HY-10583
CHIR99021	Cayman Chemica	Cat #13122
SANT-1	Sigma Aldrich	Cat #S4572-25MG
Retinoic acid	Sigma Aldrich	Cat #R2625-500MG
TPPB	Tocris Bioscience	Cat #5343
T3 hormone	Sigma Aldrich	Cat #T6397-100MG
Zinc sulfate heptahydrate	Sigma Aldrich	Cat #Z0251-100G
Heparin sodium salt from porcine intestinal mucosa	Sigma Aldrich	Cat #H3149-1MU
g-Secretase Inhibitor XX	Millipore	Cat #565789-1MG
ALK5 Inhibitor II	Cayman Chemical	Cat #14794
L-Ascorbic acid	Sigma Aldrich	Cat #A4544-100G
R428	MedchemExpress	Cat #HY-15150
N-acetyl-L-cysteine	Sigma Aldrich	Cat #A9165-5G
Trolox	Millipore	Cat #648471
LDN 193189 hydrochloride - DM 3189 hydrochloride	Axon Medchem	Cat #Axon 1509
Poly (vinyl alcohol)	Sigma Aldrich	Cat #P8136
L-Ascorbic acid 2-phosphate sesquimagnesium salt hydrate	Sigma Aldrich	Cat #A8960
SB431542	R&D Systems	Cat #1614/50
1-Thioglycerol	Sigma Aldrich	Cat #M6145
Activin A	N/A	N/A
Recombinant Human bFGF Protein	Peprotech	Cat #100-18B-500UG
Recombinant Human BMP-4 Protein	R & D Systems	Cat #314-BP
Recombinant Human VEGF Protein	R & D Systems	Cat #293-VE-500/CF
Recombinant Human SCF Protein	R & D Systems	Cat #7466-SC-100/CF
Recombinant Human IL-6 Protein	R & D Systems	Cat #206-IL-200/CF
Recombinant Human IL-3 Protein	R & D Systems	Cat #203-IL-050/CF
Recombinant Human TPO Protein	R & D Systems	Cat #288-TP-200/CF
Recombinant Human M-CSF Protein	R & D Systems	Cat #216-MC-025
**Critical commercial assays**		
LEGENDplex Human Inflammation Panel 1	Biolegend	Cat #740808
**Deposited data**		
scRNA-seq, snATAC-seq, RNA-seq	This study	GSE247809
Deposited custom code	This study	DOI: https://doi.org/10.5281/zenodo.13231249
**Experimental models: Cell lines**		
hESC line MEL-1	University of Queensland	0139
hESC line H1	Harvard University	0014
293T	ATCC	Cat #CRL-11268
hESC line H9	Harvard University	0022
Vero E6	ATCC	Cat # CRL-1586 RRID: CVCL_0574
**Oligonucleotides**		
Primers for real-time quantitative PCR and refer to Table S3 for all further details	N/A	N/A

(*Continued on next page*)

Continued		
REAGENT or RESOURCE	SOURCE	IDENTIFIER
Software and algorithms		
Rstudio	Rstudio	https://rstudio.com
Seurat R package v3.1.4	N/A	https://satijalab.org/seurat/
Dplyr package	LHRI	https://dplyr.tidyverse.org/
Adobe illustrator CC2017	Adobe	https://www.adobe.com/product/photoshop.html
Graphpad Prism 6	Graphpad software	https://www.graphpad.com
Other		
StemFlex	GIBCO Thermo Fisher	Cat #A3349401
MCDB 131 Medium, no glutamine	Thermo Fisher Scientific	Cat #10372019
STEMdiff APEL2 Medium	Stem Cell Technologies	Cat #05270
Endothelial Cell Growth Medium MV2 (Readyto-use)	PromoCell	Cat #C-22022
F12	GIBCO Thermo Fisher	Cat #31765035
Lipids	GIBCO Thermo Fisher	Cat #11905031
Penicillin-Streptomycin (5,000 U/mL)	GIBCO Thermo Fisher	Cat #15070063
MEM Non-Essential Amino Acids Solution (100X)	GIBCO Thermo Fisher	Cat #11140050
IMDM	GIBCO Thermo Fisher	Cat #21056023
GlutaMAX Supplement	Thermo Fisher Scientific	Cat #35050079
ITS-X	Thermo Fisher Scientific	Cat #51500056
Accutase	StemCell Technologies, Inc.	Cat # 07920
ReleSR	StemCell Technologies, Inc.	Cat # 05872
Matrigel	Corning	Cat #354234

EXPERIMENTAL MODEL AND STUDY PARTICIPANT DETAILS

Human subjects

Human pancreas

Pancreas tissues from COVID-19 samples were provided by the Weill Cornell Medicine Department of Pathology using protocols approved by the Tissue Procurement Facility of Weill Cornell Medicine. Experiments using samples from human subjects were conducted in accordance with local regulations and with the approval of the IRB at the Weill Cornell Medicine. The autopsy samples were collected under protocol 20-04021814. For GeoMx RNA and protein analysis, seven COVID19 human pancreas samples were deceased upon tissue acquisition and were provided from Weill Cornell Medicine as fixed samples. Eight control human pancreas samples were obtained from the Human Islet Core at the University of Pennsylvania. The pancreatic organs were obtained from the organ procurement organization under the United Network for Organ Sharing. The organs were kept in the University of Wisconsin solution at 4°C before the tissue samples biopsies. The freshly dissected tissues (<3mm thick) were fixed with 10% formalin for 8 hours at room temperature. The tissue samples were rinsed with running tap water for 5 min then through 80% and 95% alcohol for 1 hour each, followed with 2 rinses of 100% alcohol for 1 hour each for dehydration. The tissues were cleared in xylene 3 times for 1 hour each. The tissues were immersed in paraffin 3 times for 1 hour each before being embedded in a paraffin block. The paraffin-embedded tissue blocks were sectioned at 5 μm thickness on a microtome and floated in a 40°C water bath containing distilled water. The sections were transferred onto glass slides which were suitable for immunohistochemistry and the slides were dried at room temperature before use. See Table S1 for age, gender and more information.

Human islets

The pancreatic organs were obtained from the local organ procurement organization under the United Network for Organ Sharing (UNOS). The islets were isolated in the Human Islet Core at the University of Pennsylvania following the guidelines of Clinical Islet Transplantation consortium protocol.[95] Briefly, the pancreas was digested following intraductal injection of Collagenase & Neutral Protease in Hanks' balanced salt solution. Liberated islets were then purified on continuous density gradients (Cellgro/Mediatech) using the COBE 2991 centrifuge and cultured in CIT culture media and kept in a humidified 5% CO_2 incubator. See Table S1 for age, gender and more information.

Sample size estimation

For spatial multiomics analysis, 8 control human pancreas samples and 7 COVID-19 human pancreas samples were used. For scRNA-seq analysis of human islets, 4 islets samples were used for control or virus exposure. For assays using VMI organoids, three independent experiments were performed expect scRNA-seq and snATAC-seq.

How subjects/samples were allocated to experimental groups

The experiments using human pancreas and islets were carried out in a blinded manner. Briefly, the investigators performing experiments were blinded to the patient information. The human pancreas and islets samples were used upon available. For organoids with different treatments, all organoids from the same experiments were grown on the same batch and were randomly assigned to each experimental group.

Virus strains

SARS-CoV-2 Viruses

SARS-CoV-2, isolate USA-WA1/2020 was obtained from World Reference Center for Emerging Viruses and Arboviruses located at University of Texas Medical Branch via the CDC. Vero E6 cells (ATCC) served as the culture system for SARS-CoV-2 propagation, utilizing EMEM with a supplement of 10% FCS, 1 mM Sodium Pyruvate, and 10 mM HEPES (citation). All work involving live SARS-CoV-2 was performed in the CDC/USDA-approved BSL-3 facility at Aaron Diamond AIDS Research Center located at Columbia University. The Aaron Diamond AIDS Research Center's BSL-3 facility at Columbia University prepared the SARS-CoV-2 WA1 strain, subsequently stored at -70°C.

CVB4 Viruses

The aliquots of CVB4 E2, the diabetogenic strain of coxsackievirus B4 virus were provided by Didier Hober and were then stored frozen at −80°C.

Cell Lines

HEK293T (human [*Homo sapiens*] fetal kidney) and Vero E6 (African green monkey [*Chlorocebus aethiops*] kidney) were obtained from ATCC). Cells were cultured in Dulbecco's Modified Eagle Medium (DMEM) supplemented with 10% FBS and 100 I.U./mL penicillin and 100 μg/mL streptomycin. All cell lines were incubated at 37°C with 5% CO_2.

METHOD DETAILS

hPSC maintenance and pancreatic differentiation

INS$^{GFP/W}$ MEL-1 cells were used to generate pancreatic endocrine cells using a previously reported strategy.[96] In brief, *INS*$^{GFP/W}$ MEL-1 cells were cultured on Matrigel-coated 6-well plates in StemFlex medium (Gibco Thermo Fisher) and maintained at 37°C with 5% CO_2. At stage 1-day 1, cells were exposed to basal RPMI 1640 medium supplemented with 1× Glutamax (Thermo Fisher Scientific), 50 μg/mL Normocin, 100 ng/mL Activin A (R&D systems), and 3 μM of CHIR99021 (GSK3β inhibitor 3, Cayman Chemical) for 24 hours. At stage 1-day 2 and 3, the medium was changed to basal RPMI 1640 medium supplemented with 1× Glutamax, 50 μg/mL Normocin, 0.2% fetal bovine serum (FBS, Corning), 100 ng/mL Activin A for 2 days. At stage 2-day 4 and 5, the resulting definitive endoderm cells were cultured in MCDB131 medium (Thermo Fisher Scientific) supplemented with 1.5 g/L sodium bicarbonate, 1× Glutamax, 10 mM glucose (Sigma Aldrich) at final concentration, 2% bovine serum albumin (BSA, Lampire), 0.25 mM L-ascorbic acid (Sigma Aldrich) and 50 ng/ml of fibroblast growth factor 7 (FGF-7, Peprotech) to acquire primitive gut tube. At stage 3-day 6 and day 7, cells were induced to differentiate to posterior foregut in MCDB 131 medium supplemented with 2.5 g/L sodium bicarbonate, 1× Glutamax, 10 mM glucose at final concentration, 2% BSA, 0.25 mM L-ascorbic acid, 50 ng/ml of FGF-7, 1 μM Retinoic acid (RA; Sigma Aldrich), 100 nM LDN193189 (LDN, Axon Medchem), 1:200 ITS-X (Thermo Fisher Scientific), 200 nM TPPB (Tocris Bioscience) and 0.25 μM SANT-1 (Sigma Aldrich) for 2 days. At stage 4-day 8-day 10, cells were differentiated to pancreatic endoderm in MCDB 131 medium supplemented with 2.5 g/L sodium bicarbonate, 1× Glutamax, 10 mM glucose at final concentration, 2% BSA, 0.25 mM L-ascorbic acid, 2 ng/ml of FGF-7, 0.1 μM RA, 200 nM LDN193189, 1:200 ITS-X, 100 nM TPPB and 0.25 μM SANT-1 for 3 days. At stage 5-day 11-day 13, cells were differentiated to pancreatic endocrine precursors in MCDB 131 medium supplemented with 1.5 g/L sodium bicarbonate, 1× Glutamax, 20 mM glucose at final concentration, 2% BSA, 0.05 μM RA, 100 nM LDN, 1:200 ITS-X, 0.25 μM SANT-1, 1 mM T3 hormone (Sigma Aldrich), 10 μM ALK5 inhibitor II (Cayman Chemical), 10 μM zinc sulfate heptahydrate (Sigma Aldrich) and 10 μg/ml of heparin (Sigma Aldrich) for 3 days. At day 14, cells were exposed to MCDB 131 medium supplemented with 1.5 g/L sodium bicarbonate, 1× Glutamax, 20 mM glucose at final concentration, 2% BSA, 100 nM LDN193189, 1:200 ITS-X, 1 μM T3, 10 μM ALK5 inhibitor II, 10 μM zinc sulfate, 10 μg/ml of heparin, 100 nM gamma secretase inhibitor XX (Millipore) for 7 days. Then, cells were exposed to MCDB 131 medium supplemented with 1.5 g/L sodium bicarbonate, 1× Glutamax, 20 mM glucose at final concentration, 2% BSA, 1:200 ITS-X, 1 μM T3, 10 μM ALK5 inhibitor II, 10 μM zinc sulfate heptahydrate, 10 μg/ml of heparin, 1 mM N-acetyl cysteine (Sigma Aldrich), 10 μM Trolox (Millipore), 2 μM R428 (MedchemExpress) for another 7-15 days. The medium was subsequently refreshed every day.

hPSC differentiation toward endothelial cells

To derive endothelial cells from hPSCs, we optimized a previously reported strategy.[97] Briefly, H1 hESCs were passaged onto Matrigel-coated 6-well plates in StemFlex medium. Before differentiation, we infected H1 hESCs with lentivirus carrying ETV2. After two days selection with 1 μg/ml puromycin and 1 day recovery in StemFlex medium, hESCs will be switched to StemDiff APEL medium (STEMCELL Technologies) with 6 μM CHIR99021 for 2 days. Then, cells were cultured in StemDiff APEL medium with an additional of 25 ng/ml BMP-4, 10 ng/ml bFGF and 50 ng/ml VEGF (R&D Systems) for another two days. On day 4, cells were dissociated with Accutase (Innovative Cell Technologies) and reseeded onto p100 culture dishes in EC Growth Medium MV2 (Promocell) with an additional 50 ng/ml VEGF for 4-6 days. Finally, endothelial cells were generated and passaged every 3-5 days in EC Growth Medium MV2 with an additional 50 ng/ml VEGF. Before coculture as organoids or non-coculture as control, hPSCs-derived endothelial cells were purified by magnetic sorting using anti-CD31 (PECAM1) beads.

hPSCs differentiation towards macrophages

H9 hESCs expressing RFP (RFP-H9) were differentiated using a previously reported protocol.[98] RFP-H9 cells were dissociated with ReLeSR (STEMCELL Technologies) as small clusters onto Matrigel-coated 6-well plates at low density. The day after passaging, cells were cultured in IF9S medium supplemented with 50 ng/ml BMP-4, 15 ng/ml Activin A and 1.5 μM CHIR99021. After 2 days, medium was refreshed with IF9S medium supplemented with 50 ng/ml VEGF, 50 ng/ml bFGF, 50 ng/ml SCF (R&D Systems) and 10 μM SB431542 (Cayman Chemical). On day 5 and 7, medium was changed into IF9S medium supplemented with 50 ng/ml IL-6 (R&D Systems), 10 ng/ml IL-3 (R&D Systems), 50 ng/ml VEGF, 50 ng/ml bFGF, 50 ng/ml SCF and 50 ng/ml TPO (R&D Systems). On day 9, cells were dissociated with TrypLE (Life Technologies) and resuspended in IF9S medium supplemented with 50 ng/ml IL-6, 10 ng/ml IL-3 and 80 ng/ml M-CSF (R&D Systems). On day 13, medium was changed to IF9S medium supplemented with 50 ng/ml IL-6, 10 ng/ml IL-3 and 80 ng/ml M-CSF. On day 15, monocytes can be collected at this stage for further experiments. Otherwise, monocytes can be collected and plated on FBS-coated plates in IF9S medium supplemented with 80 ng/ml M-CSF to generate macrophages. IF9S medium was prepared according to previous publication.[98] All differentiation steps were cultured under normoxic conditions at 37 °C, 5% CO_2. Before coculture as organoids or non-coculture as control, hPSCs-derived macrophages were purified by magnetic sorting using anti-CD14 beads.

GeoMx transcriptomic and protein assays

Human control and COVID-19 pancreas samples were prepared as FFPE slides and applied to the NanoString GeoMx® Digital Spatial Profiler platform according to the manufacturer's instructions. In brief, slides from FFPE embedded pancreatic autopsy samples were prepared two weeks before experiments. Insulin (INS), Pan-ck (Pan Cytokeratin) and nuclear dye (TOTO™-3 Iodide) were used as morphology markers for selecting ROIs. We selected 6 ROIs in human islet areas, 3 ROIs in exocrine area and 3 ROIs in ductal area for each pancreas sample. The protein assays and transcriptomic assays were performed using adjacent sides. Data analysis was performed on GeoMx DSP software.

Construction of VMI organoids

The VMI organoids were constructed with hPSC-derived pancreatic endocrine cells, endothelial cells, and macrophages. Briefly, endocrine cells were dissociated with Accutase (Innovative Cell Technologies) at Day 16- 19, macrophages were dissociated with Accutase after day 19 of the differentiation procedure, and endothelial cells were dissociated with Trypsin 0.25% EDTA (THERMO FISHER) after day 10 of the differentiation procedure. The dissociated single cells were reaggregated with approximately 70-80% pancreatic endocrine cells, 10-20% endothelial cells, and approximately 2-5% macrophages in VMI organoid culture medium containing 80% pancreatic endocrine cells' stage 6 medium (MCDB 131 medium supplemented with 1.5 g/L sodium bicarbonate, 1× Glutamax, 20 mM glucose at final concentration, 2% BSA, 100 nM LDN193189, 1:200 ITS-X, 1 μM T3, 10 μM ALK5 inhibitor II, 10 μM zinc sulfate, 10 μg/ml of heparin, 100 nM gamma secretase inhibitor XX) plus 20% endothelial cells' medium (EC Growth Medium MV2 with an additional 50 ng/ml VEGF) using low-attach U plates. 48 hours later, the cells self-assembled into organoids. Subsequently, the medium was changed every two days.

SARS-CoV-2 infection

Infection assays on human islets or hESCs-derived VMI organoids were conducted in culture medium at specified multiplicity of infections (MOIs) and incubated at 37°C. Post-infection, at predetermined hours post-infection (hpi), the cells underwent triple PBS washes and a 60-minute fixation in 4% formaldehyde at room temperature. Culture medium alone served as control.

CVB4 infection

Human islets or hESCs-derived VMI organoids were infected with CVB4 E2 at 2×10^6 PFU/ml (2×10^4 PFU/organoid).[94] Human islets or VMI organoids were then incubated in a humidified incubator at 37°C with 5% CO_2 at predetermined hours post-infection (hpi), the cells underwent triple PBS washes and a 60-minute fixation in 4% formaldehyde at room temperature. Culture medium alone served as control.

Immunohistochemistry

Tissues were fixed overnight in 4% buffered formalin and transferred to 30% sucrose before being snap-frozen in O.C.T (Fisher Scientific, Pittsburgh, PA). Live organoids in culture were directly fixed in 4% paraformaldehyde for 30 min, followed with 60 min of permeabilization and blocking in PBS supplemented with 0.2% Triton X-100 and 5% horse serum. For immunofluorescence, cells or tissue sections were stained with primary antibodies at 4°C overnight and secondary antibodies at RT for 1h. The information for primary antibodies and secondary antibodies were provided in Table S3. Nuclei were counterstained by DAPI.

Single-cell RNA-seq data analysis of human islets upon SARS-CoV-2 or CVB4 infection

The 10X libraries were sequenced on the Illumina NovaSeq6000 sequencer with paired end reads (28 bp for read 1 and 90 bp for read 2). Subsequently, the sequencing data were primarily analyzed using the 10X cellranger pipeline v6.1.1 in a two-step process. In the initial step, cellranger *mkfastq* demultiplexed the samples and generated fastq files. In the subsequent step, cellranger *count* aligned the fastq files to a customized reference genome, extracting a gene expression UMI counts matrix for each library. The customized reference genome was constructed by integrating the 10X pre-built human reference GRCh38-2020-A, the SARS-CoV-2 virus genome, and the CVB4 virus genome using the cellranger *mkref*. The two virus genomes were obtained from the NCBI Nucleotide database with accession numbers NC_045512.2 (SARS-CoV-2) and AF311939.1 (CVB4).

We applied several filtering criteria, excluding cells with fewer than 500 or more than 6000 detected genes, cells with fewer than 1000 or more than 60000 detected transcripts, and cells with mitochondrial gene content exceeding 15%. Subsequently, we employed a deconvolution strategy[99] for normalizing gene expression UMI counts, utilizing the R scran package (v.1.14.1). Specifically, we initiated the process by pre-clustering cells with the *quickCluster* function. We then computed size factors per cell within each cluster, rescaled these factors by normalization between clusters using the *computeSumFactors* function, and normalized the UMI counts per cell by the size factors, followed by a logarithmic transform using the *normalize* function. We further normalized UMI counts across samples using the *multiBatchNorm* function in the R batchelor package (v1.2.1). We employed solo[100] v0.6 to identify doublets, which were subsequently excluded from the downstream analysis.

We identified highly variable genes using the *FindVariableFeatures* function in the R Seurat package v3.1.0, selecting the top 3000 variable genes after excluding mitochondria genes, ribosomal genes, viral genes, and dissociation-related genes. The list of dissociation-related genes, originally built on mouse data, was converted to human ortholog genes using Ensembl BioMart. Cells from multiple samples were aligned based on their mutual nearest neighbors (MNNs)[101] using the *fastMNN* function in the R batchelor package v1.2.1. This involved performing principal component analysis (PCA) on the highly variable genes and then correcting the principal components (PCs) according to their MNNs. We chose the corrected top 50 PCs for downstream visualization and clustering analysis.

Uniform Manifold Approximation and Projection (UMAP) dimensional reduction were executed using the *RunUMAP* function in the R Seurat package, with the number of neighboring points set to 30 and the training epochs set to 4000. Cells were clustered into thirteen clusters by constructing a shared nearest neighbor graph and grouping cells of similar transcriptome profiles using the *FindNeighbors* function and *FindClusters* function (resolution set to 0.2) in the R Seurat package. After reviewing the clusters, we merged them into nine clusters representing acinar cells, α cells, β cells, δ cells, ductal cells, mesenchymal cells, PP cells, immune cells, and endothelial cells for further analysis. Marker genes for the merged nine clusters were identified by performing differential expression analysis between cells inside and outside each cluster using the *FindMarkers* function in the R Seurat package. The expressions of cell type markers within each cell population were depicted through violin plots, utilizing the *VlnPlot* function in the R Seurat package. The expression of CVB4-polyprotein were visualized either through UMAP plot, employing the Seurat *DimPlot* function, or via jitter plot created with R ggplot2 package v3.2.1.

To assess cell death associated pathways within varied cell types following SARS-CoV-2 infection, we compared gene expressions in α cells, β cells, δ cells, mesenchymal cells and endothelial cells between mock and SARS-CoV-2 infected conditions using the Wilcoxon rank-sum test via the *FindMarkers* function in the R Seurat package. Subsequently, we ordered the genes based on $\log_2$ fold change and performed gene set enrichment analysis on cell death associated pathways using the *GSEA* function in the R clusterProfiler[102] package v4.6.2. The expressions of pyroptosis pathway associated genes in β cells under mock and SARS-CoV-2 infected conditions were visualized using the *DotPlot* function in the R Seurat package. The expressions of HLA genes and autoantigen associated genes in β cells under mock, SARS-CoV-2 infection or CVB4 infection conditions were represented using violin plots generated with the *VlnPlot* function in the R Seurat package.

To investigate the immune cell population, we extracted immune cells and performed a sub-clustering analysis. Highly variable genes were identified using the *FindVariableFeatures* function in the R Seurat package, and the top 3000 variable genes were selected, excluding mitochondria genes, ribosomal genes, viral genes and dissociation-related genes. Cells from multiple samples were aligned using the *fastMNN* function in the R batchelor package, as described above. The top 50 corrected PCs were selected for UMAP dimensional reduction using the *RunUMAP* function in the R Seurat package, with the number of neighboring points set to 30 and training epochs setting to 200. The immune cell population was clustered into seven clusters using the *FindNeighbors* function and *FindClusters* function (resolution set to 0.8) in the R Seurat package. After reviewing these clusters, we merged them into five clusters representing macrophages, dendritic cells, immune progenitor cells, T cells and B cells.

UMAP and violin plots were generated to illustrate the cell clusters and highlight expressions of selected genes using the R ggplot2 package v3.2.1. Dot plots were generated to show gene expression changes in the mock and infected conditions using the *DotPlot* function in the R Seurat package.

Single-cell RNA-seq data analysis of VMI organoids

The 10X scRNA-seq libraries underwent sequencing on the Illumina NovaSeq6000 sequencer with pair-end reads (28 bp for read 1 and 90 bp for read 2). Subsequently, the sequencing data were primarily analyzed using the 10X cellranger pipeline v7.1.0 in a two-step process. In the initial step, cellranger *mkfastq* demultiplexed the samples and generated fastq files. In the subsequent step, cellranger *count* aligned the fastq files to the 10X pre-built human reference GRCh38-2020-A reference, extracting a gene expression UMI counts matrix for each library.

Several filtering criteria were applied, excluding cells with fewer than 300 or more than 9000 detected genes, cells with fewer than 600 or more than 75000 detected transcripts, and cells with mitochondrial gene content exceeding 10%. Doublet cells in each sample were identified, assuming a doublet rate 0.8% per 1000 recovered cells, as reported by 10X Genomics, using the R DoubletFinder[103] package v2.0.3. The doublet cells were subsequently excluded from downstream analysis.

We employed a deconvolution strategy[99] for normalizing gene expression UMI counts, utilizing the R scran (v.1.22.1), scuttle (v1.4.0) and batchelor (v1.10.0) packages. The process involved pre-clustering cells with the *quickCluster* function in the R scran package, computing size factors per cell within each cluster, rescaling these factors by normalization between clusters using the *computeSumFactors* function in the R scran package, normalizing the UMI counts per cell by the size factors, followed by a logarithmic transform using the *logNormCounts* function in the R scuttle package. Further normalization of UMI counts across samples was performed using the *multiBatchNorm* function in the R batchelor package. Cells from multiple samples were aligned based on their MNNs using the *quickCorrect* function in the R batchelor package. This involved identifying highly variable genes, performing PCA on the highly variable genes and then correcting the PCs according to their MNNs. The corrected top 50 PCs were chosen for downstream clustering analysis. The corrected gene expression values on variable genes were reconstructed based on the corrected PCs and were used for coembeding scRNA-seq and snATAC-seq data.

UMAP dimensional reduction were executed using the *RunUMAP* function in the R Seurat package[104] av4.1.0, with the number of neighboring points set to 30 and the training epochs set to 500. Cells were clustered into fourteen clusters by constructing a shared nearest neighbor graph and grouping cells of similar transcriptome profiles using the *FindNeighbors* function and *FindClusters* function (resolution set to 0.5) in the R Seurat package. After reviewing the clusters, we merged them into nine clusters representing acinar cells, α cells, β cells, δ cells, ductal cells, endocrine progenitor cells, endothelial cells, macrophages and proliferation cells for further analysis.

DE analysis was performed on β cells between VMI organoids and non-coculture cells, and between VMI organoids with and without proinflammatory macrophages using the Wilcoxon rank-sum test via the *FindMarkers* function in the R Seurat package. Volcano plots were generated to illustrate DE genes using the R ggplot2 package v3.4.2. Dot plots were generated to show gene expression changes in different clusters or conditions using the *DotPlot* function in the R Seurat package. Pie charts were utilized to visualize cell type compositions within VMI organoids using the R ggplot package v3.4.2.

To determine the mechanisms by which proinflammatory macrophages induce β cell pyropotosis, we conducted cell-cell interaction analysis between macrophage and β cell populations using the R CellChat[105] package v1.5.0. Bubble plots were generated to illustrate the communication probabilities mediated by ligand-receptor pairs in between macrophage and β cell populations using the *netVisual_bubble* function.

Single-nuclear ATAC-seq data analysis of VMI organoids

The 10X snATAC-seq libraries underwent sequencing on the Illumina NovaSeq6000 sequencer with pair-end reads (51bp for read 1 and 51bp for read 2). Subsequently, the sequencing data were primarily analyzed using the 10X cellranger-atac pipeline v2.1.0 in a two-step process. In the initial step, cellranger-atac *mkfastq* demultiplexed the samples and generated fastq files. In the subsequent step, cellranger-atac *count* aligned the fastq files to the 10X pre-built GRCh38 2020-A-2.0.0 reference, performed peak calling, and extracted a barcoded and aligned fragment file for each library.

We ultilized the R Signac[106] package v1.10.0 to analyze snATAC-seq data. Specifically, we created a common set of peaks across all samples using the *reduce* function and generated a peaks x cell matrix for each sample by quantifying the common peaks using the *FeatureMatrix* function. We applied several filtering criteria, excluding cells with fewer than 3000 or more than 30000 peaks detected, cells with fewer than 20% of reads in peaks, cells with more than 5% of reads in blacklist regions, cells with the ratio of mononucleosomal to nucleosome-free fragments greater than 4, and cells with TSS enrichment score smaller than 3. Term frequency-inverse document frequency (TF-IDF) normalization was performed using the *RunTFIDF* function. We selected the top-ranked peaks using the *FindTopFeatures* function and ran singular value decomposition (SVD) to obtain latent semantic indexing (LSI) components using the *RunSVD* function. The top 50 LSI components, excluding the first LSI component, were used for downstream clustering analysis.

We classified cells into the nine cell types based on clustering results from scRNA-seq data. This was achieved by quantifying gene expression activity from the snATAC-seq data using the *GeneActivity* function, identifying anchors between scRNA-seq and snATAC-seq data using the *FindTransferAnchors* function, and transferring the cell clustering labels from scRNA-seq to snATAC-seq data using the *TransferData* function.

We co-embeded the scRNA-seq and snATAC-seq cells in the same UMAP plot. This was done by imputing gene expressions for the snATAC-seq cells based on the corrected gene expression values from the scRNA-seq cells using the *TransferData* function, merging cells from scRNA-seq and snATAC-seq, scaling the expression values and performing PCA using the *ScaleData* and

RunPCA functions, and selecting the top 30 PCs for UMAP dimensional reduction using the *RunUMAP* function with the number of neighboring points setting to 30 and training epochs setting to 500.

UMAP plots were generated to illustrate the cell clusters using the R ggplot2 package v3.4.2. Aggregated chromatin accessibility signals were visualized for multiple groups of cells within a given genomic region using the *CoveragePlot* function. Chromatin accessibility signal for individual cells were visualized using the *TilePlot* function. Pie charts were utilized to visualize cell type compositions within VMI organoids using the R ggplot package v3.4.2.

Bulk RNA-seq data analysis

The libraries underwent sequencing with single-end 50 bps on the Illumina NovaSeq6000 sequencer. Raw sequencing reads in BCL format were processed through bcl2fastq 2.20 (Illumina) for FASTQ conversion and demultiplexing. After trimming the adaptors with cutadapt v1.18, the sequencing reads were mapped to the human GRCh37 reference by STAR[107] av2.5.2b. Read counts per gene were extracted using HTSeq-count v0.11.2,[108] and normalized through a regularized log transformation with the DESeq2 package v1.26.0.[109]

RNA-Seq

Total RNA was extracted in TRIzol (Invitrogen) and DNase I treated using Directzol RNA Miniprep kit (Zymo Research) according to the manufacturer's instructions. RNAseq libraries of polyadenylated RNA were prepared using the TruSeq RNA Library Prep Kit v2 (Illumina) or TruSeq Stranded mRNA Library Prep Kit (Illumina) according to the manufacturer's instructions. cDNA libraries were sequenced using an Illumina NextSeq 500 platform. The resulting single end reads were checked for quality (FastQC v0.11.5) and processed using the Digital Expression Explorer 2 (DEE2)[110] workflow. Adapter trimming was performed with Skewer (v0.2.2).[111] Further quality control done with Minion, part of the Kraken package.[112] The resultant filtered reads were mapped to human reference genome GRCh38 using STAR aligner[107] and gene-wise expression counts generated using the "-quantMode GeneCounts" parameter. BigWig files were generated using the bamCoverage function in deepTools2 (v.3.3.0).[113] After further filtering and quality control, R package edgeR[114] was used to calculate RPKM and Log_2 counts per million (CPM) matrices as well as perform differential expression analysis. Heatmap was generated using online tool: http://www.heatmapper.ca/.

QUANTIFICATION AND STATISTICAL ANALYSIS

n=3 independent biological replicates were used for all experiments unless otherwise indicated. *P*-values were calculated by unpaired two-tailed Student's t-test or one way ANOVA with a common control unless otherwise indicated. n.s. indicates a non-significant difference. *$p<0.05$, **$p<0.01$ and ***$p<0.001$.

Cell Stem Cell, Volume *31*

Supplemental Information

Human vascularized macrophage-islet organoids to model immune-mediated pancreatic β cell pyroptosis upon viral infection

Liuliu Yang, Yuling Han, Tuo Zhang, Xue Dong, Jian Ge, Aadita Roy, Jiajun Zhu, Tiankun Lu, J. Jeya Vandana, Neranjan de Silva, Catherine C. Robertson, Jenny Z. Xiang, Chendong Pan, Yanjie Sun, Jianwen Que, Todd Evans, Chengyang Liu, Wei Wang, Ali Naji, Stephen C.J. Parker, Robert E. Schwartz, and Shuibing Chen

Figure S1

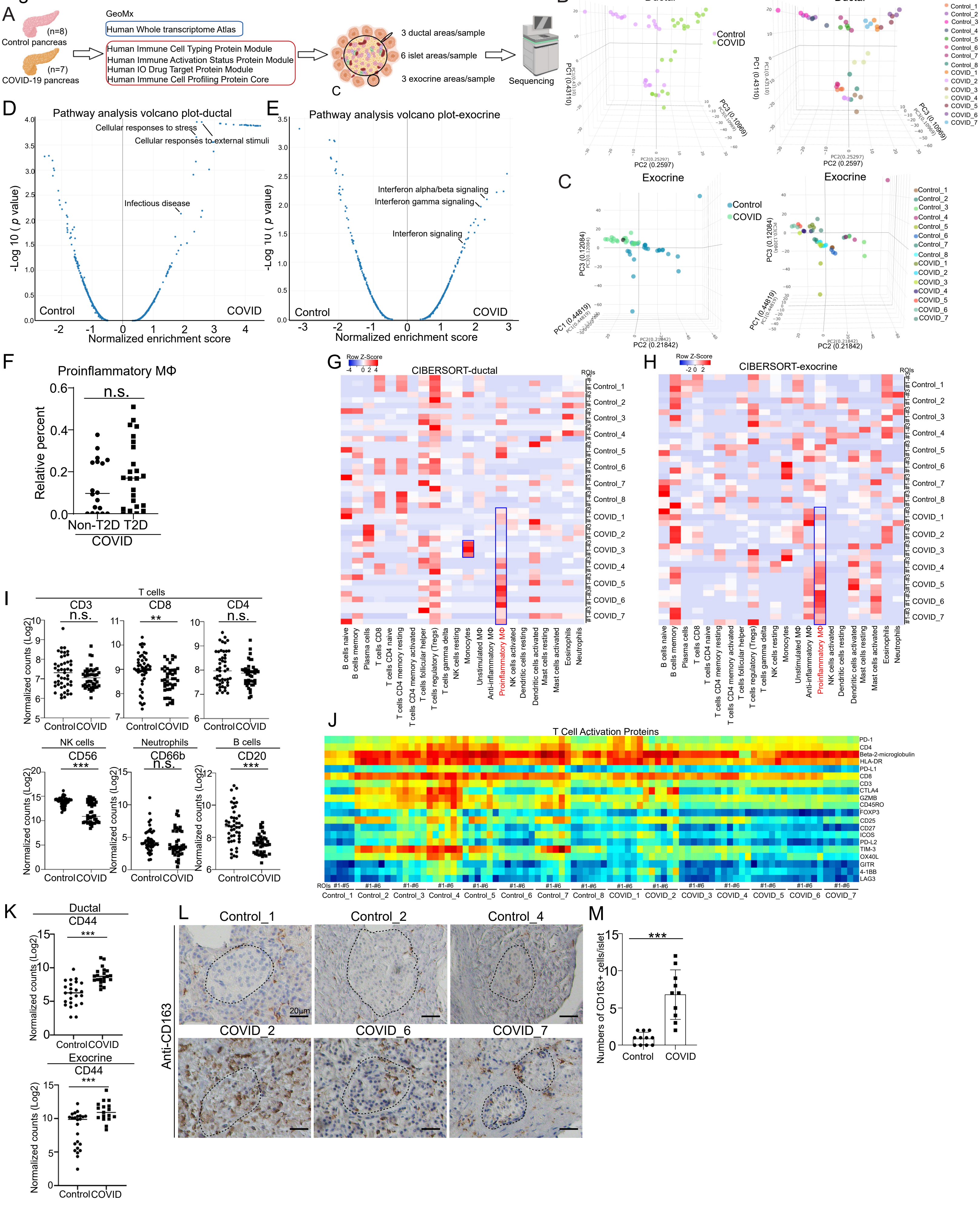

A
GeoMx
Human Whole transcriptome Atlas
Human Immune Cell Typing Protein Module
Human Immune Activation Status Protein Module
Human IO Drug Target Protein Module
Human Immune Cell Profiling Protein Core
Control pancreas (n=8)
COVID-19 pancreas (n=7)
3 ductal areas/sample
6 islet areas/sample
3 exocrine areas/sample
Sequencing
B Ductal
C Exocrine
D Pathway analysis volcano plot-ductal
Cellular responses to stress
Cellular responses to external stimuli
Infectious disease
Normalized enrichment score
-Log 10 (p value)
E Pathway analysis volcano plot-exocrine
Interferon alpha/beta signaling
Interferon gamma signaling
Interferon signaling
F Proinflammatory MΦ
Relative percent
n.s.
Non-T2D T2D
COVID
G CIBERSORT-ductal
H CIBERSORT-exocrine
Row Z-Score
I T cells CD3 CD8 CD4
NK cells CD56
Neutrophils CD66b
B cells CD20
Normalized counts (Log2)
J T Cell Activation Proteins
PD-1
CD4
Beta-2-microglobulin
HLA-DR
PD-L1
CD8
CD3
CTLA4
GZMB
CD45RO
FOXP3
CD25
CD27
ICOS
PD-L2
TIM-3
OX40L
GITR
4-1BB
LAG3
K Ductal CD44
Exocrine CD44
L Control_1 Control_2 Control_4
COVID_2 COVID_6 COVID_7
Anti-CD163
20μm
M Numbers of CD163+ cells/islet
Control COVID

SUPPLEMENTAL INFORMATION

Figure S1. GeoMx spatial transcriptomics and protein analysis of pancreatic autopsy samples from control and COVID-19 subjects. Related to Figure 1.

(A) Schematic representation of GeoMx spatial transcriptomics and protein analysis.

(B and C) 3D PCA plots of GeoMx whole transcriptome sequencing data from ROIs of human ductal (B) and exocrine (C) areas of COVID-19 (N=7) and control (N=8) pancreatic autopsy samples.

(D and E) Volcano plot highlighting the pathways enriched in transcriptome sequencing data from ROIs of human ductal (D) and exocrine (E) areas of COVID-19 (N=7) and control (N=8) pancreatic autopsy samples.

(F) Relative percent of proinflammatory macrophages from the CIBERSORT analysis of immune cells (LM22) using the GeoMx whole transcriptome sequencing data of human islet areas in non-T2D or T2D COVID-19 pancreatic autopsy samples. Each dot represents one count in each ROI.

(G) Heatmap of the CIBERSORT analysis of immune cells (LM22) using the GeoMx whole transcriptome sequencing data from ROIs of human ductal areas of COVID-19 (N=7) and control (N=8) pancreatic autopsy samples.

(H) Heatmap of the CIBERSORT analysis of immune cells (LM22) using the GeoMx whole transcriptome sequencing data from ROIs of human exocrine areas of COVID-19 (N=7) and control (N=8) pancreatic autopsy samples.

(I) Normalized counts (Log2) of immune cell markers, including CD3, CD8 and CD4 for T cells, CD56 for NK cells, CD66b for neutrophils and CD20 for B cells, in ROIs of the islet areas of control (N=8) and COVID-19 (N=7) pancreatic autopsy samples. Each dot represents one count in each ROI.

(J) Heatmap of the proteins related to T cell activation examined by GeoMx protein assay from ROIs of human islet areas of COVID-19 (N=7) and control (N=8) pancreatic autopsy samples.

(K) Normalized counts (Log2) of CD44 in the ductal or exocrine areas of control (N=8) and COVID-19 (N=7) pancreatic autopsy samples. Each dot represents one count in each ROI.

(L and M) Immunohistochemistry staining (L) and quantification (M) of CD163 in COVID-19 (N=3) and control (N=3) pancreatic autopsy samples. Dotted lines encircled the regions of the islets. Scale bar=20 μm.

P values were calculated by unpaired two-tailed Student's t test. n.s., no significance, $^{**}P < 0.01$, $^{***}P < 0.001$.

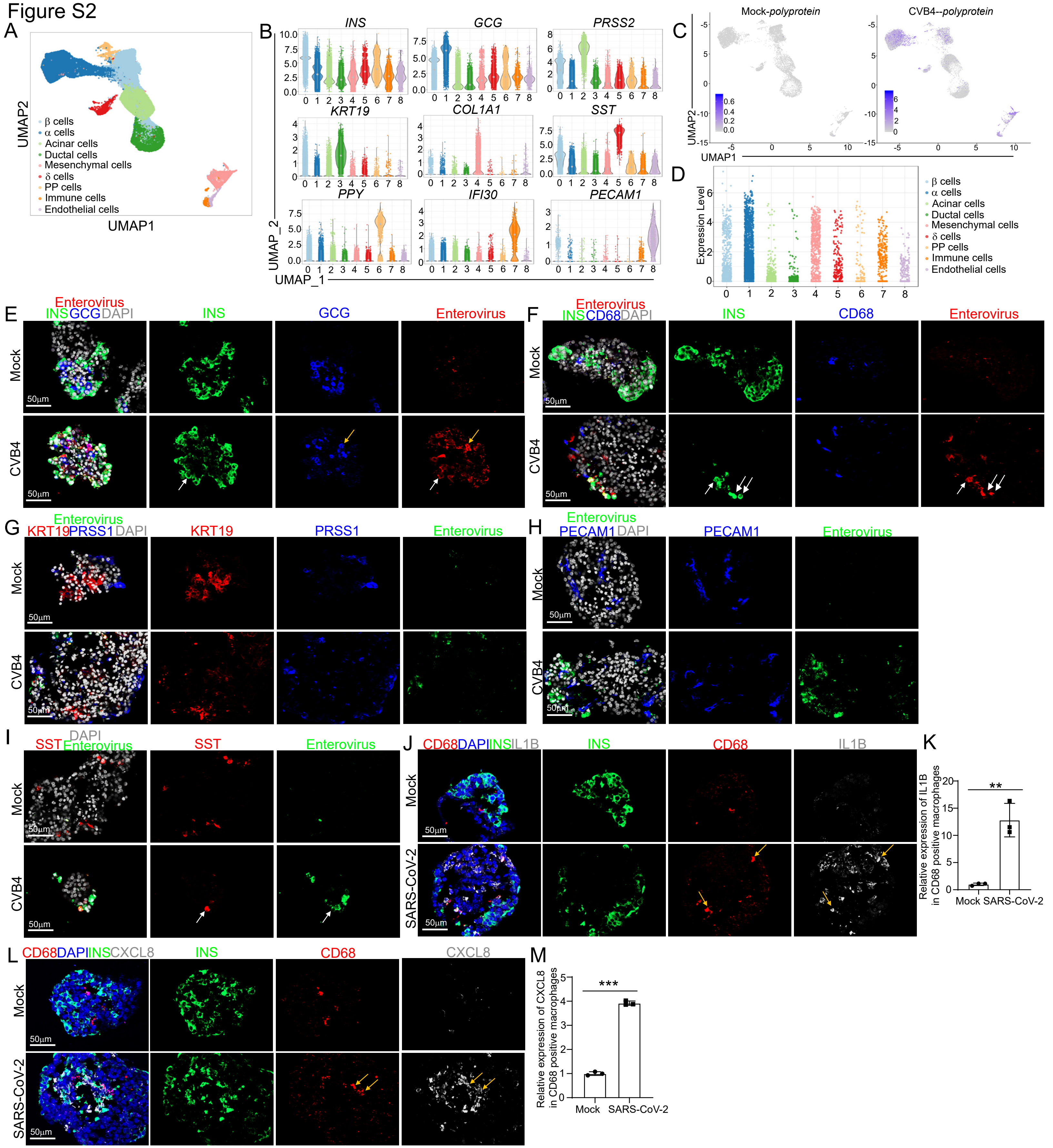

Figure S2
A
UMAP2
UMAP1
β cells
α cells
Acinar cells
Ductal cells
Mesenchymal cells
δ cells
PP cells
Immune cells
Endothelial cells
B
INS
GCG
PRSS2
KRT19
COL1A1
SST
PPY
IFI30
PECAM1
UMAP_2
UMAP_1
C
Mock-polyprotein
CVB4--polyprotein
UMAP2
UMAP1
D
Expression Level
E
Enterovirus
INS GCG DAPI
INS
GCG
Enterovirus
Mock
CVB4
50μm
F
Enterovirus
INS CD68 DAPI
INS
CD68
Enterovirus
G
Enterovirus
KRT19 PRSS1 DAPI
KRT19
PRSS1
Enterovirus
H
Enterovirus
PECAM1 DAPI
PECAM1
Enterovirus
I
SST DAPI Enterovirus
SST
Enterovirus
J
CD68 DAPI INS IL1B
INS
CD68
IL1B
SARS-CoV-2
K
Relative expression of IL1B in CD68 positive macrophages
**
Mock
SARS-CoV-2
L
CD68 DAPI INS CXCL8
INS
CD68
CXCL8
M
Relative expression of CXCL8 in CD68 positive macrophages
***
Mock
SARS-CoV-2

Figure S2. CVB4 infects human islets and induces activation of proinflammatory macrophages. Related to Figure 2.

(A) UMAP of human islets exposed to mock, SARS-CoV-2 (MOI=1) or CVB4 ($2x10^6$ PFU/ml) viruses.

(B) Violin plot of cell markers of each cell population, including *INS, GCG, PRSS2, KRT19*, *COL1A1*, *SST*, *PPY*, *IFI30*, and *PECAM1*.

(C) UMAP showed the expression of CVB4-*polyprotein* in human islets exposed to mock or CVB4 ($2x10^6$ PFU/ml) virus.

(D) Jitter plot showed the expression of CVB4-*polyprotein* in human islets exposed to mock or CVB4 ($2x10^6$ PFU/ml) virus.

(E) Confocal images of Enterovirus (CVB4) antigen expression in INS^+ β cells, GCG^+ α cells of human islets exposed to mock or CVB4 ($2x10^6$ PFU/ml). The white arrows highlight the co-localization of INS and Enterovirus antigen. The yellow arrows highlight the co-localization of GCG and Enterovirus antigen. Scale bar= 50 µm.

(F) Confocal images of Enterovirus (CVB4) antigen expression in INS^+ β cells, $CD68^+$ macrophages of human islets exposed to mock or CVB4 ($2x10^6$ PFU/ml). The white arrows highlight the co-localization of INS and Enterovirus antigen. Scale bar= 50 µm.

(G) Confocal images of Enterovirus (CVB4) antigen expression in KRT19$^+$ ductal cells, PRSS1$^+$ acinar cells of human islets exposed to mock or CVB4 (2x10^6 PFU/ml). Scale bar= 50 µm.

(H) Confocal images of Enterovirus (CVB4) antigen expression in PECAM1$^+$ endothelial cells of human islets exposed to mock or CVB4 (2x10^6 PFU/ml). Scale bar= 50 µm.

(I) Confocal images of Enterovirus (CVB4) antigen expression in SST$^+$ δ cells of human islets exposed to mock or CVB4 (2x10^6 PFU/ml). The white arrows highlight the co-localization of SST and Enterovirus antigen. Scale bar= 50 µm.

(J and K) Confocal images (J) and quantification (K) of IL1B expression in CD68$^+$ macrophages of human islets exposed to mock or SARS-CoV-2 (MOI=0.5). The yellow arrows highlight the co-localization of CD68 and IL1B. Scale bar= 50 µm.

(L and M) Confocal images (L) and quantification (M) of CXCL8 expression in CD68$^+$ macrophages of human islets exposed to mock or SARS-CoV-2 (MOI=0.5). The yellow arrows highlight the co-localization of CD68 and CXCL8. Scale bar= 50 µm.

n=3 independent biological replicates. Data was presented as mean ± STDEV. *P* values were calculated by unpaired two-tailed Student's t test. $^{**}P < 0.01$, $^{***}P < 0.001$.

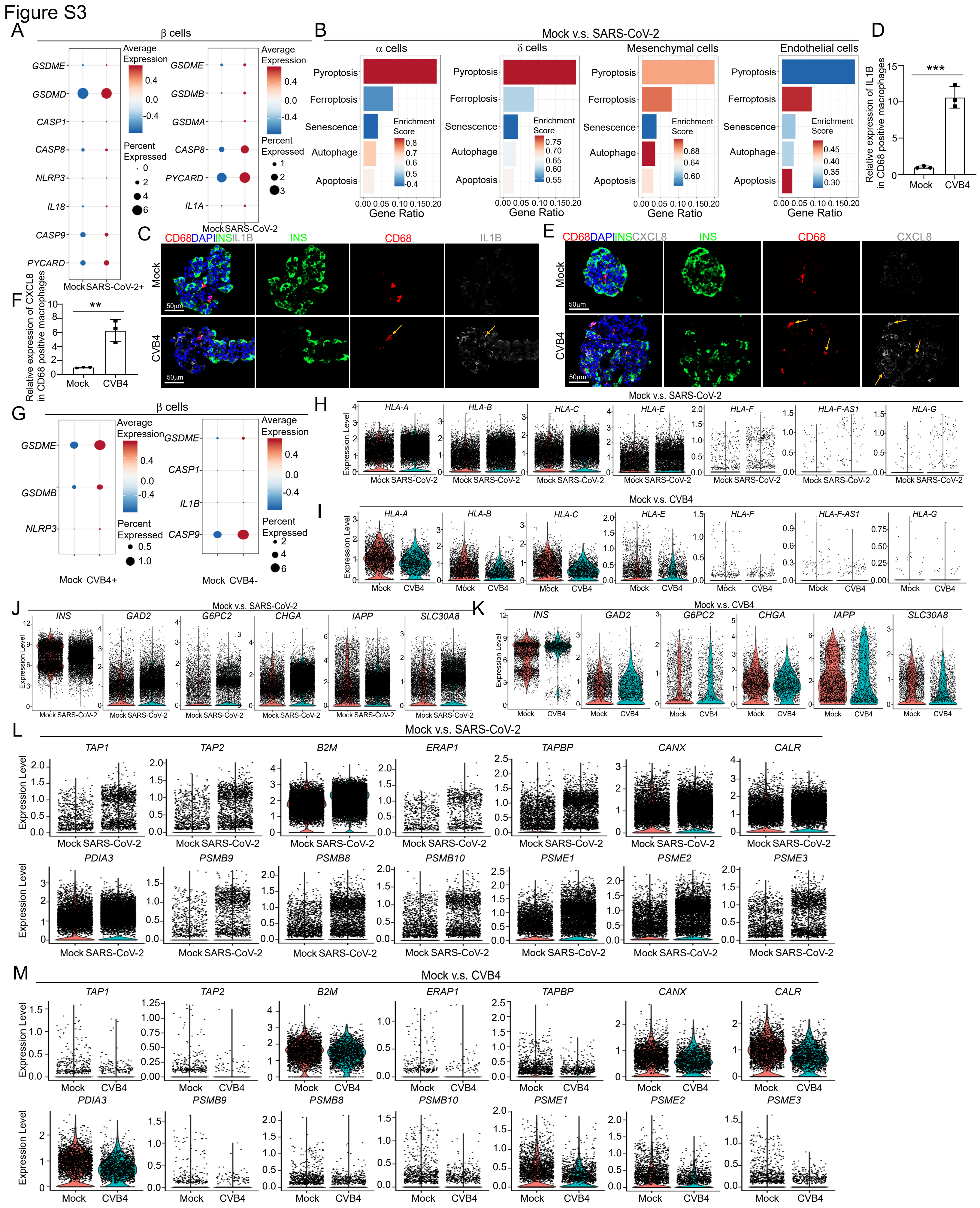
Figure S3
A
β cells
GSDME
GSDMD
CASP1
CASP8
NLRP3
IL18
CASP9
PYCARD
GSDMB
GSDMA
IL1A
Average Expression
Percent Expressed
Mock SARS-CoV-2+
Mock SARS-CoV-2
B
Mock v.s. SARS-CoV-2
α cells
δ cells
Mesenchymal cells
Endothelial cells
Pyroptosis
Ferroptosis
Senescence
Autophage
Apoptosis
Enrichment Score
Gene Ratio
C
CD68DAPIINSIL1B
INS
CD68
IL1B
Mock
CVB4
50μm
D
Relative expression of IL1B in CD68 positive macrophages
***
E
CD68DAPIINSCXCL8
CXCL8
F
Relative expression of CXCL8 in CD68 positive macrophages
**
G
β cells
Mock CVB4+
Mock CVB4-
H
Mock v.s. SARS-CoV-2
HLA-A
HLA-B
HLA-C
HLA-E
HLA-F
HLA-F-AS1
HLA-G
Expression Level
I
Mock v.s. CVB4
J
Mock v.s. SARS-CoV-2
INS
GAD2
G6PC2
CHGA
IAPP
SLC30A8
K
Mock v.s. CVB4
L
Mock v.s. SARS-CoV-2
TAP1
TAP2
B2M
ERAP1
TAPBP
CANX
CALR
PDIA3
PSMB9
PSMB8
PSMB10
PSME1
PSME2
PSME3
M
Mock v.s. CVB4

Figure S3. Single cell RNA-seq analysis of human islets upon CVB4 or SARS-CoV-2 exposure. Related to Figure 2.

(A) Dot plot analysis of pyroptosis pathway associated genes in virus$^+$ β cell cluster and virus$^-$ β cell cluster of human islets upon mock or SARS-CoV-2 exposure (MOI=1).

(B) Pathway enrichment analysis of cell death pathways in α, δ, mesenchymal and endothelial cell clusters of human islets exposed to mock or SARS-CoV-2 (MOI=1).

(C and D) Confocal images (C) and quantification (D) of IL1B expression in CD68$^+$ macrophages of human islets exposed to mock or CVB4 ($2x10^6$ PFU/ml). The yellow arrows highlight the co-localization of CD68 and IL1B. Scale bar= 50 µm.

(E and F) Confocal images (E) and quantification (F) of CXCL8 expression in CD68$^+$ macrophages of human islets exposed to mock or CVB4 ($2x10^6$ PFU/ml). The yellow arrows highlight the co-localization of CD68 and CXCL8. Scale bar= 50 µm.

(G) Dot plot analysis of pyroptosis pathway associated genes in virus$^+$ β cell cluster and virus$^-$ β cell cluster of human islets upon mock or CVB4 ($2x10^6$ PFU/ml).

(H) Violin plot of the expression of *HLA* genes in the β cell cluster of human islets exposed to mock or SARS-CoV-2 (MOI=1).

(I) Violin plot of the expression of *HLA* genes in the β cell cluster of human islets exposed to mock or CVB4 (2x10^6 PFU/ml).

(J) Violin plot of the expression of autoantigen genes in the β cell cluster of human islets exposed to mock or SARS-CoV-2 (MOI=1).

(K) Violin plot of the expression of autoantigen genes in the β cell cluster of human islets exposed to mock or CVB4 (2x10^6 PFU/ml).

(L) Violin plot of the expression of antigen presentation associated genes in the β cell cluster of human islets exposed to mock or SARS-CoV-2 (MOI=1).

(M) Violin plot of the expression of antigen presentation genes in the β cell cluster of human islets exposed to mock or CVB4 (2x10^6 PFU/ml).

n=3 independent biological replicates. Data was presented as mean ± STDEV. *P* values were calculated by unpaired two-tailed Student's t test. $^{**}P < 0.01$, $^{***}P < 0.001$.

Figure S4

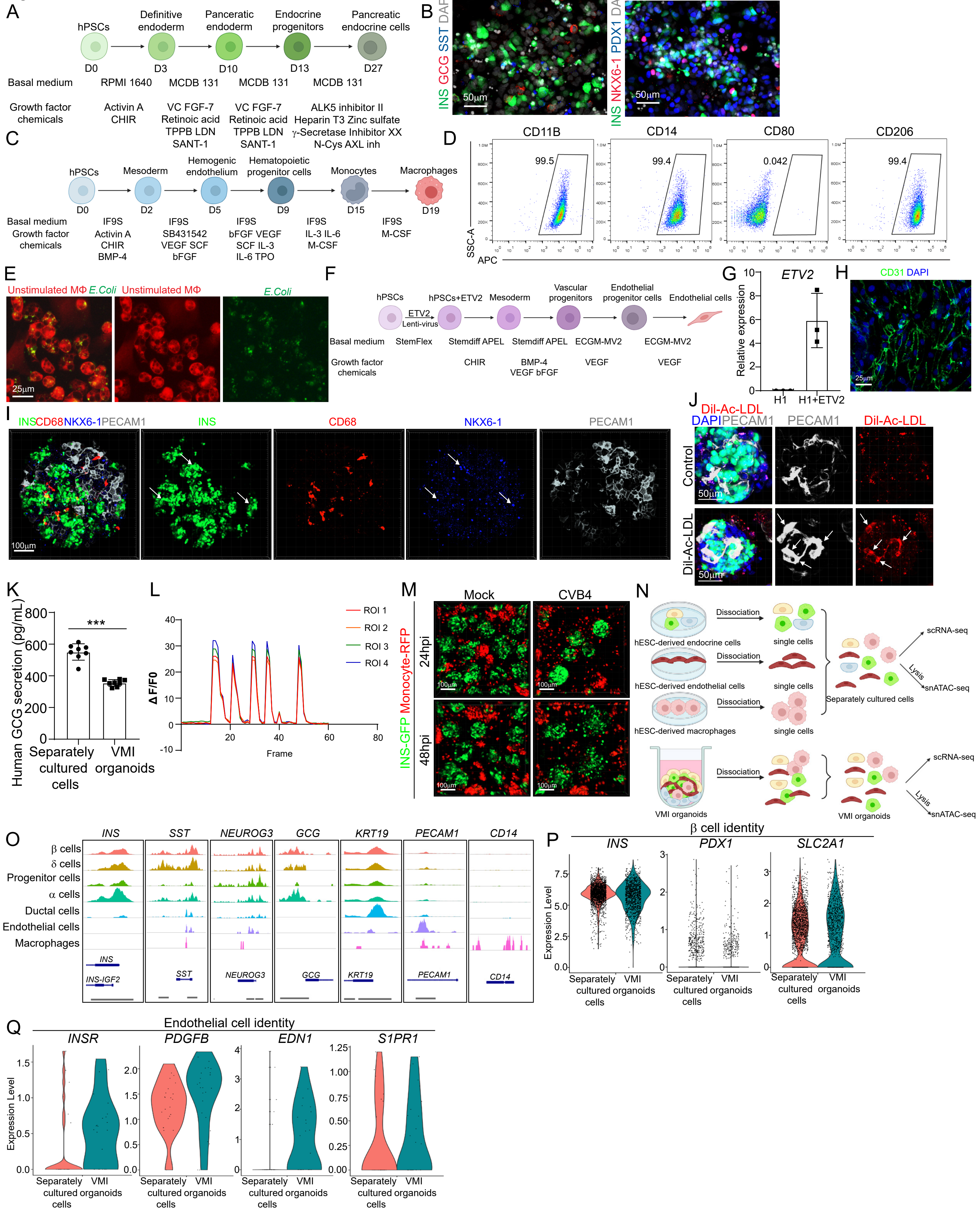
A
hPSCs
Definitive endoderm
Pancreatic endoderm
Endocrine progenitors
Pancreatic endocrine cells
D0
D3
D10
D13
D27
Basal medium
RPMI 1640
MCDB 131
MCDB 131
MCDB 131
Growth factor chemicals
Activin A CHIR
VC FGF-7 Retinoic acid TPPB LDN SANT-1
VC FGF-7 Retinoic acid TPPB LDN SANT-1
ALK5 inhibitor II Heparin T3 Zinc sulfate γ-Secretase Inhibitor XX N-Cys AXL inh
B
INS GCG SST DAPI
INS NKX6-1 PDX1 DAPI
50μm
C
hPSCs
Mesoderm
Hemogenic endothelium
Hematopoietic progenitor cells
Monocytes
Macrophages
D0
D2
D5
D9
D15
D19
Basal medium Growth factor chemicals
IF9S Activin A CHIR BMP-4
IF9S SB431542 VEGF SCF bFGF
IF9S bFGF VEGF SCF IL-3 IL-6 TPO
IF9S IL-3 IL-6 M-CSF
IF9S M-CSF
D
CD11B
CD14
CD80
CD206
99.5
99.4
0.042
99.4
SSC-A
APC
E
Unstimulated MΦ E.Coli
Unstimulated MΦ
E.Coli
25μm
F
hPSCs
ETV2 Lenti-virus
hPSCs+ETV2
Mesoderm
Vascular progenitors
Endothelial progenitor cells
Endothelial cells
Basal medium
StemFlex
Stemdiff APEL
Stemdiff APEL
ECGM-MV2
ECGM-MV2
Growth factor chemicals
CHIR
BMP-4 VEGF bFGF
VEGF
VEGF
G
ETV2
Relative expression
H1
H1+ETV2
H
CD31 DAPI
25μm
I
INS CD68 NKX6-1 PECAM1
INS
CD68
NKX6-1
PECAM1
100μm
J
Dil-Ac-LDL DAPI PECAM1
PECAM1
Dil-Ac-LDL
Control
Dil-Ac-LDL
50μm
K
Human GCG secretion (pg/mL)
***
Separately cultured cells
VMI organoids
L
ROI 1
ROI 2
ROI 3
ROI 4
ΔF/F0
Frame
M
Mock
CVB4
24hpi
48hpi
INS-GFP Monocyte-RFP
100μm
N
hESC-derived endocrine cells
hESC-derived endothelial cells
hESC-derived macrophages
Dissociation
single cells
Separately cultured cells
scRNA-seq
Lysis
snATAC-seq
VMI organoids
O
INS
SST
NEUROG3
GCG
KRT19
PECAM1
CD14
β cells
δ cells
Progenitor cells
α cells
Ductal cells
Endothelial cells
Macrophages
INS-IGF2
P
β cell identity
INS
PDX1
SLC2A1
Expression Level
Separately cultured cells
VMI organoids
Q
Endothelial cell identity
INSR
PDGFB
EDN1
S1PR1
Expression Level
Separately cultured cells
VMI organoids

Figure S4. Construction and characterization of hPSC-derived VMI organoids. Related to Figure 3 and Figure 4.

(A) Schematic illustration of directed differentiation of hPSCs to pancreatic endocrine cells. At day 16, we detected INS-GFP^{+} cells. Then, early stage 6 cells (day 16-day19) were co-cultured with macrophages and endothelial cells or culture separately for 7-14 days.

(B) Confocal images of hPSC-derived pancreatic endocrine cells stained with antibodies against INS, GCG, NKX6-1, PDX1 and SST. Scale bar= 50 µm.

(C) Schematic illustration of directed differentiation of hESCs to macrophages.

(D) Flow cytometry analysis of hPSC-derived unstimulated macrophages stained with antibodies against CD11B, CD14, CD80 and CD206.

(E) Confocal images of hPSC-derived macrophages engulfing GFP labeled *E.Coli*. Macrophages: RFP; *E.Coli: Green*. Scale bar= 25 µm.

(F) Schematic illustration of directed differentiation of hPSCs to endothelial cells.

(G) qRT-PCR analysis to examine the expression level of *ETV2* in H1 hPSCs following forced expression of ETV2 or control. Data was normalized to β-actin.

(H) Confocal images of hPSC-derived endothelial cells stained with antibodies against PECAM1 (CD31) and DAPI. Scale bar= 25 µm.

(I) Composite Z-stack confocal images of VMI organoids at day 14 after reaggregation stained with antibodies against INS, CD68, NKX6-1 and PECAM1 (CD31). The white arrows highlight the co-localization of INS and NKX6-1. Scale bar= 100 µm.

(J) Confocal images of VMI organoids stained with antibodies against PECAM1 (CD31) and Dil-Ac-LDL. Scale bar= 50 µm.

(K) ELISA assay showed the secretion of GCG in VMI organoids and separately cultured endocrine cells.

(L) Quantification of calcium signaling in VMI organoids upon high glucose stimulation. High glucose: 20 mM D-glucose. Each frame was captured every 500ms.

(M) Live cell imaging of VI organoids and monocytes exposed to mock or CVB4 ($2x10^6$ PFU/ml) at 24 hpi and 48 hpi. Scale bar= 100 µm.

(N) Schematic illustration of the sample preparation for scRNA-seq and snATAC-seq.

(O) Chromatin accessibility signals of cell markers for each cluster as analyzed using snATAC-seq.

(**P)** Violin plot analysis of β cell associated genes in β cell cluster of VMI organoids at day 7 after reaggregation and separately cultured cells as analyzed by scRNA-seq.

(**Q)** Violin plot analysis of endothelial cell associated genes in endothelial cell cluster of VMI organoids at day 7 after reaggregation and separately cultured cells as analyzed by scRNA-seq.

n=3 independent biological replicates. Data was presented as mean ± STDEV. *P* values were calculated by unpaired two-tailed Student's t test. ***$P < 0.001$.

Figure S5

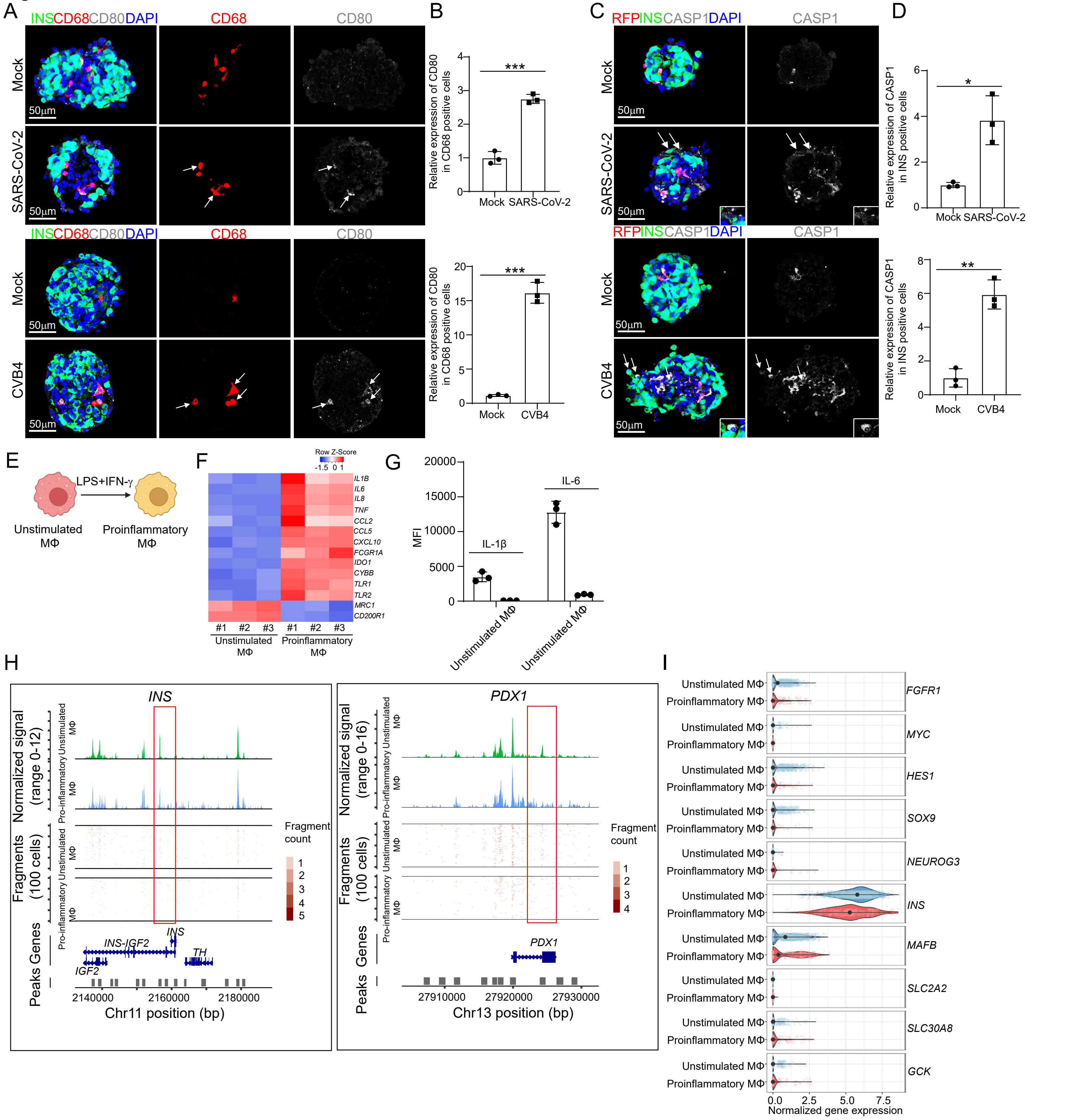
A
INSCD68CD80DAPI
CD68
CD80
Mock
SARS-CoV-2
CVB4
50µm
B
Relative expression of CD80 in CD68 positive cells
***
Mock
SARS-CoV-2
CVB4
C
RFPINSCASP1DAPI
CASP1
D
Relative expression of CASP1 in INS positive cells
*
**
E
LPS+IFN-γ
Unstimulated MΦ
Proinflammatory MΦ
F
Row Z-Score
IL1B
IL6
IL8
TNF
CCL2
CCL5
CXCL10
FCGR1A
IDO1
CYBB
TLR1
TLR2
MRC1
CD200R1
#1 #2 #3
Unstimulated MΦ
Proinflammatory MΦ
G
MFI
IL-1β
IL-6
Unstimulated MΦ
H
INS
PDX1
Normalized signal (range 0-12)
Normalized signal (range 0-16)
Fragments (100 cells)
Fragment count
Unstimulated MΦ
Pro-inflammatory MΦ
Genes
Peaks
INS-IGF2
IGF2
TH
Chr11 position (bp)
Chr13 position (bp)
I
FGFR1
MYC
HES1
SOX9
NEUROG3
INS
MAFB
SLC2A2
SLC30A8
GCK
Normalized gene expression

Figure S5. Activation of proinflammatory macrophages and β cell pyroptosis were detected in hPSC-derived VMI organoids exposed to viruses. Related to Figure 5.

(A and B) Confocal images (A) and quantification (B) of hPSC-derived VMI organoids exposed to viruses or mock conditions stained with antibodies against INS, CD68 and CD80 (SARS-CoV-2: MOI=0.5; CVB4: $2x10^6$ PFU/ml). Scale bar= 50 µm. The white arrows highlight the $CD68^+CD80^+$ cells.

(C and D) Confocal images (C) and quantification (D) of hPSC-derived VMI organoids exposed to viruses or mock conditions stained with antibodies against INS and CASP1 (SARS-CoV-2: MOI=0.5; CVB4: $2x10^6$ PFU/ml). Scale bar= 50 µm. The white arrows highlight the INS^+CASP1^+ cells.

(E) Schematic illustration of the stimulation of macrophages to proinflammatory macrophages.

(F) Heatmap showing the expression of macrophage associated genes in hPSC-derived macrophages with or without 2 days treatment with 100 ng/ml LPS and 20 ng/ml IFN-γ.

(**G)** The secretion of cytokines, including IL-1β and IL-6 in the supernatant of hPSC-derived macrophages with or without 2 days treatment with 100 ng/ml LPS and 20 ng/ml IFN-γ.

(H) Chromatin accessibility signals of the β cluster of VMI organoids at day 7 after reaggregation containing unstimulated macrophages or proinflammatory macrophages as analyzed using snATAC-seq. The normalized signal shows the averaged frequency of sequenced DNA fragments within a genomic region. The fragment shows the frequency of sequenced fragments within a genomic region for individual cells.

(**I)** Jitter plot analysis of β cell dedifferentiation associated genes in β cell cluster of VMI organoids with unstimulated macrophages or proinflammatory macrophages at day 7 after reaggregation as analyzed by scRNA-seq.

n=3 independent biological replicates. Data was presented as mean ± STDEV. *P* values were calculated by unpaired two-tailed Student's t test. **P* < 0.05, ***P* < 0.01, ****P* < 0.001.

Figure S6

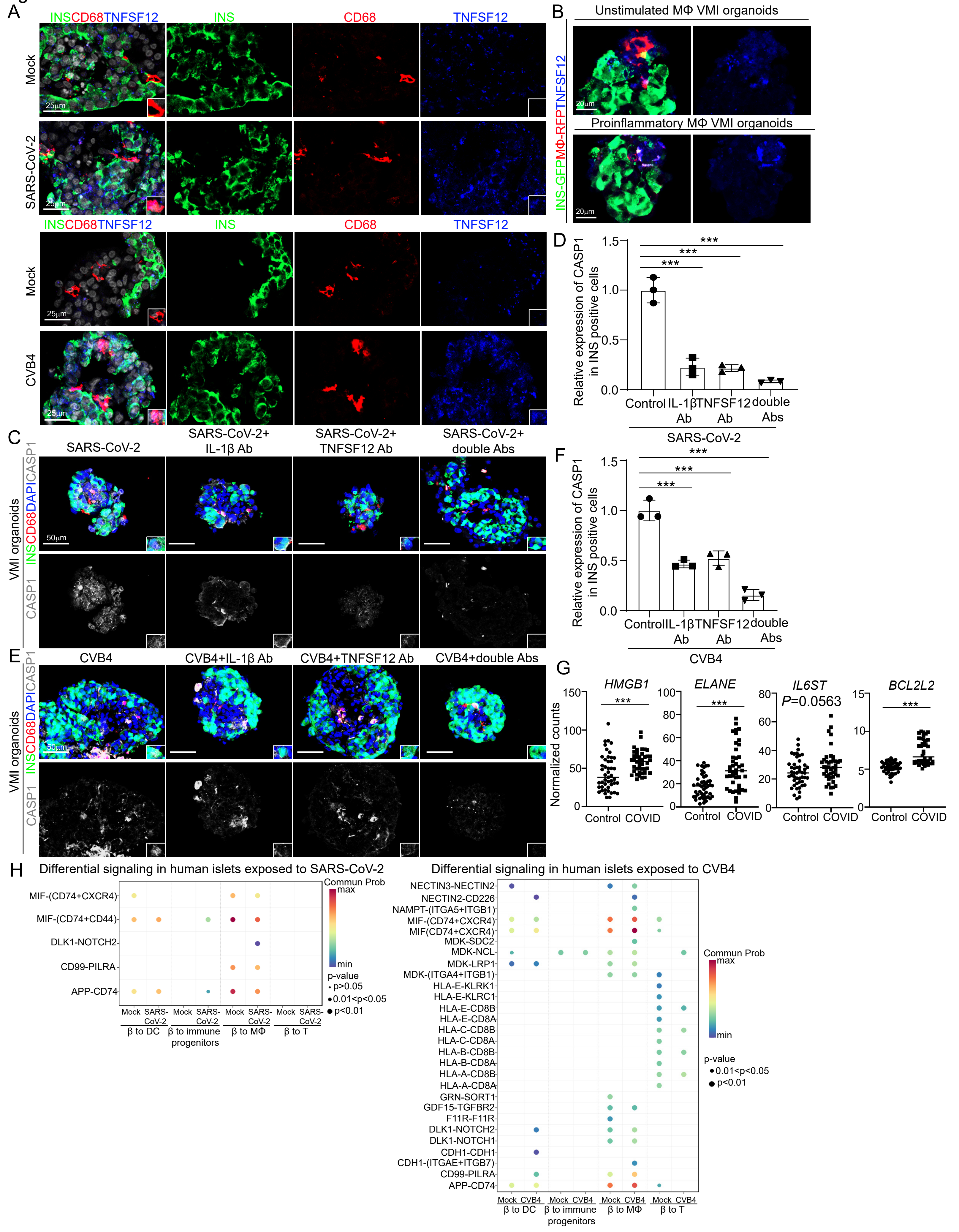
A
INSCD68TNFSF12
INS
CD68
TNFSF12
Mock
SARS-CoV-2
CVB4
25μm
B
Unstimulated MΦ VMI organoids
Proinflammatory MΦ VMI organoids
INS-GFPMΦ-RFPTNFSF12
20μm
C
SARS-CoV-2
SARS-CoV-2+ IL-1β Ab
SARS-CoV-2+ TNFSF12 Ab
SARS-CoV-2+ double Abs
VMI organoids
INSCD68DAPICASP1
CASP1
50μm
D
Relative expression of CASP1 in INS positive cells
1.5
1.0
0.5
0.0
***
Control
IL-1β Ab
TNFSF12 Ab
double Abs
SARS-CoV-2
E
CVB4
CVB4+IL-1β Ab
CVB4+TNFSF12 Ab
CVB4+double Abs
F
CVB4
G
HMGB1
ELANE
IL6ST
BCL2L2
P=0.0563
Normalized counts
Control
COVID
H
Differential signaling in human islets exposed to SARS-CoV-2
MIF-(CD74+CXCR4)
MIF-(CD74+CD44)
DLK1-NOTCH2
CD99-PILRA
APP-CD74
Commun Prob
max
min
p-value
p>0.05
0.01<p<0.05
p<0.01
Mock
SARS-CoV-2
β to DC
β to immune progenitors
β to MΦ
β to T
Differential signaling in human islets exposed to CVB4
NECTIN3-NECTIN2
NECTIN2-CD226
NAMPT-(ITGA5+ITGB1)
MIF-(CD74+CXCR4)
MIF(CD74+CXCR4)
MDK-SDC2
MDK-NCL
MDK-LRP1
MDK-(ITGA4+ITGB1)
HLA-E-KLRK1
HLA-E-KLRC1
HLA-E-CD8B
HLA-E-CD8A
HLA-C-CD8B
HLA-C-CD8A
HLA-B-CD8B
HLA-B-CD8A
HLA-A-CD8B
HLA-A-CD8A
GRN-SORT1
GDF15-TGFBR2
F11R-F11R
DLK1-NOTCH2
DLK1-NOTCH1
CDH1-CDH1
CDH1-(ITGAE+ITGB7)
CD99-PILRA
APP-CD74
Mock
CVB4

Figure S6. TNFSF12 expression in human islets exposed to CVB4 or SARS-CoV-2, and VMI organoids with proinflammatory macrophages. Related to Figure 6.

(A) Confocal images of TNFSF12 in human islets exposed to mock, CVB4 ($2x10^6$ PFU/ml) or SARS-CoV-2 (MOI=0.5). The inserts show a high magnification of cells. Scale bar= 25 µm.

(B) Confocal images of TNFSF12 in VMI organoids containing unstimulated or pro-inflammatory macrophages at day 7 after reaggregation. Scale bar= 20 µm.

(C and D) Confocal images (C) and quantification (D) of the CASP1 expression in INS^+ cells of VMI organoids exposed to SARS-CoV-2 (MOI=0.5) and treated with control, 10 µg/ml TNFSF12 blocking antibody, 5 µg/ml IL-1β blocking antibody or 10 µg/ml TNFSF12 + 5 µg/ml IL-1β blocking antibodies. The inserts show a high magnification of cells. Scale bar= 50 µm.

(E and F) Confocal images (E) and quantification (F) of the CASP1 expression in INS^+ cells of VMI organoids exposed to CVB4 ($2x10^6$ PFU/ml) and treated with control, 10 µg/ml TNFSF12 blocking antibody, 5 µg/ml IL-1β blocking antibody or 10 µg/ml TNFSF12 + 5 µg/ml IL-1β blocking antibodies. The inserts show a high magnification of cells. Scale bar= 50 µm.

(G) Normalized counts of pyroptosis associated genes expression in control or COVID-19 samples examined by GeoMx transcriptomic assays. Each dot represents one count in each ROI.

(H) Cell chat analysis showed the interactions from β cells to immune cell subpopulations, including DC cells, immune progenitors, T cells and macrophages in human islets exposed to SARS-CoV-2 (MOI=1) or CVB4 ($2x10^6$ PFU/ml).

n=3 independent biological replicates. Data was presented as mean ± STDEV. *P* values were calculated by one-way ANOVA with a common control. ***$P < 0.001$.

Table S1. Patient information. Related to Figures 1, 2, 6 and Figures S1, S2, S3, S6.

Sample name	Age	Sex	Pre-existing diabetes	Tissues	Obesity (BMI)	Family Hx	Laboratory data (WBC)	The onset and severity of the disease	Time to death from onset	Comorbidities	Glucose	Transport, and processing of these samples	Cause of death
Control_1	58	F	N	pancreas	24.2	Yes, Brother	7.6 - 12.2	N/A	N/A	N/A	60-325	Within 1 day	Anoxia
Control_2	64	M	N	pancreas	25.12	N/A	N/A	N/A	N/A	N/A	96-105	Within 1 day	Anoxia
Control_3	64	F	N	pancreas	20.92	N/A	N/A	N/A	N/A	N/A	131-242	Within 1 day	CVA
Control_4	66	F	N	pancreas	32.98	N/A	N/A	N/A	N/A	N/A	154-354	Within 1 day	CVA
Control_5	61	F	N	pancreas	29.85	N/A	N/A	N/A	N/A	N/A	195-724	Within 1 day	Anoxia
Control_6	60	F	T2D	pancreas	29.7	N/A	14.2 - 20.4	N/A	N/A	N/A	58-482	Within 1 day	Anoxia
Control_7	59	M	N	pancreas	21.32	N/A	N/A	N/A	N/A	N/A	106-344	Within 1 day	Trauma
Control_8	62	F	T2D	pancreas	37.01	N/A	N/A	N/A	N/A	N/A	76-407	Within 1 day	CVA
Control_9	58	F	N	islets	31.07	No	8.7 - 16.4	N/A	N/A	N/A	131-212	Within 1 day	CVA
Control_10	27	M	N	islets	25.3	No	4.5 - 9.4	N/A	N/A	N/A	128-520	Within 1 day	CAR
Control_11	39	M	N	islets	31.07	No	8 - 14.7	N/A	N/A	N/A	130-346	Within 1 day	Anoxia
Control_12	57	M	N	islets	24.5	Yes, Mom & Dad	6.7 - 8.9	N/A	N/A	N/A	104-241	Within 1 day	CVA
COVID_1	58	M	T2D	pancreas	29.19	No	29.1	Hospitalized not intubat	21 days	HTN	75-171	Within 1 day	COVID Pneumonia

								ed, Required O2					
COVID_2	64	M	T2D	pancreas	19.77	No	17.4	Hospitalized not intubated, Required O2	1 day	HTN	103-296	Within 2 days	Respiratory failure and pneumonia
COVID_3	75	F	N	pancreas	23.62	No	35.07	Hospitalized not intubated, Required O2	12 days	Multiple Myeloma in good control, H/O DVT and PE on AC	43-179	Within 1 day	COVID Pneumonia
COVID_4	81	F	N	pancreas	20.27	No	22.7	Hospitalized not intubated, Required O2	9 days	CAD, Gout, HTN, Hyperlipidemia, Dementia	133-221	Within 1 day	COVID Pneumonia
COVID_5	53	M	T2D	pancreas	30.63	No	21.3	Hospitalized not intubated, Required O2	47 days	HTN, CVA	81-292	Within 2 days	Non COVID Pneumonia
COVID_6	87	M	T2D	pancreas	21.03	No	6.8	Hospitalized not intubated, Required O2	5 days	HTN, T2DM, Dementia	50-307	Within 1 day	COVID Pneumonia
COVID_7	71	F	N	pancreas	22.76	Yes, Brother	19.3	Hospitalized, Intubated	29 days	HTN, AML in remission	118-254	Within 1 day	COVID Pneumonia

						& Mom							
COVID_8	55	M	N	pancreas	Not recorded	No	17.46	Hospitalized not intubated, Required O2	14 days	HTN, CAD, Hyperlipidemia	110-263	Within 2 days	Respiratory failure and pneumonia

Table S2. Antibodies used for immunocytochemistry, intracellular flow cytometry analysis. Related to STAR Methods.

Usage	Antibody	Clone #	Host	Catalog #	Vendor	Dilution
Immunostaining	Polyclonal Guinea Pig Anti-Insulin	Polyclonal	Guinea Pig	#A0564	Dako	1:500
Immunostaining	Glucagon Rabbit Ab	Polyclonal	Rabbit	#2760	Cell Signaling	1:1000
Immunostaining	Polyclonal Rabbit Anti-Somatostatin	Polyclonal	Rabbit	#A0566	Dako	1:1000
Immunostaining	Human CD31/PECAM-1 Antibody	Polyclonal	Sheep	#AF806	R&D Systems	1:1000
Immunostaining	Purified anti-human CD68 Antibody	Monoclonal	Mouse	# 333802	Biolegend	1: 100
Immunostaining	Cleaved Caspase-1 (Asp297)	Monoclonal	Rabbit	# 4199	Cell Signaling	1: 200
Immunostaining	hPDX-1 Affinity purified goat igG	Polyclonal	Goat	# AF2419	R&D Systems	1:500
Immunostaining	Nkx6.1 (D8O4R) Rabbit mAb	Monoclonal	Rabbit	#54551	Cell Signaling	1: 500
Flow Cytometry	APC anti-mouse/human CD11b Antibody	Monoclonal	Rat	#101212	Biolegend	1: 50
Flow Cytometry	APC anti-human CD206 (MMR) Antibody	Monoclonal	Mouse	#321109	Biolegend	1:50
Flow Cytometry	APC anti-human CD14	Monoclonal	Mouse	#301808	Biolegend	1:100
GeoMx	Insulin Monoclonal Antibody (ICBTACLS), Alexa Fluor™ 488	Monoclonal	Mouse	#53-9769-82	Thermo Fisher Scientific	1:200

GeoMx	Cytokeratin, pan Antibody (AE-1/AE-3) [DyLight 594]	Monoclonal	Mouse	# NBP2-33200DL594	Novus Biological	1:200
Immunostaining	caspase-1 Antibody (14F468)	Monoclonal	Mouse	#sc-56036	Santa Cruz	1:200
Immunostaining	Enterovirus (Concentrate)	Monoclonal	Mouse	#M7064	Dako	1:500
Immunostaining	CD163 (D6U1J) Rabbit mAb	Monoclonal	Rabbit	#93498	Cell Signaling	1:200
Immunostaining	Anti-PRSS1 antibody produced in rabbit	Polyclonal	Rabbit	#HPA063471	Sigma Aldrich	1:500
GeoMx	Purified anti-Cytokeratin 19	Monoclonal	Mouse	#628502	Biolegend	1:1000
Immunohistochemistry	Human B7-1/CD80 MAb (Clone 37711)	Monoclonal	Mouse	# MAB140-100	RnD	1:500
Immunostaining	Alexa Fluor 488 AffiniPure Donkey Anti-Guinea Pig IgG (H+L)	Polyclonal	Donkey	#706-545-148	Jackson Immunoresearch Labs	1:500
Immunostaining	Donkey anti-Mouse IgG (H+L) Highly Cross-Adsorbed Secondary Antibody, Alexa Fluor 594	Polyclonal	Donkey	#A-21203	Thermo Fisher Scientific	1:500
Immunostaining	Donkey anti-Rabbit IgG (H+L) Secondary Antibody, Alexa Fluor 594 conjugate	Polyclonal	Donkey	#A-21207	Thermo Fisher Scientific	1:500
Immunostaining	Donkey anti-Rabbit IgG (H+L) Secondary Antibody,	Polyclonal	Donkey	#A-31573	Thermo Fisher Scientific	1:500

	Alexa Fluor 647 conjugate					
Immunostaining	Donkey anti-Mouse IgG (H+L) Secondary Antibody, Alexa Fluor 647	Polyclonal	Donkey	#A-31571	Thermo Fisher Scientific	1:500
Immunostaining	Donkey anti-Goat IgG (H+L) Cross-Adsorbed Secondary Antibody, Alexa Fluor 647	Polyclonal	Donkey	#A-21447	Thermo Fisher Scientific	1:500
Immunostaining	Donkey anti-Sheep IgG (H+L) Cross-Adsorbed Secondary Antibody, Alexa Fluor 647	Polyclonal	Donkey	#A-21448	Thermo Fisher Scientific	1:500
Immunostaining	Donkey anti-Mouse IgG (H+L) Highly Cross-Adsorbed Secondary Antibody, Alexa Fluor Plus 405	Polyclonal	Donkey	#A48257	Thermo Fisher Scientific	1:500

Table S3. Primers used for qRT-PCR. Related to STAR Methods.

**Primer name**	**Sequence**
ACTB-Forward	*CGTCACCAACTGGGACGACA*
ACTB-Reverse	*CTTCTCGCGGTTGGCCTTGG*
ETV2-F	*GAAGGAGCCAAATTAGGCTTCT*
ETV2-R	*GAGCTTGTACCTTTCCAGCAT*
